# Which Probiotic Is the Most Effective for Treating Acute Diarrhea in Children? A Bayesian Network Meta-Analysis of Randomized Controlled Trials

**DOI:** 10.3390/nu13124319

**Published:** 2021-11-29

**Authors:** Zengbin Li, Guixian Zhu, Chao Li, Hao Lai, Xin Liu, Lei Zhang

**Affiliations:** 1 China-Australia Joint Research Center for Infectious Diseases, School of Public Health, Xi’an Jiaotong University Health Science Center, Xi’an 710061, China; ZengbinLi@stu.xjtu.edu.cn (Z.L.); xianxianshell@stu.xjtu.edu.cn (G.Z.); xjtu_haolai@stu.xjtu.edu.cn (H.L.); 2 Department of Epidemiology and Biostatistics, School of Public Health, Global Health Institute, Xi’an Jiaotong University Health Science Center, Xi’an 710061, China; lcxjtu@xjtu.edu.cn (C.L.); xinliu@xjtu.edu.cn (X.L.); 3 Melbourne Sexual Health Centre, Alfred Health, Melbourne, VIC 3053, Australia; 4 Central Clinical School, Faculty of Medicine, Nursing and Health Sciences, Monash University, Melbourne, VIC 3800, Australia; 5 Department of Epidemiology and Biostatistics, College of Public Health, Zhengzhou University, Zhengzhou 450001, China

**Keywords:** probiotics, acute diarrhea, children, network meta-analysis

## Abstract

Acute diarrhea is a major cause of morbidity and mortality in children under five. Probiotics are beneficial for treating acute diarrhea in children, but unclear which specific probiotic is the most effective. We performed a Bayesian network meta-analysis to examine the comparative effectiveness of probiotics. By searching EMBASE, PubMed, and the Cochrane Library up to 31 March 2021, randomized clinical trials (RCTs) on probiotics for treating acute diarrhea in children were included. Primary outcomes included the duration of diarrhea and diarrhea lasting ≥2 days, and secondary outcomes included the mean stool frequency on day 2 and duration of hospitalization, fever, and vomiting. We assessed the certainty of the evidence of outcomes according to Grading of Recommendations Assessment, Development, and Evaluation (GRADE) guideline. Eighty-four studies with twenty-one different interventions in 13,443 children were included. For the primary outcomes, moderate evidence indicated that, *Lactobacillus reuteri* [mean difference (MD) = −0.84 day; 95% confidence interval (CI), −1.39, −0.29], *Bifidobacterium lactis* (MD = −0.98 day; 95%CI, −1.82, −0.14), *Saccharomyces boulardii* (MD = −1.25 day; 95%CI, −1.59, −0.91), *Lactobacillus species* (spp.) plus *Bifidobacterium* spp. plus *Saccharomyces* spp. (MD = −1.19 day; 95%CI, −1.81, −0.58), and *Bacillus* spp. plus *Enterococcus* spp. plus *Clostridium* spp. (MD = −1.1 day; 95%CI, −1.84, −0.35) significantly reduced the duration of diarrhea when compared with placebo. *Saccharomyces boulardii* [Odds ratio (OR) = 0.22; 95%CI, 0.11, 0.41] and *Lactobacillus reuteri* (OR = 0.23; 95%CI, 0.090, 0.60) significantly reduced the risk of diarrhea lasting ≥2 days when compared with placebo or no treatment, with moderate evidence. Among all probiotics, *Saccharomyces boulardii* may be the most effective in reducing both duration of diarrhea (compared with placebo) and risk of diarrhea lasting ≥2 days (compared with placebo or no treatment), with moderate evidence. To be conclusive, *Saccharomyces boulardii* may be the most effective probiotic for treating acute diarrhea in children, followed by several other single-strain and multi-strain probiotics.

## 1. Introduction

Diarrhea is common among infants, usually acute, which is mainly caused by infection [1]. In 2017, diarrhea accounted for 533,768 deaths among children under five, mainly in developing countries [2]. World Health Organization defines diarrhea as three or more loose or watery stools within 24 h, and diarrhea is classified as acute if the duration of diarrhea is less than 14 days [3]. Diarrhea could result in dehydration and electrolyte disturbances in children. Significant consequences related to diarrhea in children include growth stunting, malnutrition, and impaired cognitive development [4].

Intestinal microbiota, which is closely associated with human health, has been a research hotspot. The derangement of intestinal microflora is the hallmark of diarrhea [5]. Probiotics are live microorganisms and have been proved to be beneficial for treating diarrhea [6,7]. Probiotics show antidiarrheal activity via several mechanisms, including promoting intestinal microflora balance, enhancing host immunity, and enhancing the gut barrier function [5,8,9,10,11].

Oral rehydration solution (ORS) is the mainstay treatment modality for diarrhea. However, it cannot halt the progression of diarrhea, nor address microflora imbalance, pathogen clearance, and environmental enteric dysfunction [11,12], all of them may be addressed by probiotics. Probiotics were recommended as adjuncts to effectively alleviate and treat diarrhea by clinical practice guidelines and numerous studies [7,13,14,15]. Traditional pairwise meta-analysis only focused on one probiotic and failed to find the most effective probiotic [6,16,17,18]. The most effective probiotic for children with acute diarrhea remains controversial. Besides, the effectiveness of multi-strain probiotics is yet to be evaluated. Superior to traditional pairwise meta-analysis, network meta-analysis could compare the effectiveness of different probiotics in treating acute diarrhea. We conducted a Bayesian network meta-analysis (NMA) of RCTs to assess the effectiveness of a broad spectrum of single-strain and multi-strain probiotics, determining the most effective probiotic in treating acute diarrhea for children.

## 2. Methods

The NMA was performed following the Reporting Items for Systematic Reviews and Meta-Analyses (PRISMA) statement [19,20]. We have registered the protocol on PROSPERO (registration number: CRD42021247282).

### 2.1. Search Strategy

By searching the Cochrane Library, PubMed, and EMBASE, randomized controlled trials of probiotics for treating acute diarrhea in children were included. We limited our search to English. The search date for these databases ended on 31 March 2021. The search terms included: diarrhea, probiotic, children, and randomized controlled trial (Supplementary Material Table S1).

### 2.2. Study Selection and Data Extraction

Studies were included in this NMA if they met the following inclusion criteria: (1) RCT; (2) children (age ≤ 18 years) with acute diarrhea (the frequency of diarrhea was more than three times during a 24-h period and the duration of diarrhea was less than 14 days); (3) children were randomly assigned to receive probiotics, placebo, or no treatment; (4) the study must report at least one of the outcomes (duration of diarrhea, duration of hospitalization, number of patients with diarrhea lasting longer than two days, mean stool frequency on day 2, duration of fever, or duration of vomiting). We excluded the following articles: non–English papers, non-randomized trials, studies with malnourished children, studies without children, studies without probiotics, studies without acute diarrhea, case reports, reviews, meta-analysis studies, conference abstracts, animal studies, in vitro experiments, and letters.

Two investigators independently read the full texts of the included literature and extracted the data. Any disagreements were resolved by discussion. The extracted data included first author, publication, year, country, sample size, probiotic species, and outcomes of interest (duration of diarrhea, duration of hospitalization, number of patients with diarrhea lasting longer than two days, mean stool frequency on day 2, duration of fever, and duration of vomiting).

### 2.3. Quality Assessment

Two investigators independently used Review Manager 5.3 (Cochrane Collaboration) to evaluate the quality of the included studies according to the Cochrane risk of bias assessment tool [21]. In addition, we used the GRADE guideline to assess the certainty of the evidence for each outcome as high, moderate, low, or very low [22,23]. According to the GRADE approach, the certainty of the evidence of RCT was initially considered as high. However, it may be downgraded due to five factors (risk of bias, imprecision, inconsistency, indirectness, and publication bias).

### 2.4. Data Synthesis and Statistical Analysis

The NMA was performed with the “rjags” and “gemtc” packages in R software (version 4.0.1). Network plots were done by STATA (version 14.0). The NMA was conducted with a Bayesian hierarchical model framework [24]. It was applied with four chains of Markov chain Monte Carlo (MCMC) estimation using a random-effect model, running for 100,000 iterations [25]. OR, MD, and their 95% CIs were calculated for the outcomes. When the mean value and standard deviation were not reported, we estimated them based on median, quartile, range, and sample size [26,27,28]. Statistical significance was set at *p*-value < 0.05.

Heterogeneity assessment was performed using the I² index and χ² test. The probability ranking of intervention was achieved by calculating the surface under the cumulative probability ranking curve (SUCRA) [24]. The consistency between direct and indirect comparisons was assessed by a node-splitting method.

## 3. Results

### 3.1. Characteristics of Included Studies

**Figure 1:**
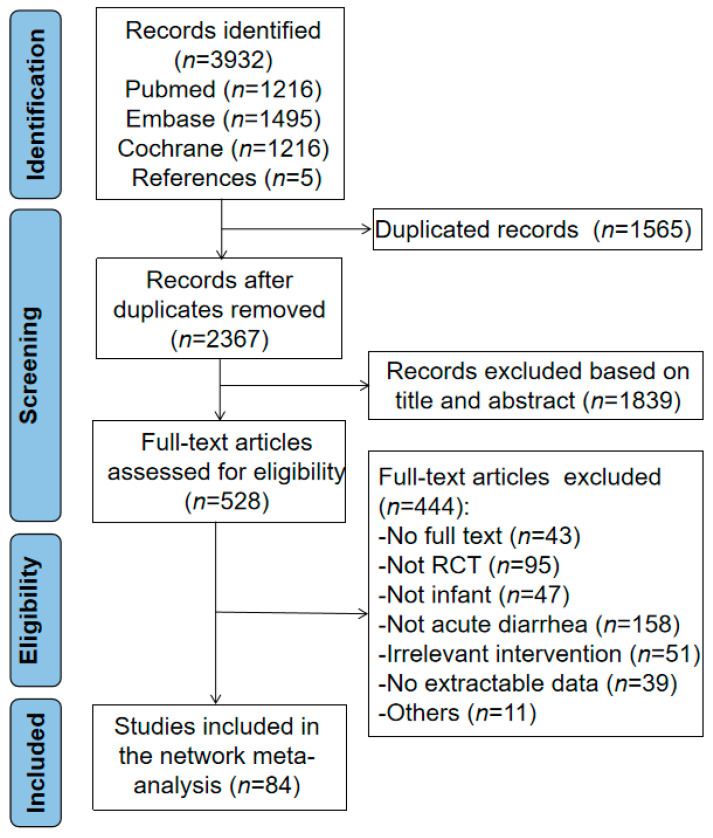
PRISMA flow diagram for the Bayesian network meta-analysis.

A total of 3927 records were retrieved by searching EMBASE, PubMed, and the Cochrane Library. Five studies were found from references. Following the removal of duplicates, 528 potentially eligible articles were identified. Ultimately, eighty-four studies that met the inclusion criteria were included in the NMA. The flow of literature screening is detailed in Figure 1.

**Figure 2:**
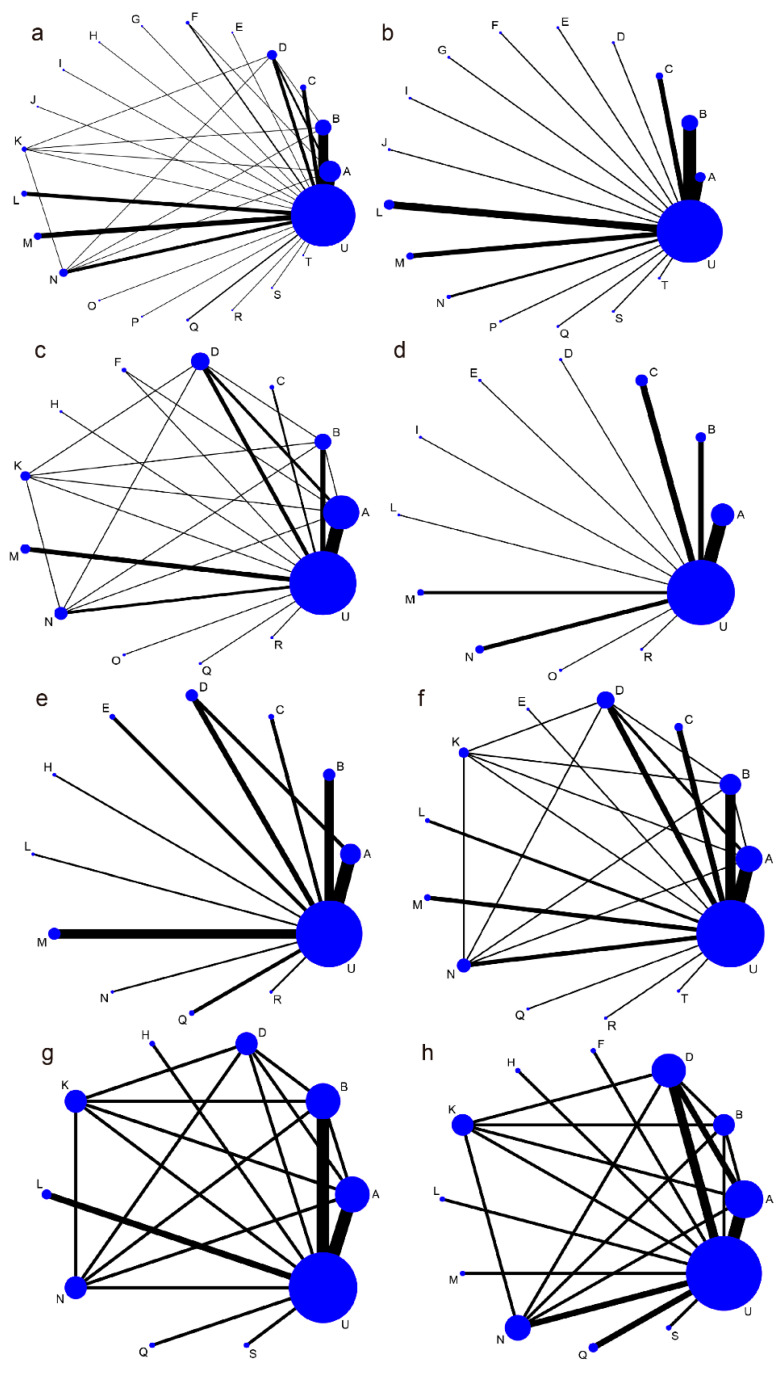
Networks of treatment comparisons. A, *S. boulardii*; B, LGG; C: *L. reuteri*; D, *Bacillus clausii*; E, *L. acidophilus*; F, *B. lactis*; G, *L. sporogenes*; H, *L. plantarum*; I, *ECN 1917*; J, *L. paracasei*; K, *E. faecium*; L, *L.* spp.; M, *L.* spp. + *B.* spp.; N, *L.* spp. + *B.* spp.+ *S.* spp.; O, *L.* spp. + *S.* spp.; P, *B.* spp. + *S.* spp.; Q, *Bacillus* spp. + *E.* spp. + *C.* spp.; R, *L.* spp. + *B.* spp. + *E.* spp.; S, *L.* spp. + *B.* spp. + *P.* spp.; T, *L.* spp. + *S.* spp. + *C.* spp. + *Bacillus* spp.; U, control (placebo/no treatment). (**a**) network of the duration of diarrhea (control = placebo/no treatment); (**b**) network of the duration of diarrhea (control = placebo); (**c**) network of the duration of diarrhea (control = no treatment); (**d**) network for diarrhea lasting ≥ 2 days; (**e**) network of the duration of hospitalization; (**f**) network of the mean stool frequency on day 2; (**g**) network for the duration of vomiting; (**h**) network of the duration of fever. The circle size indicates the number of patients, and the line thickness indicates the number of trials.

Eighty-four studies with twenty-one different interventions involving a total of 13,443 children were included [12,29,30,31,32,33,34,35,36,37,38,39,40,41,42,43,44,45,46,47,48,49,50,51,52,53,54,55,56,57,58,59,60,61,62,63,64,65,66,67,68,69,70,71,72,73,74,75,76,77,78,79,80,81,82,83,84,85,86,87,88,89,90,91,92,93,94,95,96,97,98,99,100,101,102,103,104,105,106,107,108,109,110]. The characteristics and outcomes of included studies are summarized in Supplementary Material Table S2. The relevant literature was published from 1994 to 2020. Of the 84 included studies, 23 were studied in high-income economies, 34 were in lower-middle economies, and 25 were in upper-middle economies, and 2 were multicenter studies. Most of the children were under age five. Figure 2 represents the networks of comparisons for primary outcomes (duration of diarrhea and diarrhea lasting ≥2 days) and secondary outcomes (mean stool frequency on day 2 and duration of hospitalization, fever, and vomiting).

Probiotic interventions could be divided into single-strain and multi-strain probiotics. The single-strain probiotics included *Saccharomyces (S.) boulardii*, *Lactobacillus rhamnosus* GG (LGG), *Lactobacillus (L.) reuteri*, *Bacillus clausii*, *L. acidophilus*, *Bifidobacterium (B.) lactis*, *L. sporogenes*, *L. plantarum*, *Escherichia coli nissle 1917 (ECN 1917)*, *L. paracasei*, and *Enterococcus (E.) faecium*. Multiple-strain probiotics included *L.* species (spp.), *L.* spp. + *B.* spp., *L.* spp. + *B.* spp.+ *S.* spp., *L.* spp. + *S.* spp., *B.* spp. + *S.* spp., *Bacillus* spp. + *E.* spp. + *Clostridium (C.)* spp., *L.* spp. + *B.* spp. + *E.* spp., *L.* spp. + *B.* spp. + *Pediococcus* spp., and *L.* spp. + *S.* spp. + *C.* spp. + *Bacillus* spp. In addition, the control arms included both placebo and no treatment.

### 3.2. Network Meta-Analysis Outcomes

There was no statistical difference or inconsistency for diarrhea lasting ≥2 days, mean stool frequency on day 2, and duration of hospitalization, vomiting, and fever (Supplementary Material Figures S5–S8). In addition, we ranked the interventions for each outcome by calculating the SUCRA (Supplementary Material Table S27). More details about the comparative effectiveness of different probiotics were reported in Supplementary Material Tables S11–S18.

#### 3.2.1. Duration of Diarrhea

We included 73 studies with a total of 12315 children involving 21 interventions (Figure 2a). Compared with the placebo or no treatment, low evidence showed that *B. lactis* (MD = −2.13 days; 95%CI, −3.06, −1.22) had the highest probability of reducing the duration of diarrhea (Table 1 and Supplementary Material Table S27). However, the test suggested significant inconsistency and heterogeneity in the duration of diarrhea (*p* < 0.05, Supplementary Material Figure S3 and Table S3). Therefore, subgroup analysis was performed for the duration of diarrhea based on the control (placebo/no treatment).

Compared with placebo, moderate evidence indicated that *S. boulardii* (MD = −1.25 day; 95%CI, −1.59, −0.91), *L. reuteri* (MD = −0.84 day; 95%CI, −1.39, −0.29), *B. lactis* (MD = −0.98 day; 95%CI, −1.82, −0.14), *L.* spp. + *B.* spp. + *S.* spp. (MD = −1.19 day; 95%CI, −1.81 to −0.58), and *Bacillus* spp. + *E.* spp. + *C.* spp. (MD = −1.1 day; 95%CI, −1.84, −0.35) significantly reduced the duration of diarrhea. In addition, very low or low evidence showed that *ECN 1917* (MD = −1.44 day; 95%CI, −2.16, −0.72), *L.* spp. + *B.* spp. (MD = −0.79 day; 95%CI, −1.22, −0.38), and *L.* spp. + *Bacillus* spp. + *S.* spp. + *C.* spp. (MD = −1.1 day; 95%CI, −2.03, −0.17) could decrease the duration of diarrhea when compared to placebo (Table 1). Additionally, compared with placebo, *S. boulardii* may be the most effective in reducing the duration of diarrhea with moderate evidence (Supplementary Material Table S27).

Compared with no treatment, low or very low evidence represented that the following probiotics could shorten the duration of diarrhea: *S. boulardii* (MD = −0.95 day; 95%CI, −1.33, −0.58), LGG (MD = −1.57 day; 95%CI, −2.13, −1.01), *L. reuteri* (MD = −0.98 day; 95%CI, −1.92, −0.04), *B. lactis* (MD = −3.17 days, 95%CI, −4.47, −1.87), *L.* spp. + *B.* spp. (MD = −0.97 day; 95%CI, −1.57, −0.37), *L.* spp. + *B.* spp. + *S.* spp. (MD = −1.26 day; 95%CI, −1.99, −0.51), and *L.* spp. + *B.* spp. + *E.* spp. (MD = −1.53 day; 95%CI, −2.85, −0.21) (Table 1). *B. lactis* had the highest probability of reducing the duration of diarrhea when compared with no treatment (Supplementary Material Table S27).

#### 3.2.2. Diarrhea Lasting ≥2 Days

The number of children with diarrhea lasting more than two days was reported in 36 studies with 6536 children (Figure 2d). Compared with the controls, the risk of diarrhea lasting ≥2 days was reduced in patients receiving *S. boulardii* (OR = 0.22; 95%CI, 0.11, 0.41) and *L. reuteri* (OR = 0.23; 95%CI, 0.090, 0.60) with moderate evidence. Low evidence indicated that *L.* spp. + *B.* spp. (OR = 0.20; 95%CI, 0.052, 0.77) and *L.* spp. + *B.* spp. + *S.* spp. (OR = 0.35; 95%CI, 0.11, 1.0) contributed to the significant effect (Table 1). Comparing across the probiotics, *S. boulardii* may be the most effective in reducing the risk of diarrhea lasting ≥2 days with moderate evidence (Supplementary Material Table S27).

#### 3.2.3. Duration of Hospitalization

A total of 28 RCTs with 4859 children involving 13 interventions were included (Figure 2e). Compared with the controls, *S. boulardii* (MD = −0.88 day; 95%CI, −1.58, −0.18) and LGG (MD = −1.21 day; 95%CI, −2.09, −0.33) significantly decreased the duration of hospitalization with low evidence (Table 1). In addition, LGG had the highest probability of reducing the duration of hospitalization (Supplementary Material Table S27).

#### 3.2.4. Mean Stool Frequency on Day 2

The mean stool frequency on day 2 was presented in 32 studies with 6471 children involving 13 interventions (Figure 2f). Compared to the controls, moderate evidence indicated that *S. boulardii* (MD = −0.66; 95%CI, −1.1, −0.23), LGG (MD = −0.66; 95%CI, −1.2, −0.14), *L. reuteri* (MD = −1.5; 95%CI, −2.3, −0.61), *L.* spp. + *B.* spp. + *S.* spp. (MD = −0.77; 95%CI, −1.5, −0.014), and *Bacillus* spp. + *E.* spp. + *C.* spp. (MD = −1.6; 95%CI, −2.9, −0.44) could decrease the mean stool frequency on day 2. *L.* spp. + *B.* spp. (MD = −0.78; 95%CI, −1.6, −0.021; very low evidence) showed the significant effect (Table 1). In addition, *Bacillus* spp. + *E.* spp. + *C.* spp. may be the most effective in reducing the mean stool frequency on day 2 with moderate evidence (Supplementary Material Table S27).

#### 3.2.5. Duration of Vomiting and Fever

We included 12 studies with a total of 2385 children involving 11 interventions (Figure 2g). Only *Bacillus* spp. + *E.* spp. + *C.* spp. (MD = −0.63 day; 95%CI, −1.15, −0.11; moderate evidence) could reduce the duration of vomiting when compared to the controls (Table 1).

The duration of fever was reported in 13 studies with 1411 children involving 12 interventions (Figure 2h). No treatment could shorten the duration of fever (Table 1).

### 3.3. Risk of Bias Assessment

We conducted a quality assessment of the included studies according to the Cochrane handbook 5.3. Among the 84 studies, random sequence generation was rated as “low risk” in 48 studies (57%); 40 studies (48%) were judged at “low risk” for allocation concealment; 39 (46%) were classified as “low risk” for blinding of children’s parents and personnel; 31 (37%) were classified as “low risk” for blinding of outcome assessment; 62 (74%) represented “low risk” for incomplete outcome data (Supplementary Material Figures S1 and S2).

## 4. Discussion

Diarrhea not only seriously endangers the long-term physical development health of children but also poses a substantial socioeconomic burden [4,111]. In the NMA, we included eighty-four randomized controlled trials with a total of 13,443 children involving twenty-one interventions to illustrate the clinical effect of probiotics for treating acute diarrhea. We found that certain single-strain and multi-strain probiotics effectively treated acute diarrhea. For single-strain probiotics, moderate evidence showed that *L. reuteri* and *S. boulardii* could reduce the duration of diarrhea, the risk of diarrhea lasting ≥2 days, and the mean stool frequency on day 2. Evidence with a low or very low certainty suggested that *B. lactis*, LGG, and *ECN 1917* could shorten the duration of diarrhea. For multi-strain probiotics, *L.* spp. + *B.* spp. + *S.* spp. could decrease the duration of diarrhea and the mean stool frequency on day 2 with moderate evidence. Low evidence indicated that *L.* spp. + *B.* spp., *Bacillus* spp. + *E.* spp. + *C.* spp., *L.* spp. + *B.* spp. + *E.* spp., and *L.* spp. + *Bacillus* spp. + *S.* spp. + *C.* spp. could decrease the duration of diarrhea. Only *Bacillus* spp. + *E.* spp. + *C.* spp. could reduce the duration of vomiting with moderate evidence. Remarkably, for the primary outcomes, *S. boulardii* may be the most effective in reducing the duration of diarrhea (compared with placebo) and the risk of diarrhea lasting ≥2 days (compared with placebo or no treatment) with moderate evidence.

Our study demonstrated that probiotics could facilitate antidiarrheal effects on acute diarrhea in children, which was consistent with a previous report [7]. Previous pairwise meta-analysis only elucidated the effectiveness of single-strain probiotics [6,7,16,17,112], but multi-strain probiotics were not analyzed, nor did they assess the comparative effectiveness of different probiotics. The effectiveness of probiotics varied greatly, and which probiotic was the most effective for treating acute diarrhea remained controversial. Our NMA has some advantages. The NMA is comprehensive, including all common probiotics. Besides, we ranked the effectiveness of probiotic treatments by calculating the surface under the cumulative probability ranking curve (SUCRA, Supplementary Material Table S27) and evaluated the comparative effectiveness of probiotics (Supplementary Material Tables S11–S18). Notably, we used the GRADE methodology to assess the certainty of the evidence (Supplementary Material Tables S19–S26), which could provide a highly reliable reference for clinical practice.

For the duration of diarrhea, we identified significant heterogeneity and inconsistency in the two comparisons (*S. boulardii* vs LGG and *S. boulardii* vs *B. lactis*; Supplementary Material Figure S3 and Table S3). Therefore, for the two comparisons, we used the results of direct comparison instead of NMA. In addition, we performed a subgroup analysis based on the control (placebo/no treatment). Compared with placebo, *S. boulardii* (moderate evidence), *L. reuteri* (moderate evidence), *B. lactis* (low evidence), and *ECN 1917* (very low evidence) could shorten the duration of diarrhea. Interestingly, LGG significantly reduced the duration of diarrhea compared with no treatment, but such the effect was not observed compared with placebo. It was consistent with the previous study by Szajewska et al. [16]. It may be because most of the valid trials of LGG were open-label, which resulted in exaggerating the effectiveness. Placebo-controlled trials provide more conservative effectiveness estimates with high credibility. Effectiveness evaluation of probiotics needs to be assessed based on the results of placebo-controlled trials. Results of LGG should be considered with caution, and further investigations are needed to validate its effectiveness.

In addition to reducing the duration of diarrhea, *S. boulardii* may also reduce the duration of hospitalization (low evidence), the risk of diarrhea lasting ≥2 days (moderate evidence), and the mean stool frequency on day 2 (moderate evidence). Therefore, we recommend *S. boulardii* as the best probiotic for the treatment of acute diarrhea in children. However, *S. boulardii* was present in some human stools but was not found in most children. It has been reported in cases of fungal sepsis [113]. More studies are needed to elaborate on the detailed mechanisms of *S. boulardii*’s antidiarrheal effect. Low evidence indicated that *B. lactis* ranked the first in reducing the duration of diarrhea when compared with placebo or no treatment. Several studies suggested that *B. lactis* could improve gut barrier function, enhance immunity against pathogens, and regulate intestinal flora, thereby facilitating overall health recovery [114,115]. However, compared with placebo, *S. boulardii* may be more effective than *B. lactis* in reducing the duration of diarrhea. Therefore, we recommend, but inferior to *S. boulardii*, that *B. lactis* treats acute diarrhea in children. More randomized controlled trials are required to verify the effectiveness of *B. lactis*.

In pediatrics, several studies have found that multi-strain probiotics were effective, even better than single-strain probiotics [14,116,117,118]. *L.* spp. + *B.* spp. + *S.* spp. possibly decreased the duration of diarrhea (moderate evidence), the risk of diarrhea lasting ≥2 days (low evidence), and the mean stool frequency on day 2 (moderate evidence). It suggested that the combination of *Lactobacillus* spp., *Bifidobacterium* spp., and *Saccharomyces* spp. produced a significant effect. Together with the above results of the NMA, we recommend a mixture of LGG, *L. reuteri*, *B. lactis*, and *S. boulardii* to treat acute diarrhea in children. Remarkably, the success of probiotic treatments may be reduced in well-nourished, rotavirus-vaccinated children, resulting in a signal-to-noise problem. Two large RCTs involving almost 1000 children reflected it [37,40].

Limitations to the NMA need to be acknowledged. First, several studies did not provide the mean and standard deviation. We performed the data estimation of them based on median, quartile, range, and sample size, so that the authenticity of some data was not high. Second, malnutrition and HIV-infected children were not included in the NMA. However, diarrhea contributed to a significant share of morbidity and mortality in these children in the sub–Sahara African region [1,2]. Third, the longer-term outcomes of diarrhea (growth stunting, malnutrition, and impaired cognitive development) were not evaluated because few studies provided them. Fourth, of the included studies, only one was conducted in sub-Saharan Africa where there was the greatest burden of diarrhea-associated mortality and stunting [1,2]. Fifth, most of the included studies were assessed as moderate or high risk using the Cochrane risk of bias assessment tool. We used the GRADE guideline to assess the certainty of the evidence. Results need to be carefully considered based on the level of evidence. Sixth, there was considerable heterogeneity of results in the direct comparison meta-analysis. Finally, we did not analyze different doses of probiotics because the data was unavailable in some studies. Various doses of probiotics may exert different effects.

In the NMA, we illustrated the clinical effect of single-strain and multi-strain probiotics and determined the comparative effectiveness of various probiotics. To our knowledge, it is the first network meta-analysis that systematically evaluates the effectiveness of probiotics for treating acute diarrhea in children. Our results showed that probiotics could reduce the duration of diarrhea in children by 1–2 days. Probiotics may be cost-effective for treating acute diarrhea in children because probiotics are cheap. The results of this study may provide a valuable reference for decision-making in a clinical setting.

## 5. Conclusions

In the Bayesian network meta-analysis of 84 studies involving 13,443 children, we found that certain single-strain (including *Saccharomyces boulardii*, LGG, *Lactobacillus reuteri*, *Bifidobacterium lactis*, and *ECN 1917*) and multi-strain probiotics effectively treated acute diarrhea in children with various certainty evidence. *Saccharomyces boulardii* may be the most effective probiotic for treating acute diarrhea in children. Besides, *Bifidobacterium lactis* was a promising probiotic. More studies are needed to verify the results.

## Figures and Tables

**Table 1:** Results of comparisons of interventions for each outcome in the network meta-analysis.

Intervention	Duration of Diarrhea (MD, 95%CI)	Duration of Diarrhea (Control = Placebo) (MD, 95%CI)	Duration of Diarrhea (Control = No Treatment) (MD, 95%CI)	Diarrhea Lasting ≥ 2 Days (OR, 95%CI)	Duration of Hospitalization (MD, 95%CI)	Mean stool Frequency on Day 2 (MD, 95%CI)	Duration of Vomiting (MD, 95%CI)	Duration of Fever (MD, 95%CI)
*S. boulardii*	**−0.98** ^c^ **(−1.29, −0.68)**	**−1.25** ^b^ **(−1.59, −0.91)**	**−0.95** ^c^ **(−1.33, −0.58)**	**0.22** ^b^ **(0.11, 0.41)**	**−0.88** ^c^ **(−1.58, −0.18)**	**−0.66** ^b^ **(−1.1, −0.23)**	−0.07 ^b^ (−0.31, 0.19)	−0.18 ^c^ (−0.53, 0.16)
LGG	**−0.78** ^c^ **(−1.12, −0.44)**	−0.23 ^b^ (−0.51, 0.02)	**−1.57** ^c^ **(−2.13, −1.01)**	0.56 ^c^ (0.21, 1.4)	**−1.21** ^c^ **(−2.09, −0.33)**	**−0.66** ^b^ **(−1.2, −0.14)**	−0.16 ^a^ (−0.42, 0.09)	−0.56 ^b^ (−1.2, 0.11)
*L. reuteri*	**−0.91** ^b^ **(−1.5, −0.32)**	**−0.84** ^b^ **(−1.39, −0.29)**	**−0.98** ^c^ **(−1.92, −0.04)**	**0.23** ^b^ **(0.090, 0.60)**	−0.69 ^c^ (−2.02, 0.65)	**−1.5** ^b^ **(−2.3, −0.61)**	-	-
*Bacillus clausii*	−0.26 ^c^ (−0.78, 0.26)	−0.26 ^b^ (−0.96, 0.44)	−0.31 ^c^ (−0.90, 0.28)	0.32 ^c^ (0.020, 4.2)	−0.35 ^c^ (−1.36, 0.65)	−0.40 ^a^ (−0.98, 0.18)	−0.12 ^b^ (−0.61, 0.38)	−0.1 ^b^ (−0.5, 0.33)
*L. acidophilus*	0.03 ^c^ (−1.27, 1.32)	0.03 ^b^ (−0.74, 0.8)	-	0.84 ^c^ (0.11, 6.2)	−0.28 ^c^ (−1.6, 1.04)	0.0018 ^c^ (−1.9, 1.9)	-	-
*B. lactis*	**−2.13** ^c^ **(−3.06, −1.22)**	**−0.98** ^b^ **(−1.82, −0.14)**	**−3.17** ^c^ **(−4.47, −1.87)**	-	-	-		−0.52 ^c^ (−1.3, 0.27)
*L. sporogenes*	−0.1 ^b^ (−1.36, 1.17)	−0.1 ^b^ (−0.82, 0.62)	-	-	-	-	-	-
*L. plantarum*	−1.23 ^c^ (−2.52, 0.06)	-	−1.23 ^c^ (−2.55, 0.08)	-	0.23 ^c^ (−1.69, 2.15)	-	−0.08 ^c^ (−0.84, 0.68)	−0.23 ^c^ (−2.13, 1.64)
*ECN 1917*	**−1.44** ^d^ **(−2.70, −0.17)**	**−1.44** ^d^ **(−2.16, −0.72)**	-	0.50 ^d^ (0.062, 4.0)	-	-	-	-
*L. paracasei*	−0.16 ^b^ (−1.48, 1.16)	−0.16 ^b^ (−0.98, 0.65)	-	-	-	-	-	-
*E. faecium*	0.16 ^b^ (−0.88, 1.20)	-	−0.01 ^b^ (−1.09, 1.06)	-	-	−0.22 ^b^ (−1.3, 0.80)	−0.12 ^b^ (−0.6, 0.36)	0.08 ^b^ (−0.68, 0.86)
*L.* spp.	−0.31 ^a^ (−0.93, 0.31)	−0.23 ^a^ (−0.67, 0.18)	-	1.0 ^b^ (0.14, 7.3)	−1.1 ^c^ (−3.06, 0.86)	−0.22 ^c^ (−1.1, 0.70)	−0.24 ^b^ (−0.79, 0.31)	−0.2 ^c^ (−1.29, 0.89)
*L.* spp. + *B.* spp.	**−0.86** ^c^ **(−1.33, −0.4)**	**−0.79** ^c^ **(−1.22, −0.38)**	**−0.97** ^c^ **(−1.57, −0.37)**	**0.20** ^c^ **(0.052, 0.77)**	−0.65 ^c^ (−1.5, 0.19)	**−0.78** ^d^ **(−1.6, −0.021)**	-	−0.66 ^c^ (−1.7, 0.39)
*L.* spp. + *B.* spp. + *S.* spp.	**−1.17** ^b^ **(−1.75, −0.59)**	**−1.19** ^b^ **(−1.81, −0.58)**	**−1.26** ^d^ **(−1.99, −0.51)**	**0.35** ^c^ **(0.11, 1.0)**	−0.74 ^c^ (−2.69, 1.22)	**−0.77** ^b^ **(−1.5, −0.014)**	−0.26 ^b^ (−0.75, 0.23)	−0.12 ^b^ (−0.71, 0.47)
*L.* spp. + *S.* spp.	−0.73 ^c^ (−2.07, 0.61)	-	−0.73 ^c^ (−2.1, 0.64)	0.26 ^d^ (0.031, 2.2)	-	-	-	-
*B.* spp. + *S.* spp.	−0.04 ^c^ (−1.28, 1.20)	−0.04 ^c^ (−0.71, 0.63)	-	-	-	-	-	-
*Bacillus* spp. + *E.* spp. + *C.* spp.	**−1.1** ^c^ **(−2.02, −0.18)**	**−1.1** ^b^ **(−1.84, −0.35)**	−1.1 ^c^ (−2.44, 0.24)	-	−0.46 ^c^ (−1.8, 0.91)	**−1.6** ^b^ **(−2.9, −0.44)**	**−0.63** ^b^ **(−1.15, −0.11)**	−0.12 ^b^ (−0.65, 0.44)
*L.* spp. + *B.* spp. + *E.* spp.	**−1.53** ^c^ **(−2.82, −0.24)**	-	**−1.53** ^c^ **(−2.85, −0.21)**	0.16 ^d^ (0.019, 1.4)	−0.83 ^c^ (−2.73, 1.07)	−1.3 ^c^ (−2.6, 0.073)	-	-
*L.* spp. + *B.* spp. + *Pediococcus* spp.	−1.1 ^c^ (−2.66, 0.47)	−1.1 ^c^ (−2.27, 0.08)	-	-	-	-	−1.20 ^c^ (−2.51, 0.11)	−0.5 ^c^ (−1.97, 0.97)
*L.* spp. + *Bacillus* spp. + *S.* spp. + *C.* spp.	−1.1 ^c^ (−2.49, 0.29)	**−1.1** ^c^ **(−2.03, −0.17)**	-	-	-	−0.85 ^c^ (−2.5, 0.77)	-	-

MD, mean difference; OR, odds ratio; CI, confidence interval. ^a^ high certainty evidence. ^b^ moderate certainty evidence. ^c^ low certainty evidence. ^d^ very low certainty evidence. It would be considered statistically significant (*p* < 0.05) if the 95% CI of OR did not contain 1 or the 95% CI of MD did not contain 0.

## Data Availability

Not applicable.

## Supplementary Materials

The following are available online at https://www.mdpi.com/2072-6643/13/12/4319/s1. Figure S1: Risk of bias graph; Figure S2: Risk of bias summary; Figure S3: Incoherence plot for the duration of diarrhea (control = placebo/no treatment); Figure S4: Incoherence plot for the duration of diarrhea (control = no treatment); Figure S5: Incoherence plot for the duration of hospitalization; Figure S6: Incoherence plot for the mean stool frequency on day 2; Figure S7: Incoherence plot for the duration of vomiting; Figure S8: Incoherence plot for the duration of fever; Table S1: Search strategies; Table S2: Characteristics of included studies; Table S3: Heterogeneity for the duration of diarrhea (control = placebo/no treatment); Table S4; Heterogeneity for the duration of diarrhea (control = placebo); Table S5: Heterogeneity for the duration of diarrhea (control = no treatment); Table S6: Heterogeneity for diarrhea lasting ≥2 days; Table S7: Heterogeneity for the duration of hospitalization; Table S8: Heterogeneity for the mean stool frequency on day 2; Table S9: Heterogeneity for the duration of vomiting; Table S10: Heterogeneity for the duration of fever; Table S11: NMA results for the duration of diarrhea (control = placebo/no treatment); Table S12: NMA results for the duration of diarrhea (control = placebo); Table S13: NMA results for the duration of diarrhea (control = no treatment); Table S14: NMA results for diarrhea lasting ≥2 days; Table S15: NMA results for the duration of hospitalization; Table S16: NMA results for the mean stool frequency on day 2; Table S17: NMA results for the duration of vomiting; Table S18: NMA results for the duration of fever; Table S19: Certainty of evidence for the duration of diarrhea; Table S20: Certainty of evidence for the duration of diarrhea (control = placebo); Table S21: Certainty of evidence for the duration of diarrhea (control = no treatment); Table S22: Certainty of evidence for diarrhea lasting ≥2 days; Table S23: Certainty of evidence for the duration of hospitalization; Table S24: Certainty of evidence for the mean stool frequency on day 2; Table S25: Certainty of evidence for the duration of vomiting; Table S26: Certainty of evidence for the duration of fever; Table S27: Rank for outcomes.

## References

[1] Troeger C., Colombara D.V., Rao P.C., Khalil I.A., Brown A., Brewer T.G., Guerrant R.L., Houpt E.R., Kotloff K.L., Misra K. (2018). Global disability-adjusted life-year estimates of long-term health burden and undernutrition attributable to diarrhoeal diseases in children younger than 5 years. Lancet Glob. Health.

[2] GBD 2017 Diarrhoeal Disease Collaborators (2020). Quantifying risks and interventions that have affected the burden of diarrhoea among children younger than 5 years: An analysis of the Global Burden of Disease Study 2017. Lancet Infect. Dis..

[3] World Health Organization (1988). Persistent diarrhoea in children in developing countries: Memorandum from a WHO meeting. Bull. World Health Organ..

[4] Guerrant R.L., DeBoer M.D., Moore S.R., Scharf R.J., Lima A.A. (2013). The impoverished gut—A triple burden of diarrhoea, stunting and chronic disease. Nat. Rev. Gastroenterol. Hepatol..

[5] Li Y., Xia S., Jiang X., Feng C., Gong S., Ma J., Fang Z., Yin J., Yin Y. (2021). Gut Microbiota and Diarrhea: An Updated Review. Front. Cell. Infect. Microbiol..

[6] Szajewska H., Kołodziej M., Zalewski B.M. (2020). Systematic review with meta-analysis: *Saccharomyces boulardii* for treating acute gastroenteritis in children—A 2020 update. Aliment. Pharmacol. Ther..

[7] Collinson S., Deans A., Padua-Zamora A., Gregorio G.V., Li C., Dans L.F., Allen S.J. (2020). Probiotics for treating acute infectious diarrhoea. Cochrane Database Syst. Rev..

[8] Suez J., Zmora N., Segal E., Elinav E. (2019). The pros, cons, and many unknowns of probiotics. Nat. Med..

[9] Thomas C.M., Versalovic J. (2010). Probiotics-host communication: Modulation of signaling pathways in the intestine. Gut Microbes.

[10] Wieers G., Belkhir L., Enaud R., Leclercq S., Philippart de Foy J.M., Dequenne I., de Timary P., Cani P.D. (2019). How Probiotics Affect the Microbiota. Front. Cell. Infect. Microbiol..

[11] Binder H.J., Brown I., Ramakrishna B.S., Young G.P. (2014). Oral rehydration therapy in the second decade of the twenty-first century. Curr. Gastroenterol. Rep..

[12] Islam T.M.D.T., Hussain T., Rahman A., Quaium S.M.M.A., Hamid F. (2019). Clinical Efficacy of *Bacillus clausii* Probiotic in the Management of Acute Diarrhoea in Children. Chattagram Maa-O-Shishu Hosp. Med. Coll. J..

[13] Depoorter L., Vandenplas Y. (2021). Probiotics in Pediatrics. A Review and Practical Guide. Nutrients.

[14] Tremblay A., Xu X., Colee J., Tompkins T.A. (2021). Efficacy of a Multi-Strain Probiotic Formulation in Pediatric Populations: A Comprehensive Review of Clinical Studies. Nutrients.

[15] Malagón-Rojas J.N., Mantziari A., Salminen S., Szajewska H. (2020). Postbiotics for Preventing and Treating Common Infectious Diseases in Children: A Systematic Review. Nutrients.

[16] Szajewska H., Kołodziej M., Gieruszczak-Białek D., Skórka A., Ruszczyński M., Shamir R. (2019). Systematic review with meta-analysis: *Lactobacillus rhamnosus* GG for treating acute gastroenteritis in children—A 2019 update. Aliment. Pharmacol. Ther..

[17] Ianiro G., Rizzatti G., Plomer M., Lopetuso L., Scaldaferri F., Franceschi F., Cammarota G., Gasbarrini A. (2018). *Bacillus clausii* for the Treatment of Acute Diarrhea in Children: A Systematic Review and Meta-Analysis of Randomized Controlled Trials. Nutrients.

[18] Szajewska H., Guarino A., Hojsak I., Indrio F., Kolacek S., Orel R., Salvatore S., Shamir R., van Goudoever J.B., Vandenplas Y. (2020). Use of Probiotics for the Management of Acute Gastroenteritis in Children: An Update. J. Pediatr. Gastroenterol. Nutr..

[19] Moher D., Liberati A., Tetzlaff J., Altman D.G., Group P. (2009). Preferred reporting items for systematic reviews and meta-analyses: The PRISMA statement. BMJ.

[20] Hutton B., Salanti G., Caldwell D.M., Chaimani A., Schmid C.H., Cameron C., Ioannidis J.P., Straus S., Thorlund K., Jansen J.P. (2015). The PRISMA extension statement for reporting of systematic reviews incorporating network meta-analyses of health care interventions: Checklist and explanations. Ann. Intern. Med..

[21] Higgins J.P., Altman D.G., Gotzsche P.C., Juni P., Moher D., Oxman A.D., Savovic J., Schulz K.F., Weeks L., Sterne J.A. (2011). The Cochrane Collaboration’s tool for assessing risk of bias in randomised trials. BMJ.

[22] Puhan M.A., Schunemann H.J., Murad M.H., Li T., Brignardello-Petersen R., Singh J.A., Kessels A.G., Guyatt G.H., Group G.W. (2014). A GRADE Working Group approach for rating the quality of treatment effect estimates from network meta-analysis. BMJ.

[23] Brignardello-Petersen R., Bonner A., Alexander P.E., Siemieniuk R.A., Furukawa T.A., Rochwerg B., Hazlewood G.S., Alhazzani W., Mustafa R.A., Murad M.H. (2018). Advances in the GRADE approach to rate the certainty in estimates from a network meta-analysis. J. Clin. Epidemiol..

[24] Salanti G., Ades A.E., Ioannidis J.P. (2011). Graphical methods and numerical summaries for presenting results from multiple-treatment meta-analysis: An overview and tutorial. J. Clin. Epidemiol..

[25] Shim S.R., Kim S.J., Lee J., Rucker G. (2019). Network meta-analysis: Application and practice using R software. Epidemiol. Health.

[26] Wan X., Wang W., Liu J., Tong T. (2014). Estimating the sample mean and standard deviation from the sample size, median, range and/or interquartile range. BMC Med. Res. Methodol..

[27] Luo D., Wan X., Liu J., Tong T. (2018). Optimally estimating the sample mean from the sample size, median, mid-range, and/or mid-quartile range. Stat. Methods Med. Res..

[28] Shi J., Luo D., Weng H., Zeng X.T., Lin L., Chu H., Tong T. (2020). Optimally estimating the sample standard deviation from the five-number summary. Res. Synth. Methods.

[29] Vaghela P., Langade R.A. (2020). Analysis of impact of ors with zinc & probiotics supplements in curing acute diarrhoea. Int. J. Res. Pharm. Sci..

[30] Shin D.Y., Yi D.Y., Jo S., Lee Y.M., Kim J.H., Kim W., Park M.R., Yoon S.M., Kim Y., Yang S. (2020). Effect of a new *Lactobacillus plantarum* product, LRCC5310, on clinical symptoms and virus reduction in children with rotaviral enteritis. Medicine.

[31] Mourey F., Sureja V., Kheni D., Shah P., Parikh D., Upadhyay U., Satia M., Shah D., Troise C., Decherf A. (2020). A Multicenter, Randomized, Double-Blind, Placebo-Controlled Trial of *Saccharomyces boulardii* in Infants and Children with Acute Diarrhea. Pediatr. Infect. Dis. J..

[32] Kluijfhout S., Trieu T.V., Vandenplas Y. (2020). Efficacy of the Probiotic Probiotical Confirmed in Acute Gastroenteritis. Pediatr. Gastroenterol. Hepatol. Nutr..

[33] Chen K., Xin J., Zhang G., Xie H., Luo L., Yuan S., Bu Y., Yang X., Ge Y., Liu C. (2020). A combination of three probiotic strains for treatment of acute diarrhoea in hospitalised children: An open label, randomised controlled trial. Benef. Microbes.

[34] Szymański H., Szajewska H. (2019). Lack of Efficacy of Lactobacillus reuteri DSM 17938 for the Treatment of Acute Gastroenteritis: A Randomized Controlled Trial. Pediatr. Infect. Dis. J..

[35] Sudha M.R., Jayanthi N., Pandey D.C., Verma A.K. (2019). *Bacillus clausii* UBBC-07 reduces severity of diarrhoea in children under 5 years of age: A double blind placebo controlled study. Benef. Microbes.

[36] Vidjeadevan D., Vinoth S., Ramesh S. (2018). Role of *Saccharomyces boulardii* and *Bacillus clausii* in reducing the duration of diarrhea: A three-armed randomised controlled trial. Int. J. Contemp. Pediatr..

[37] Schnadower D., Tarr P.I., Casper T.C., Gorelick M.H., Dean J.M., O’Connell K.J., Mahajan P., Levine A.C., Bhatt S.R., Roskind C.G. (2018). *Lactobacillus rhamnosus* GG versus Placebo for Acute Gastroenteritis in Children. N. Engl. J. Med..

[38] Javeed A., Manzoor S., Wamiq S. (2018). Effect of oral *Saccharomyces boulardii* supplementation on the duration of acute watery diarrhea in children. Pak. J. Med. Health Sci..

[39] Hong Chau T.T., Minh Chau N.N., Hoang Le N.T., Chung The H., Voong Vinh P., Nguyen To N.T., Ngoc N.M., Tuan H.M., Chau Ngoc T.L., Kolader M.E. (2018). A Double-blind, Randomized, Placebo-controlled Trial of *Lactobacillus acidophilus* for the Treatment of Acute Watery Diarrhea in Vietnamese Children. Pediatr. Infect. Dis. J..

[40] Freedman S.B., Williamson-Urquhart S., Farion K.J., Gouin S., Willan A.R., Poonai N., Hurley K., Sherman P.M., Finkelstein Y., Lee B.E. (2018). Multicenter Trial of a Combination Probiotic for Children with Gastroenteritis. N. Engl. J. Med..

[41] Bhat S., Shreekrishna G.N., Savio C.D. (2018). Efficacy of probiotics in acute diarrhoea in children. Int. J. Contemp. Pediatr..

[42] Sirsat G.M., Sankpal D.M. (2017). Role of *Saccharomyces boulardii* in management of acute diarrhoea of children—A randomized controlled trial. MedPulse Int. J. Pediatr..

[43] Park M.S., Kwon B., Ku S., Ji G.E. (2017). The Efficacy of Bifidobacterium longum BORI and *Lactobacillus acidophilus* AD031 Probiotic Treatment in Infants with Rotavirus Infection. Nutrients.

[44] Burki M.F.K., Jabeen F. (2017). Efficacy of *Saccharomyces boullardii* in children with acute diarrhea. Med. Forum Mon..

[45] Yazar A.S., Güven Ş., Dinleyici E. (2016). Effects of zinc or synbiotic on the duration of diarrhea in children with acute infectious diarrhea. Turk. J. Gastroenterol..

[46] Sharif M.R., Kashani H.H., Ardakani A.T., Kheirkhah D., Tabatabaei F., Sharif A. (2016). The Effect of a Yeast Probiotic on Acute Diarrhea in Children. Probiotics Antimicrob. Proteins.

[47] García-Menor E., García-Marín F., Vecino-López R., Horcajo-Martínez G., de Ibarrondo Guerrica-Echevarría M.J., Gómez-González P., Velasco-Ortega S., Suárez-Almarza J., Nieto-Magro C. (2016). A Multicenter, Prospective, Randomized Controlled Trial to Evaluate the Additional Benefit of a Multistrain Synbiotic (Prodefen^®^) in the Clinical Management of Acute Viral Diarrhea in Children. Glob. Pediatr. Health.

[48] Dash D.K., Dash M., Mohanty M.D., Acharya N. (2016). Efficacy of probiotic *Saccharomyces boulardii* as an adjuvant therapy in acute childhood diarrhoea. J. Nepal Paediatr. Soc..

[49] Das S., Gupta P.K., Das R.R. (2016). Efficacy and Safety of *Saccharomyces boulardii* in Acute Rotavirus Diarrhea: Double Blind Randomized Controlled Trial from a Developing Country. J. Trop. Pediatr..

[50] Lee D.K., Park J.E., Kim M.J., Seo J.G., Lee J.H., Ha N.J. (2015). Probiotic bacteria, *B. longum* and *L. acidophilus* inhibit infection by rotavirus in vitro and decrease the duration of diarrhea in pediatric patients. Clin. Res. Hepatol. Gastroenterol..

[51] Hegar B., Waspada I.M., Gunardi H., Vandenplas Y. (2015). A double blind randomized trial showing probiotics to be ineffective in acute diarrhea in Indonesian children. Indian J. Pediatr..

[52] Freedman S.B., Sherman P.M., Willan A., Johnson D., Gouin S., Schuh S. (2015). Emergency Department Treatment of Children with Diarrhea who Attend Day Care: A Randomized Multidose Trial of a *Lactobacillus helveticus* and *Lactobacillus rhamnosus* Combination Probiotic. Clin. Pediatr..

[53] El-Soud N.H.A., Said R.N., Mosallam D.S., Barakat N.A.M., Sabry M.A. (2015). *Bifidobacterium lactis* in treatment of children with acute diarrhea. A randomized double blind controlled trial. Maced. J. Med. Sci..

[54] Dinleyici E.C., Kara A., Dalgic N., Kurugol Z., Arica V., Metin O., Temur E., Turel O., Guven S., Yasa O. (2015). *Saccharomyces boulardii* CNCM I-745 reduces the duration of diarrhoea, length of emergency care and hospital stay in children with acute diarrhoea. Benef. Microbes.

[55] Dinleyici E.C., Dalgic N., Guven S., Metin O., Yasa O., Kurugol Z., Turel O., Tanir G., Yazar A.S., Arica V. (2015). *Lactobacillus reuteri* DSM 17938 shortens acute infectious diarrhea in a pediatric outpatient setting. J. Pediatr..

[56] Sindhu K.N., Sowmyanarayanan T.V., Paul A., Babji S., Ajjampur S.S., Priyadarshini S., Sarkar R., Balasubramanian K.A., Wanke C.A., Ward H.D. (2014). Immune response and intestinal permeability in children with acute gastroenteritis treated with *Lactobacillus rhamnosus* GG: A randomized, double-blind, placebo-controlled trial. Clin. Infect. Dis..

[57] Dinleyici E.C., Vandenplas Y. (2014). *Lactobacillus reuteri* DSM 17938 effectively reduces the duration of acute diarrhoea in hospitalised children. Acta Paediatr..

[58] Huang Y.F., Liu P.Y., Chen Y.Y., Nong B.R., Huang I.F., Hsieh K.S., Chen K.T. (2014). Three-combination probiotics therapy in children with salmonella and rotavirus gastroenteritis. J. Clin. Gastroenterol..

[59] Azim K., Sheikh T.S., Khan S.N. (2014). Efficacy of probiotics (*Saccharomyces bulardii*) in acute watery diarrhoea in children. J. Rawalpindi Med. Coll..

[60] Aggarwal S., Upadhyay A., Shah D., Teotia N., Agarwal A., Jaiswal V. (2014). Lactobacillus GG for treatment of acute childhood diarrhoea: An open labelled, randomized controlled trial. Indian J. Med. Res..

[61] Phavichitr N., Puwdee P., Tantibhaedhyangkul R. (2013). Cost-benefit analysis of the probiotic treatment of children hospitalized for acute diarrhea in Bangkok, Thailand. Southeast Asian J. Trop. Med. Public Health.

[62] Dinleyici E.C., Dalgic N., Guven S., Ozen M., Kara A., Arica V., Metin-Timur O., Sancar M., Kurugol Z., Tanir G. (2013). The effect of a multispecies synbiotic mixture on the duration of diarrhea and length of hospital stay in children with acute diarrhea in Turkey: Single blinded randomized study. Eur. J. Pediatr..

[63] Burande M. (2013). Comparison of efficacy of *Saccharomyces boulardii* strain in the treatment of acute diarrhea in children: A prospective, single-blind, randomized controlled clinical trial. J. Pharmacol. Pharmacother..

[64] Riaz M., Alam S., Malik A., Ali S.M. (2012). Efficacy and safety of *Saccharomyces boulardii* in acute childhood diarrhea: A double blind randomised controlled trial. Indian J. Pediatr..

[65] Nixon A.F., Cunningham S.J., Cohen H.W., Crain E.F. (2012). The effect of Lactobacillus GG on acute diarrheal illness in the pediatric emergency department. Pediatr. Emerg. Care.

[66] Khan A., Javed T., Chishti A.L. (2012). Clinical efficacy of use of probiotic “*Saccharomyces boulardii*” in children with acute watery diarrhea. Pak. Paediatr. J..

[67] Francavilla R., Lionetti E., Castellaneta S., Ciruzzi F., Indrio F., Masciale A., Fontana C., La Rosa M.M., Cavallo L., Francavilla A. (2012). Randomised clinical trial: *Lactobacillus reuteri* DSM 17938 vs. placebo in children with acute diarrhoea—A double-blind study. Aliment. Pharmacol. Ther..

[68] Erdoğan O., Tanyeri B., Torun E., Gönüllü E., Arslan H., Erenberk U., Oktem F. (2012). The comparition of the efficacy of two different probiotics in rotavirus gastroenteritis in children. J. Trop. Med..

[69] Vandenplas Y., De Hert S.G. (2011). Randomised clinical trial: The synbiotic food supplement Probiotical vs. placebo for acute gastroenteritis in children. Aliment. Pharmacol. Ther..

[70] Dutta P., Mitra U., Dutta S., Rajendran K., Saha T.K., Chatterjee M.K. (2011). Randomised controlled clinical trial of *Lactobacillus sporogenes* (*Bacillus coagulans*), used as probiotic in clinical practice, on acute watery diarrhoea in children. Trop. Med. Int. Health.

[71] Dalgic N., Sancar M., Bayraktar B., Pullu M., Hasim O. (2011). Probiotic, zinc and lactose-free formula in children with rotavirus diarrhea: Are they effective?. Pediatr. Int..

[72] Corrêa N.B., Penna F.J., Lima F.M., Nicoli J.R., Filho L.A. (2011). Treatment of acute diarrhea with *Saccharomyces boulardii* in infants. J. Pediatr. Gastroenterol. Nutr..

[73] Ritchie B.K., Brewster D.R., Tran C.D., Davidson G.P., McNeil Y., Butler R.N. (2010). Efficacy of Lactobacillus GG in aboriginal children with acute diarrhoeal disease: A randomised clinical trial. J. Pediatr. Gastroenterol. Nutr..

[74] Rerksuppaphol S., Rerksuppaphol L. (2010). *Lactobacillus acidophilus* and *Bifidobacterium bifidum* stored at ambient temperature are effective in the treatment of acute diarrhoea. Ann. Trop. Paediatr..

[75] Grandy G., Medina M., Soria R., Terán C.G., Araya M. (2010). Probiotics in the treatment of acute rotavirus diarrhoea. A randomized, double-blind, controlled trial using two different probiotic preparations in Bolivian children. BMC Infect. Dis..

[76] Chen C.C., Kong M.S., Lai M.W., Chao H.C., Chang K.W., Chen S.Y., Huang Y.C., Chiu C.H., Li W.C., Lin P.Y. (2010). Probiotics have clinical, microbiologic, and immunologic efficacy in acute infectious diarrhea. Pediatr. Infect. Dis. J..

[77] Misra S., Sabui T.K., Pal N.K. (2009). A randomized controlled trial to evaluate the efficacy of Lactobacillus GG in infantile diarrhea. J. Pediatr..

[78] Teran C.G., Teran-Escalera C.N., Villarroel P. (2009). Nitazoxanide vs. probiotics for the treatment of acute rotavirus diarrhea in children: A randomized, single-blind, controlled trial in Bolivian children. Int. J. Infect. Dis..

[79] Kianifar H.R., Farid R., Ahanchian H., Jabbari F., Moghiman T., Sistanian A. (2009). Probiotics in the treatment of acute diarrhea in young children. Iran. J. Med. Sci..

[80] Basu S., Paul D.K., Ganguly S., Chatterjee M., Chandra P.K. (2009). Efficacy of high-dose *Lactobacillus rhamnosus* GG in controlling acute watery diarrhea in Indian children: A randomized controlled trial. J. Clin. Gastroenterol..

[81] Rafeey M., Ostadrahimi A., Boniadi M., Ghorashi Z., Alizadeh M.M., Hadafey V. (2008). *Lactobacillus acidophilus* yogurt and supplement in children with acute diarrhea: A clinical trial. Res. J. Med. Sci..

[82] Narayanappa D. (2008). Randomized double blinded controlled trial to evaluate the efficacy and safety of Bifilac in patients with acute viral diarrhea. Indian J. Pediatr..

[83] Mao M., Yu T., Xiong Y., Wang Z., Liu H., Gotteland M., Brunser O. (2008). Effect of a lactose-free milk formula supplemented with bifidobacteria and streptococci on the recovery from acute diarrhoea. Asia Pac. J. Clin. Nutr..

[84] Villarruel G., Rubio D.M., Lopez F., Cintioni J., Gurevech R., Romero G., Vandenplas Y. (2007). *Saccharomyces boulardii* in acute childhood diarrhoea: A randomized, placebo-controlled study. Acta Paediatr..

[85] Ozkan T.B., Sahin E., Erdemir G., Budak F. (2007). Effect of *Saccharomyces boulardii* in children with acute gastroenteritis and its relationship to the immune response. J. Int. Med. Res..

[86] Canani R.B., Cirillo P., Terrin G., Cesarano L., Spagnuolo M.I., De Vincenzo A., Albano F., Passariello A., De Marco G., Manguso F. (2007). Probiotics for treatment of acute diarrhoea in children: Randomised clinical trial of five different preparations. BMJ.

[87] Henker J., Blokhin B.M., Bolbot Y.K., Maydannik V.G., Wolff C., Schulze L. (2006). The probiotic *E. coli* line Nissle 1917 (EcN) stops acute diarrhoea in infants and small children. Results of a confirming study. Z. Gastroenterol..

[88] Basu S., Chatterjee M., Ganguly S., Chandra P.K. (2007). Efficacy of *Lactobacillus rhamnosus* GG in acute watery diarrhoea of Indian children: A randomised controlled trial. J. Paediatr. Child Health.

[89] Vivatvakin B., Kowitdamrong E. (2006). Randomized control trial of live *Lactobacillus acidophilus* plus *Bifidobacterium infantis* in treatment of infantile acute watery diarrhea. J. Med. Assoc. Thail..

[90] Szymański H., Pejcz J., Jawień M., Chmielarczyk A., Strus M., Heczko P.B. (2006). Treatment of acute infectious diarrhoea in infants and children with a mixture of three *Lactobacillus rhamnosus* strains—A randomized, double-blind, placebo-controlled trial. Aliment. Pharmacol. Ther..

[91] Billoo A.G., Memon M.A., Khaskheli S.A., Murtaza G., Iqbal K., Saeed Shekhani M., Siddiqi A.Q. (2006). Role of a probiotic (*Saccharomyces boulardii*) in management and prevention of diarrhoea. World J. Gastroenterol..

[92] Sarker S.A., Sultana S., Fuchs G.J., Alam N.H., Azim T., Brüssow H., Hammarström L. (2005). *Lactobacillus paracasei* strain ST11 has no effect on rotavirus but ameliorates the outcome of nonrotavirus diarrhea in children from Bangladesh. Pediatrics.

[93] Kurugöl Z., Koturoğlu G. (2005). Effects of *Saccharomyces boulardii* in children with acute diarrhoea. Acta Paediatr..

[94] Kowalska-Duplaga K., Fyderek K., Szajewska H., Janiak R. (2004). Efficacy of Trilac^®^ in the treatment of acute diarrhoea in infants and young children—A multicentre, randomized, double-blind placebo-controlled study. Pediatr. Wspolczesna.

[95] Costa-Ribeiro H., Ribeiro T.C., Mattos A.P., Valois S.S., Neri D.A., Almeida P., Cerqueira C.M., Ramos E., Young R.J., Vanderhoof J.A. (2003). Limitations of probiotic therapy in acute, severe dehydrating diarrhea. J. Pediatr. Gastroenterol. Nutr..

[96] Rosenfeldt V., Michaelsen K.F., Jakobsen M., Larsen C.N., Møller P.L., Tvede M., Weyrehter H., Valerius N.H., Paerregaard A. (2002). Effect of probiotic Lactobacillus strains on acute diarrhea in a cohort of nonhospitalized children attending day-care centers. Pediatr. Infect. Dis. J..

[97] Rosenfeldt V., Michaelsen K.F., Jakobsen M., Larsen C.N., Møller P.L., Pedersen P., Tvede M., Weyrehter H., Valerius N.H., Paerregaard A. (2002). Effect of probiotic *Lactobacillus strains* in young children hospitalized with acute diarrhea. Pediatr. Infect. Dis. J..

[98] Hafeez A., Tariq P., Ali S., Kundi Z.U., Khan A., Hassan M. (2002). The efficacy of *Saccharomyces boulardii* in the treatment of acute watery diarrhea in children: A multicentre randomized controlled trial. J. Coll. Physicians Surg. Pak..

[99] Urganci N., Polat T., Uysalol M., Cetinkaya F. (2001). Evaluation of the efficacy of *Saccharomyces boulardii* in children with acute diarrhoea. Arch. Gastroenterohepatol..

[100] Lee M.C., Lin L.H., Hung K.L., Wu H.Y. (2001). Oral bacterial therapy promotes recovery from acute diarrhea in children. Acta Paediatr. Taiwanica.

[101] Boudraa G., Benbouabdellah M., Hachelaf W., Boisset M., Desjeux J.F., Touhami M. (2001). Effect of feeding yogurt versus milk in children with acute diarrhea and carbohydrate malabsorption. J. Pediatr. Gastroenterol. Nutr..

[102] Guandalini S., Pensabene L., Zikri M.A., Dias J.A., Casali L.G., Hoekstra H., Kolacek S., Massar K., Micetic-Turk D., Papadopoulou A. (2000). Lactobacillus GG administered in oral rehydration solution to children with acute diarrhea: A multicenter European trial. J. Pediatr. Gastroenterol. Nutr..

[103] Hernandez C.L., Pineda E.E., Jimenez M.I.R., Lucena M.S. (1998). Clinical therapeutic affect of *Saccharomyces boulardii* on children with acute diarrhea. Rev. Enferm. Infecc. Pediatr..

[104] Shornikova A.V., Isolauri E., Burkanova L., Lukovnikova S., Vesikari T. (1997). A trial in the Karelian Republic of oral rehydration and Lactobacillus GG for treatment of acute diarrhoea. Acta Paediatr..

[105] Shornikova A.V., Casas I.A., Mykkänen H., Salo E., Vesikari T. (1997). Bacteriotherapy with *Lactobacillus reuteri* in rotavirus gastroenteritis. Pediatr. Infect. Dis. J..

[106] Shornikova A.V., Casas I.A., Isolauri E., Mykkänen H., Vesikari T. (1997). *Lactobacillus reuteri* as a therapeutic agent in acute diarrhea in young children. J. Pediatr. Gastroenterol. Nutr..

[107] Guarino A., Canani R.B., Spagnuolo M.I., Albano F., Di Benedetto L. (1997). Oral bacterial therapy reduces the duration of symptoms and of viral excretion in children with mild diarrhea. J. Pediatr. Gastroenterol. Nutr..

[108] Pant A.R., Graham S.M., Allen S.J., Harikul S., Sabchareon A., Cuevas L., Hart C.A. (1996). Lactobacillus GG and acute diarrhoea in young children in the tropics. J. Trop. Pediatr..

[109] Isolauri E., Kaila M., Mykkänen H., Ling W.H., Salminen S. (1994). Oral bacteriotherapy for viral gastroenteritis. Dig. Dis. Sci..

[110] Cetina-Sauri G., Sierra Basto G. (1994). Evaluation of *Saccharomyces boulardii* for the treatment of acute diarrhea in pediatric patients. Ann. Pediatr..

[111] Baral R., Nonvignon J., Debellut F., Agyemang S.A., Clark A., Pecenka C. (2020). Cost of illness for childhood diarrhea in low- and middle-income countries: A systematic review of evidence and modelled estimates. BMC Public Health.

[112] Patro-Gołąb B., Szajewska H. (2019). Systematic Review with Meta-Analysis: *Lactobacillus reuteri* DSM 17938 for Treating Acute Gastroenteritis in Children. An Update. Nutrients.

[113] Rannikko J., Holmberg V., Karppelin M., Arvola P., Huttunen R., Mattila E., Kerttula N., Puhto T., Tamm U., Koivula I. (2021). Fungemia and Other Fungal Infections Associated with Use of *Saccharomyces boulardii* Probiotic Supplements. Emerg. Infect. Dis..

[114] Uusitupa H.M., Rasinkangas P., Lehtinen M.J., Makela S.M., Airaksinen K., Anglenius H., Ouwehand A.C., Maukonen J. (2020). *Bifidobacterium animalis* subsp. *lactis* 420 for Metabolic Health: Review of the Research. Nutrients.

[115] Derrien M., van Hylckama Vlieg J.E. (2015). Fate, activity, and impact of ingested bacteria within the human gut microbiota. Trends Microbiol..

[116] Morgan R.L., Preidis G.A., Kashyap P.C., Weizman A.V., Sadeghirad B., McMaster Probiotic P., Synbiotic Work G. (2020). Probiotics Reduce Mortality and Morbidity in Preterm, Low-Birth-Weight Infants: A Systematic Review and Network Meta-analysis of Randomized Trials. Gastroenterology.

[117] Van den Akker C.H.P., van Goudoever J.B., Szajewska H., Embleton N.D., Hojsak I., Reid D., Shamir R. (2018). Probiotics for Preterm Infants: A Strain-Specific Systematic Review and Network Meta-analysis. J. Pediatr. Gastroenterol. Nutr..

[118] Tan-Lim C.S.C., Esteban-Ipac N.A.R., Mantaring J.B.V., Chan Shih Yen E., Recto M.S.T., Sison O.T., Alejandria M.M. (2021). Comparative effectiveness of probiotic strains for the treatment of pediatric atopic dermatitis: A systematic review and network meta-analysis. Pediatr. Allergy Immunol..

