# Supplementary material for: Which Probiotic Is the Most Effective for Treating Acute Diarrhea in Children? A Bayesian Network Meta-Analysis of Randomized Controlled Trials
Source: Nutrients. 2021 Nov 29;13(12):4319. doi: 10.3390/nu13124319 (PMC8706888; doi:10.3390/nu13124319)
Supplement: Supplementary file 1 [file nutrients-13-04319-s001.zip › nutrients-1429194-supplementary.pdf]

Supplementary Materials

Which Probiotic Is the Most Effective for Treating Acute Diarrhea in Children? A Bayesian Network Meta-Analysis of Randomized Controlled Trials

Zengbin Li[1], Guixian Zhu[1], Chao Li[2], Hao Lai[1], Xin Liu[2], Lei Zhang[1,3,4,5,*]

[1] China-Australia Joint Research Center for Infectious Diseases, School of Public Health, Xi'an Jiaotong University Health Science Center, Xi'an, 710061, Shaanxi, People's Republic of China; lizengbin98@126.com (Z.L.); xianxianshell@163.com (G.Z.); xjtu_haolai@163.com (H.L.)

[2] Department of Epidemiology and Biostatistics, School of Public Health, Global Health Institute, Xi'an Jiaotong University Health Science Center, Xi'an, 710061, Shaanxi, People's Republic of China; lcxjtu@xjtu.edu.cn (C.L.); xinliu@xjtu.edu.cn (X.L.)

[3] Melbourne Sexual Health Centre, Alfred Health, Melbourne, Australia.

[4] Central Clinical School, Faculty of Medicine, Nursing and Health Sciences, Monash University, Melbourne, Australia.

[5] Department of Epidemiology and Biostatistics, College of Public Health, Zhengzhou University, Zhengzhou, Henan, People's Republic of China.

[*] Correspondence: lei.zhang1@monash.edu (L.Z.); Tel.: (+8629-82655135)

Online Supplementary Contents

Table S1. Search strategies……………………………………………………………….……….…1
Table S2. Characteristics of included studies. 2
References 8
Figure S1. Risk of bias graph 12
Figure S2. Risk of bias summary.. 13
Figure S3. Incoherence plot for the duration of diarrhea (control = placebo/no treatment) 14
Figure S4. Incoherence plot for the duration of diarrhea (control = no treatment)................................ 15
Figure S5. Incoherence plot for the duration of hospitalization 16
Figure S6. Incoherence plot for the mean stool frequency on day 2 16
Figure S7. Incoherence plot for the duration of vomiting 17
Figure S8. Incoherence plot for the duration of fever... 17
Table S3. Heterogeneity for the duration of diarrhea (control = placebo/no treatment)......................... 18
Table S4. Heterogeneity for the duration of diarrhea (control = placebo)... 19
Table S5. Heterogeneity for the duration of diarrhea (control = no treatment) 20
Table S6. Heterogeneity for diarrhea lasting ≥2 days... 21
Table S7. Heterogeneity for the duration of hospitalization.. 22
Table S8. Heterogeneity for the mean stool frequency on day 2 23
Table S9. Heterogeneity for the duration of vomiting... 24
Table S10. Heterogeneity for the duration of fever 25
Table S11. NMA results for the duration of diarrhea (control = placebo/no treatment)......................... 26
Table S12. NMA results for the duration of diarrhea (control = placebo)... 28
Table S13. NMA results for the duration of diarrhea (control = no treatment) 30
Table S14. NMA results for diarrhea lasting ≥2 days 31
Table S15. NMA results for the duration of hospitalization.. 32
Table S16. NMA results for the mean stool frequency on day 2.. 33
Table S17. NMA results for the duration of vomiting... 34
Table S18. NMA results for the duration of fever.. 35
Table S19. Certainty of evidence for the duration of diarrhea.. 36
Table S20. Certainty of evidence for the duration of diarrhea (control = placebo) 37
Table S21. Certainty of evidence for the duration of diarrhea (control = no treatment)......................... 38
Table S22. Certainty of evidence for diarrhea lasting ≥2 days... 39
Table S23. Certainty of evidence for the duration of hospitalization 40
Table S24. Certainty of evidence for the mean stool frequency on day 2 41
Table S25. Certainty of evidence for the duration of vomiting 42
Table S26. Certainty of evidence for the duration of fever 43
Table S27. Rank for outcomes 44

Table S1. Search strategies

We searched Embase, PubMed, and the Cochrane Library using a combination of MeSH and free text. Besides, we chose the title/abstract to limit the scope. The search terms include: "diarrhea", "probiotic", "children", and "randomized controlled trial". In order to include relevant literatures that meet the inclusion criteria as much as possible, we did not add "randomized controlled trial" to search in the Cochrane Library.

Search terms related to diarrhea	Diarrhea; diarrheas; diarrhea; diarrheas; acute diarrhea; diarrh*; gastroenteritis; gastroenteritides; acute gastroenteritis
Search terms related to probiotic	Probiotic; probiotics; probiotic*; prebiotics; prebiotic; prebiotic*; *bifidobacterial; bifidobacterium*; *lactobacillaceae*; *lactobacilli*; *lactobacillus*; *saccharomyces*; *saccharomyces*; *saccharomyc**; *saccharomyces boulardii*; *lactobacillus*; *enterococcus*; *escherichia coli*; *streptococcus*; *lactococcus*; *bacillus*; *lactobacilli*; *lactobacillus rhamnosus GG*; *lactobacillus reuteri*; *lactobacillus sporogenes*; *bifidobacterium longum*; *bifidobacterium breve*; *bifidobacterium bifidum*; *bifidobacterium lactis*; *bacillus*; *bacillus clausii*; *lactobacill**; *streptococcus thermophilus*; *streptococc**; *lactococc**; *bacillus subtilis*; *enterococcus*; *enterococcus faec**; *leuconostoc*; *pediococc**; *bulgarian bacillus*; *bacillus laterosporus*; *pediococcus acidilactici*; *lactis*
Search terms related to children	Children; child; child*; kids; kid; neonat; neonat*; baby; babies; pediatric; paediatric; infant; infants; toddler*; toddler; toddlers; adolescent; adolescents; adolescence; adolescen*; newborns; adolescen*; teenag*; youth*; young; preschooler; preschoolers; juvenile; juvenile*; pubescent; youth; teen; teens; teenage; teen-age; teenaged; teen-aged; teenager; teenagers; teen-ager; teen-agers; youngster; youngsters; minor; minors; infan*; newborn*; new-born*; infant, newborn; infants, newborn; newborn infant; newborn infants; newborns; newborn; neonate; neonates; infantile
Search terms related to randomized controlled trial	Randomized; random; randomly; random*; randomized controlled trial; clinical trials, randomized; trials, randomized clinical; controlled clinical trial; placebo; placebo*; trial; groupscrossover procedure; double-blind procedure; single-blind procedure; groups; assign*; allocat*; single blind; double blind; triple blind

Table S2. Characteristics of included studies

Study	Study Site	Intervention	N	Duration of hospitalization(day)	Duration of Diarrhea(day)	Duration of Fever(day)	Duration of Vomiting(day)	Day 2 Stool Frequency	Diarrhea lasting ≥2 days(N)
Vaghela 2020 [1]	India	*S. boulardii*	50	NA	NA	NA	NA	NA	44
		No treatment	50	NA	NA	NA	NA	NA	49
Shin 2020 [2]	Korea	*L. plantarum*	15	4.17±0.86	1.0±0.57	1.27±1.64	0.64±0.82	NA	NA
		No treatment	8	3.94±0.61	2.23±0.35	1.50±2.23	0.72±0.67	NA	NA
Mourey 2020 [3]	India	*S. boulardii*	49	NA	2.74±0.5	NA	NA	3.1±0.9	8
		Placebo	51	NA	3.97±0.73	NA	NA	3.6±1.3	36
Kluijfhout 2020 [4]	Belgium	*S. thermophilus + L. rhamnosus + L. acidophilus + B. lactis + B. infantis*	26	NA	3.04±1.36	NA	NA	NA	15
		Placebo	20	NA	4.2±1.34	NA	NA	NA	17
Chen 2020 [5]	China	*B. lactis + L. rhamnosus + L. acidophilus*	96	5.1±1.2	5.06±0.57	NA	NA	4.4±1.3	NA
		No treatment	98	6.3±1.4	6.0±0.83	NA	NA	4.7±1.9	NA
Szymanski 2019 [6]	Poland	*L. reuteri*	44	2.56±0.90	2.45±1.83	NA	NA	2.40±3.30	4
		Placebo	44	2.81±1.16	2.79±2.39	NA	NA	2.35±3.82	2
Sudha 2019	India	*Bacillus clausii*	59	NA	3.14±0.54	1.57±0.51	NA	6.84±1.76	54

[7]		Placebo	60	NA	3.4±0.64	1.60±0.58	NA	7.38±1.94	58
Islam 2019 [8]	Bangladesh	*Bacillus clausii*	160	3.8±1.4	3.2±1.3	NA	NA	5.8±2.0	NA
		No treatment	150	3.8±1.0	3.3±1.1	NA	NA	6.0±1.7	NA
Vidjeadevan 2018 [9]	India	*S. boulardii*	34	3.41±1.04	3.36±0.77	NA	NA	NA	NA
		Bacillus clausii	33	3.06±0.86	3.64±0.78	NA	NA	NA	NA
		No treatment	32	3.34±1.06	4.50±0.78	NA	NA	NA	NA
Schnadower 2018 [10]	USA	LGG	468	NA	2.53±2.45	NA	NA	2.46±2.81	243
		Placebo	475	NA	2.65±2.32	NA	NA	2.57±2.61	251
Javeed 2018 [11]	Pakistan	*S. boulardii*	157	NA	4.37±1.38	NA	NA	NA	157
		Placebo	157	NA	4.59±1.50	NA	NA	NA	157
Hong Chau 2018 [12]	Vietnam	*L. acidophilus*	150	3.29±1.56	3.36±1.78	NA	NA	NA	54
		Placebo	150	3.26±1.59	3.33±1.72	NA	NA	NA	60
Freedman 2018 [13]	Multicenter	*L. rhamnosus + L. helveticus*	414	NA	2.81±2.75	NA	NA	2.46±0.14	228
		Placebo	413	NA	3.0±2.92	NA	NA	2.81±0.19	225
Bhat 2018 [14]	India	*Bacillus clausii*	40	3.26±0.69	2.22±0.70	0.51±0.34	NA	3.98±1.40	NA
		S. boulardii	40	2.72±0.42	1.74±0.45	0.44±0.30	NA	4.48±1.77	NA
		No treatment	40	3.37±1.10	2.40±1.10	0.97±0.96	NA	4.18±2.63	NA
Sirsat 2017 [15]	India	*S. boulardii*	145	NA	NA	NA	NA	NA	82
		No treatment	145	NA	NA	NA	NA	NA	54

Park 2017 [16]	Korea	*L. acidophilus* +*B. longum*	28	NA	4.38±1.29	3.66±1.14	NA	NA	NA
		Placebo	29	NA	5.61±1.23	4.32±1.94	NA	NA	NA
Burki 2017 [17]	Pakistan	*S. boulardii*	100	NA	3.23±1.31	NA	NA	NA	60
		No treatment	100	NA	5.84±1.81	NA	NA	NA	80
Yazar 2016 [18]	Turkey	*L. casei* + *L. rhamnosus*+ *L. plantarum* + *B. lactis*	55	NA	3.79±1.20	NA	NA	NA	34
		No treatment	55	NA	4.76±1.29	NA	NA	NA	46
Sharif 2016 [19]	Iran	*S. boulardii*	100	NA	3.4±1.3	NA	NA	6.5±1.8	NA
		Placebo	100	NA	5.5±2.1	NA	NA	7.2±3.5	NA
Garcia-Menor 2016 [20]	Multicenter	*L. casei* + *L. rhamnosus* + *S. thermophilus* + *B. breve* + *L. acidophilus* + *B. infantis* + *B. bulgaricus*	43	NA	3.35±2.30	NA	NA	NA	19
		No treatment	42	NA	4.0±1.54	NA	NA	NA	20
Dash 2016 [21]	India	*S. boulardii*	64	NA	1.1±2.0	NA	NA	NA	NA
		No treatment	62	NA	2.03±2.0	NA	NA	NA	NA
Das 2016 [22]	India	*S. boulardii*	30	3.17±0.84	2.50±0.20	2.38±0.62	1.99±0.55	NA	NA
		Placebo	28	3.78±0.94	3.72±0.20	2.82±0.85	2.20±0.59	NA	NA
Lee 2015 [23]	Korea	*B. longum* + *B. lactis* + *L*+ *acidophilus* + *L. rhamnosus* + *L. plantarum* + *Pediococcus pentosaceus*	13	NA	6.1±0.5	1.1±1.9	1.6±1.6	NA	NA

		Placebo	16	NA	7.2±1.9	1.6±1.7	2.8±1.8	NA	NA
Hegar 2015 [24]	Indonesia	*L. rhamnosus + L. acidophilus*	56	NA	2.99±1.38	NA	NA	NA	NA
		Placebo	56	NA	2.78±1.32	NA	NA	NA	NA
Freedman 2015 [25]	Canada	*L. helveticus + L. rhamnosus*	61	NA	2.96±3.26	NA	1.51±1.93	NA	NA
		Placebo	62	NA	2.65±2.68	NA	1.57±1.80	NA	NA
El-Soud 2015 [26]	Egypt	*B. lactis*	25	NA	3.12±0.92	2.27±0.85	NA	NA	NA
		Placebo	25	NA	4.10±0.94	2.79±0.64	NA	NA	NA
Dinleyici 2015-1 [27]	Turkey	*S. boulardii*	220	4.60±1.72	3.14±1.38	NA	NA	NA	141
		No treatment	143	6.12±1.71	4.16±1.35	NA	NA	NA	120
Dinleyici 2015-2 [28]	Turkey	*L. reuteri*	29	NA	2.52±1.02	NA	NA	NA	13
		No treatment	31	NA	3.10±0.64	NA	NA	NA	27
Sindhu 2014 [29]	India	LGG	65	NA	4.35±2.27	NA	NA	NA	NA
		Placebo	59	NA	4.35±2.28	NA	NA	NA	NA
Dinleyici 2014 [30]	Turkey	*L. reuteri*	64	4.31±1.30	2.95±1.09	NA	NA	NA	32
		No treatment	63	5.46±1.77	4.33±1.18	NA	NA	NA	60
Huang 2014 [31]	China	*Bacillus mesentericus+ Clostridium butyricum+ Enterococcus faecalis*	82	5.7±2.4	1.8±1.6	2.2±1.5	NA	NA	NA
		No treatment	77	5.2±2.3	2.9±1.4	2.0±1.5	NA	NA	NA
Azim 2014	Pakistan	*S. boulardii*	45	3.09±0.46	NA	NA	NA	3.56±0.84	NA

[32]		No treatment	45	5.07±0.93	NA	NA	NA	5.31±0.73	NA
Aggarwal 2014 [33]	India	*LGG*	100	3.33±0.56	2.59±0.56	NA	0.62±0.48	NA	NA
		No treatment	100	3.84±0.65	3.34±0.56	NA	0.81±0.43	NA	NA
Phavichitr 2013 [34]	Thailand	*L. acidophilus + B. bifidum*	53	2.35±0.76	4.35±2.29	NA	NA	NA	NA
		Placebo	53	3.0±1.52	5.0±1.52	NA	NA	NA	NA
Dinleyici 2013 [35]	Turkey	*L. acidophilus + L. rhamnosus + B. bifidum, B. longum + Enterococcus faecium*	113	4.94±1.70	3.25±1.27	NA	NA	3.38 ±1.6	82
		No treatment	96	5.77±1.97	4.78±1.56	NA	NA	4.65±3.61	90
Burande 2013 [36]	India	*S. boulardii*	35	NA	3.4±1.4	NA	NA	NA	NA
		No treatment	35	NA	5.5±2.1	NA	NA	NA	NA
Riaz 2012 [37]	India	*S. boulardii*	43	NA	2.06±0.99	NA	NA	10.96±8.04	NA
		Placebo	47	NA	2.76±1.22	NA	NA	16.21±17.39	NA
Nixon 2012 [38]	USA	LGG	63	NA	2.82±1.50	NA	NA	NA	NA
		Placebo	66	NA	3.08±1.65	NA	NA	NA	NA
Khan 2012 [39]	Pakistan	*S. boulardii*	210	NA	3.43±5.58	NA	NA	NA	11
		No treatment	210	NA	4.50±5.58	NA	NA	NA	120
Francavilla 2012 [40]	Italy	*L. reuteri*	35	NA	2.1±1.7	NA	NA	4.3±1.7	16
		Placebo	34	NA	3.3±2.1	NA	NA	6.3±2.1	25
Erdogan 2012	Turkey	*S. boulardii*	25	NA	6.6±1.7	NA	NA	NA	NA

[41]		*B. lactis*	25	NA	4.1±1.3	NA	NA	NA	NA
		No treatment	25	NA	7.0±1.6	NA	NA	NA	NA
Vandenplas 2011 [42]	Belgium	*S. thermophilus* + *L. rhamnosus* + *L. acidophilus* + *B. lactis* + *B. infantis*	57	NA	3.05±1.28	NA	NA	3.14±1.52	25
		Placebo	54	NA	4.31±0.83	NA	NA	3.69±1.99	40
Dutta 2011 [43]	India	*L. sporogenes*	78	NA	1.42±0.85	NA	NA	NA	NA
		Placebo	70	NA	1.52±0.89	NA	NA	NA	NA
Dalgic 2011 [44]	Turkey	*S. boulardii*	60	5.30±1.73	4.78±1.46	NA	0.85±0.54	NA	NA
		Placebo	60	5.81±2.08	5.35±1.80	NA	0.68±0.47	NA	
Correa 2011 [45]	Brazil	*S. boulardii*	90	NA	NA	NA	NA	NA	39
		Placebo	86	NA	NA	NA	NA	NA	69
Ritchie 2010 [46]	Australia	LGG	33	NA	2.18±2.08	NA	NA	3.30±2.54	13
		Placebo	31	NA	2.13±1.77	NA	NA	4.70±2.59	12
Rerksuppaphol 2010 [47]	Thailand	*L. acidophilus* + *B. bifidum*	23	NA	NA	NA	NA	NA	4
		Placebo	22	NA	NA	NA	NA	NA	11
Grandy 2010 [48]	Bolivia	*S. boulardii*	21	NA	2.92±1.21	NA	NA	NA	NA
		L. acidophilus + *L. rhamnosus* + *B. longum* + *S. boulardii*	23	NA	2.50±1.23	NA	NA	NA	NA
		Placebo	20	NA	5.68±1.76	NA	NA	NA	NA
Chen 2010	China	*Bacillus mesentericus* +	150	2.9±0.8	2.50±1.32	1.73±0.98	1.18±0.88	2.72±1.25	NA

[49]		*Enterococcus faecalis + Clostridium butyricum*							
		Placebo	143	4.2±2.1	3.60±1.57	2.08±1.45	1.81±1.36	4.37±2.83	NA
Misra 2009 [50]	India	LGG	105	NA	2.94±0.98	NA	NA	NA	NA
		Placebo	105	NA	3.25±1.43	NA	NA	NA	NA
Teran 2009 [51]	Bolivia	*L. acidophilus + L. rhamnosus + B. longum + S. boulardii*	25	3.46±1.40	2.38±1.06	1.0±1.13	NA	6.79±7.08	7
		No treatment	25	4.20±1.14	3.11±1.11	1.0±0.83	NA	7.36±4.72	16
Kianifar 2009 [52]	Iran	*L. acidophilus + B. bifidum*	32	2.1±0.7	3.4±0.8	NA	NA	NA	NA
		Placebo	30	2.7±0.6	4.5±0.8	NA	NA	NA	NA
Basu 2009 [53]	India	LGG	374	6.22±1.16	5.07±1.24	NA	4.12±1.47	22.99±6.09	NA
		No treatment	185	9.75±2.06	7.23±1.27	NA	4.18±1.58	23.49±6.10	NA
Rafeey 2008 [54]	Iran	*L. acidophilus*	40	3.4±0.9	NA	NA	NA	4.0±3.2	NA
		Placebo	40	4.0±1.1	NA	NA	NA	4.0±3.6	NA
Narayanappa 2008 [55]	India	*Bifilac*	40	NA	4.35±1.25	NA	NA	3.98±2.71	NA
		Placebo	40	NA	5.45±1.69	NA	NA	4.83±2.77	NA
Mao 2008 [56]	China	*B. lactis + S. thermophilus*	141	NA	2.79±0.15	NA	NA	NA	NA
		Placebo	71	NA	2.83±0.17	NA	NA	NA	NA
Villarruel 2007 [57]	Argentina	*S. boulardii*	44	NA	4.70±1.94	NA	NA	NA	NA
		Placebo	44	NA	6.16±3.20	NA	NA	NA	NA

Ozkan 2007 [58]	Turkey	*S. boulardii*	16	NA	NA	NA	NA	3.06±0.33	NA
		Placebo	11	NA	NA	NA	NA	4.27±0.38	NA
Canani 2007 [59]	Italy	LGG	100	NA	3.33±1.50	1.36±0.78	1.36±0.78	4.70±1.50	NA
		S. boulardii	91	NA	4.38±0.45	1.89±0.23	1.64±0.79	5.35±2.26	NA
		Bacillus clausii	100	NA	4.7±1.0	2.0±1.56	1.50±0.78	5.35±2.26	NA
		L. bulgaricus + L. acidophilus + S. thermophilus + B. bifidum	97	NA	3.06±1.63	1.701.56±	1.36±0.77	4.70±1.51	NA
		Enterococcus faecium	91	NA	4.84±1.73	2.0±1.54	1.50±0.77	5.35±2.26	NA
		No treatment	92	NA	4.7±1.0	1.64±0.78	1.64±0.77	5.35±2.26	NA
Henker 2007 [60]	Multicenter	*Escherichia coli Nissle 1917*	55	NA	2.93±0.98	NA	NA	NA	21
		Placebo	58	NA	4.37±0.38	NA	NA	NA	32
Basu 2007 [61]	India	LGG	323	9.3±1.3	6.8±2.1	NA	3.2±1.1	24.3±4.8	NA
		Placebo	323	9.2±1.2	6.6±2.3	NA	3.3±1.2	24.2±5.3	NA
Vivatvakin 2006 [62]	Thailand	*L. acidophilus + B. infantis*	35	2.1±1.2	1.6±0.7	NA	NA	2.2±2.0	2
		No treatment	36	1.6±1.0	2.9±1.7	NA	NA	2.6±2.2	11
Szymanski 2006 [63]	Poland	*Three L. rhamnosus*	46	NA	3.48±2.32	NA	NA	3.0±2.8	NA
		Placebo	41	NA	4.0±2.98	NA	NA	2.9±2.9	NA
Billoo 2006 [64]	Parkistan	*S. boulardii*	50	NA	3.60±1.77	NA	NA	NA	NA
		No treatment	50	NA	4.80±1.77	NA	NA	NA	NA

Sarker 2005 [65]	Bangladesh	*L. paracasei*	115	NA	3.77±1.88	NA	NA	NA	NA
		Placebo	115	NA	3.93±1.80	NA	NA	NA	NA
Kurugol 2005 [66]	Turkey	*S. boulardii*	100	2.9±1.2	4.7±2.5	1.0±0.8	1.2±1.0	NA	20
		Placebo	100	3.9±1.5	5.5±3.2	1.1±0.9	1.3±1.0	NA	55
Kowalska-Duplaga 2004 [67]	Poland	*L. acidophilus + B. bifidum + L. bulgaricus*	86	4.71±1.38	2.28±1.25	NA	NA	NA	NA
		Placebo	87	5.0±2.38	2.57±1.42	NA	NA	NA	NA
Costa-Ribeiro 2003 [68]	Brazil	LGG	61	NA	1.59±0.16	NA	NA	NA	31
		Placebo	63	NA	1.63±0.19	NA	NA	NA	45
Rosenfeldt 2002-1 [69]	Denmark	*L. rhamnosus + L. reuteri*	24	NA	3.16±1.65	NA	NA	NA	NA
		Placebo	19	NA	4.82±3.54	NA	NA	NA	NA
Rosenfeldt 2002-2 [70]	Denmark	*L. rhamnosus + L. reuteri*	30	1.6±1.0	3.40±1.55	1.9±1.7	1.2±1.2	NA	NA
		Placebo	39	2.7±2.0	4.21±1.98	2.1±2	1.6±1.4	NA	NA
Hafeez 2002 [71]	Pakistan	*S. boulardii*	51	NA	3.60±1.49	NA	NA	NA	NA
		No treatment	50	NA	4.50±1.49	NA	NA	NA	NA
Urganci 2001 [72]	Turkey	*S. boulardii*	50	NA	NA	NA	NA	3.78±0.71	28
		No treatment	50	NA	NA	NA	NA	4.24±0.99	42
Lee 2001 [73]	China	*L. acidophilus + B. infantis*	50	NA	3.1±0.7	NA	NA	1.9±1.9	NA
		No treatment	50	NA	3.6±0.8	NA	NA	3.7±2.4	NA
Boudraa 2001	Algeria	*L. bulgaricus + S.*	56	NA	1.84±1.40	NA	NA	NA	9

[74]		*thermophilus*							
		Placebo	56	NA	2.57±1.48	NA	NA	NA	23
Guandalini 2000 [75]	Multicenter	LGG	147	3.28±0.93	2.43±1.15	NA	NA	NA	78
		Placebo	140	4.01±0.89	3.0±1.49	NA	NA	NA	90
Hernandez 1998 [76]	Mexico	*S. boulardii*	25	NA	NA	NA	NA	NA	5
		Placebo	25	NA	NA	NA	NA	NA	11
Shornikova 1997-1 [77]	Russia	LGG	59	7.6±5.6	2.7±2.2	NA	NA	NA	NA
		Placebo	64	9.2±6.3	3.7±2.8	NA	NA	NA	NA
Shornikova 1997-2 [78]	Finland	*L. reuteri*	41	NA	1.70±1.02	NA	NA	1.9±2.4	11
		Placebo	25	NA	2.5±1.5	NA	NA	3.8±2.8	11
Shornikova 1997-3 [79]	Finland	*L. reuteri*	19	NA	1.7±1.6	NA	NA	1.0±2.3	3
		Placebo	21	NA	2.9±2.3	NA	NA	2.5±2.3	11
Guarino 1997 [80]	Italy	LGG	52	NA	3.20±1.44	NA	NA	NA	NA
		Placebo	48	NA	5.90±1.39	NA	NA	NA	NA
Pant 1996 [81]	Thailand	LGG	14	NA	1.9±0.6	NA	NA	3.5±1.3	NA
		Placebo	12	NA	3.3±2.3	NA	NA	5.2±2.8	NA
Raza 1995 [82]	Pakistan	LGG	19	NA	NA	NA	NA	5.8±3.1	NA
		Placebo	17	NA	NA	NA	NA	7.0±3.3	NA
Isolauri 1994	Finland	LGG	21	NA	1.5±0.7	NA	NA	NA	2

[83]		No treatment	21	NA	2.3±0.8	NA	NA	NA	9
Cetina-Sauri 1994 [84]	Mexico	*S. boulardii*	65	NA	NA	NA	NA	3.76±2.31	41
		Placebo	65	NA	NA	NA	NA	4.38±2.73	58

Footnote. *Saccharomyces (S.)*; *Lactobacillus (L.)*; *Bifidobacterium (B.)*; *Lactobacillus rhamnosus* GG (LGG); Not applicable (NA).

References

[1] Vaghela P, Langade RA. Analysis of impact of ors with zinc & probiotics supplements in curing acute diarrhoea. Article. International Journal of Research in Pharmaceutical Sciences, 2020;11(3):4054-4060.

[2] Shin DY, Yi DY, Jo S, et al. Effect of a new Lactobacillus plantarum product, LRCC5310, on clinical symptoms and virus reduction in children with rotaviral enteritis. Medicine, 2020;99(38):e22192.

[3] Mourey F, Sureja V, Kheni D, et al. A Multicenter, Randomized, Double-Blind, Placebo-Controlled Trial of Saccharomyces boulardii in Infants and Children With Acute Diarrhea. Pediatric infectious disease journal, 2020.

[4] Kluijfhout S, Trieu TV, Vandenplas Y. Efficacy of the Probiotic Probiotical Confirmed in Acute Gastroenteritis. Pediatric gastroenterology, hepatology & nutrition. 2020;23(5):464-471.

[5] Chen K, Xin J, Zhang G, et al. A combination of three probiotic strains for treatment of acute diarrhoea in hospitalised children: an open label, randomised controlled trial. Beneficial microbes, 2020;11(4):339-346.

[6] Szymański H, Szajewska H. Lack of Efficacy of Lactobacillus reuteri DSM 17938 for the Treatment of Acute Gastroenteritis: a Randomized Controlled Trial. Pediatric infectious disease journal, 2019;38(10):e237-e242.

[7] Sudha MR, Jayanthi N, Pandey DC, Verma AK. Bacillus clausii UBBC-07 reduces severity of diarrhoea in children under 5 years of age: a double blind placebo controlled study. Beneficial microbes. 2019;10(2):149-154.

[8] Islam TMDT, Hussain T, Rahman A, Quaium SMMA, Hamid F. Clinical Efficacy of Bacillus Clausii Probiotic in the Management of Acute Diarrhoea in Children. Chattagram Maa-O-Shishu Hospital Medical College Journal, 2019;18(1):14-17.

[9] D V, S V, S R. Role of Saccharomyces boulardii and Bacillus clausii in reducing the duration of diarrhea: a three-armed randomised controlled trial. International Journal of Contemporary Pediatrics, 2018;5(5).

[10] Schnadower D, Tarr PI, Casper TC, et al. Lactobacillus rhamnosus GG versus Placebo for Acute Gastroenteritis in Children. New England journal of medicine, 2018;379(21):2002-2014.

[11] Javeed A, Manzoor S, Wamiq S. Effect of oral saccharomyces boulardii supplementation on the duration of acute watery diarrhea in children. Pakistan journal of medical and health sciences, 2018;12(1):212-214.

[12] Hong Chau TT, Minh Chau NN, Hoang Le NT, et al. A Double-blind, Randomized, Placebo-controlled Trial of Lactobacillus acidophilus for the Treatment of Acute Watery Diarrhea in Vietnamese Children. Pediatric infectious disease journal, 2018;37(1):35-42.

[13] Freedman SB, Williamson-Urquhart S, Farion KJ, et al. Multicenter Trial of a Combination Probiotic for Children with Gastroenteritis. New England journal of medicine, 2018;379(21):2015-2026.

[14] Bhat S, G. N. S, Savio CD. Efficacy of probiotics in acute diarrhoea in children. International Journal of Contemporary Pediatrics, 2018;5(4):1646-1650.

[15] Sirsat GM, Sankpal DM. Role of Saccharomyces boulardii in management of acute diarrhoea of children - A randomized controlled trial. MedPulse International Journal of Pediatrics, 2017;4(3):68-72.

[16] Park MS, Kwon B, Ku S, Ji GE. The Efficacy of Bifidobacterium longum BORI and Lactobacillus

acidophilus AD031 Probiotic Treatment in Infants with Rotavirus Infection. Nutrients, 2017;9(8).

[17] Burki MFK, Jabeen F. Efficacy of saccharomyces boullardii in children with acute diarrhea. Medical forum monthly, 2017;28(2):112-116.

[18] Yazar AS, Güven Ş, Dinleyici E. Effects of zinc or synbiotic on the duration of diarrhea in children with acute infectious diarrhea. Turkish journal of gastroenterology, 2016;27(6):537-540.

[19] Sharif MR, Kashani HH, Ardakani AT, Kheirkhah D, Tabatabaei F, Sharif A. The Effect of a Yeast Probiotic on Acute Diarrhea in Children. Probiotics and antimicrobial proteins, 2016;8(4):211-214.

[20] García-Menor E, García-Marín F, Vecino-López R, et al. A Multicenter, Prospective, Randomized Controlled Trial to Evaluate the Additional Benefit of a Multistrain Synbiotic (Prodefen®) in the Clinical Management of Acute Viral Diarrhea in Children. Global pediatric health, 2016;3:2333794x16679587.

[21] Dash DK, Dash M, Mohanty MD, Acharya N. Efficacy of probiotic Saccharomyces boulardii as an adjuvant therapy in acute childhood diarrhoea. Journal of nepal paediatric society, 2016;36(3):250-255.

[22] Das S, Gupta PK, Das RR. Efficacy and Safety of Saccharomyces boulardii in Acute Rotavirus Diarrhea: double Blind Randomized Controlled Trial from a Developing Country. Journal of tropical pediatrics, 2016;62(6):464-470.

[23] Lee DK, Park JE, Kim MJ, Seo JG, Lee JH, Ha NJ. Probiotic bacteria, B. longum and L. acidophilus inhibit infection by rotavirus in vitro and decrease the duration of diarrhea in pediatric patients. Clinics and research in hepatology and gastroenterology, 2015;39(2):237-244.

[24] Hegar B, Waspada IM, Gunardi H, Vandenplas Y. A double blind randomized trial showing probiotics to be ineffective in acute diarrhea in Indonesian children. Indian journal of pediatrics, 2015;82(5):410-414.

[25] Freedman SB, Sherman PM, Willan A, Johnson D, Gouin S, Schuh S. Emergency Department Treatment of Children With Diarrhea Who Attend Day Care: a Randomized Multidose Trial of a Lactobacillus helveticus and Lactobacillus rhamnosus Combination Probiotic. Clinical pediatrics, 2015;54(12):1158-1166.

[26] El-Soud NHA, Said RN, Mosallam DS, Barakat NAM, Sabry MA. Bifidobacterium lactis in treatment of children with acute diarrhea. A randomized double blind controlled trial. Macedonian journal of medical sciences, 2015;3(3):403-407.

[27] Dinleyici EC, Kara A, Dalgic N, et al. Saccharomyces boulardii CNCM I-745 reduces the duration of diarrhoea, length of emergency care and hospital stay in children with acute diarrhoea. Beneficial microbes, 2015;6(4):415-421.

[28] Dinleyici EC, Dalgic N, Guven S, et al. Lactobacillus reuteri DSM 17938 shortens acute infectious diarrhea in a pediatric outpatient setting. Jornal de pediatria, 2015;91(4):392-396.

[29] Sindhu KN, Sowmyanarayanan TV, Paul A, et al. Immune response and intestinal permeability in children with acute gastroenteritis treated with Lactobacillus rhamnosus GG: a randomized, double-blind, placebo-controlled trial. Clinical infectious diseases, 2014;58(8):1107-1115.

[30] Dinleyici EC, Vandenplas Y. Lactobacillus reuteri DSM 17938 effectively reduces the duration of acute diarrhoea in hospitalised children. Acta paediatrica, 2014;103(7):e300-5.

[31] Huang YF, Liu PY, Chen YY, et al. Three-combination probiotics therapy in children with salmonella and rotavirus gastroenteritis. Journal of clinical gastroenterology, 2014;48(1):37-42.

[32] Azim K, Sheikh TS, Khan SN. Efficacy of probiotics (Saccharomyces bulardii) in acute watery diarrhoea in children. Journal of Rawalpindi Medical College, 2014;18(2):213-215.

[33] Aggarwal S, Upadhyay A, Shah D, Teotia N, Agarwal A, Jaiswal V. Lactobacillus GG for treatment of acute childhood diarrhoea: an open labelled, randomized controlled trial. Indian journal of medical research, 2014;139(3):379-385.

[34] Phavichitr N, Puwdee P, Tantibhaedhyangkul R. Cost-benefit analysis of the probiotic treatment of children hospitalized for acute diarrhea in Bangkok, Thailand. Southeast Asian journal of tropical medicine and public health, 2013;44(6):1065-1071.

[35] Dinleyici EC, Dalgic N, Guven S, et al. The effect of a multispecies synbiotic mixture on the duration of diarrhea and length of hospital stay in children with acute diarrhea in Turkey: single blinded randomized study. European journal of pediatrics, 2013;172(4):459-464.

[36] Burande M. Comparison of efficacy of Saccharomyces boulardii strain in the treatment of acute diarrhea in children: a prospective, single-blind, randomized controlled clinical trial. Journal of pharmacology & pharmacotherapeutics, 2013;4(3):205-208.

[37] Riaz M, Alam S, Malik A, Ali SM. Efficacy and safety of Saccharomyces boulardii in acute childhood diarrhea: a double blind randomised controlled trial. Indian journal of pediatrics, 2012;79(4):478-482.

[38] Nixon AF, Cunningham SJ, Cohen HW, Crain EF. The effect of Lactobacillus GG on acute diarrheal illness in the pediatric emergency department. Pediatric emergency care, 2012;28(10):1048-1051.

[39] Khan A, Javed T, Chishti AL. Clinical efficacy of use of probiotic "Saccharomyces boulardii" In children with acute watery diarrhea. Pakistan paediatric journal, 2012;36(3):122-127.

[40] Francavilla R, Lionetti E, Castellaneta S, et al. Randomised clinical trial: Lactobacillus reuteri DSM 17938 vs. placebo in children with acute diarrhoea - A double-blind study. Alimentary Pharmacology and Therapeutics, 2012;36(4):363-369.

[41] Erdoğan O, Tanyeri B, Torun E, et al. The comparition of the efficacy of two different probiotics in rotavirus gastroenteritis in children. Journal of tropical medicine, 2012;2012:787240.

[42] Vandenplas Y, De Hert SG. Randomised clinical trial: the synbiotic food supplement Probiotical vs. placebo for acute gastroenteritis in children. Alimentary pharmacology & therapeutics, 2011;34(8):862-867.

[43] Dutta P, Mitra U, Dutta S, Rajendran K, Saha TK, Chatterjee MK. Randomised controlled clinical trial of Lactobacillus sporogenes (Bacillus coagulans), used as probiotic in clinical practice, on acute watery diarrhoea in children. Tropical medicine & international health, 2011;16(5):555-561.

[44] Dalgic N, Sancar M, Bayraktar B, Pullu M, Hasim O. Probiotic, zinc and lactose-free formula in children with rotavirus diarrhea: are they effective? Pediatrics international, 2011;53(5):677-682.

[45] Corrêa NB, Penna FJ, Lima FM, Nicoli JR, Filho LA. Treatment of acute diarrhea with Saccharomyces boulardii in infants. Journal of pediatric gastroenterology and nutrition, 2011;53(5):497-501.

[46] Ritchie BK, Brewster DR, Tran CD, Davidson GP, McNeil Y, Butler RN. Efficacy of Lactobacillus GG in aboriginal children with acute diarrhoeal disease: a randomised clinical trial. Journal of pediatric gastroenterology and nutrition, 2010;50(6):619-624.

[47] Rerksuppaphol S, Rerksuppaphol L. Lactobacillus acidophilus and Bifidobacterium bifidum stored at ambient temperature are effective in the treatment of acute diarrhoea. Annals of tropical paediatrics, 2010;30(4):299-304.

[48] Grandy G, Medina M, Soria R, Terán CG, Araya M. Probiotics in the treatment of acute rotavirus diarrhoea. A randomized, double-blind, controlled trial using two different probiotic preparations in Bolivian children. BMC infectious diseases, 2010;10:253.

[49] Chen CC, Kong MS, Lai MW, et al. Probiotics have clinical, microbiologic, and immunologic efficacy in acute infectious diarrhea. Pediatric infectious disease journal, 2010;29(2):135-138.

[50] Misra S, Sabui TK, Pal NK. A randomized controlled trial to evaluate the efficacy of lactobacillus GG in infantile diarrhea. Journal of pediatrics, 2009;155(1):129-132.

[51] Teran CG, Teran-Escalera CN, Villarroel P. Nitazoxanide vs. probiotics for the treatment of acute rotavirus diarrhea in children: a randomized, single-blind, controlled trial in Bolivian children. International journal of infectious diseases, 2009;13(4):518-523.

[52] Kianifar HR, Farid R, Ahanchian H, Jabbari F, Moghiman T, Sistanian A. Probiotics in the treatment of acute diarrhea in young children. Iranian journal of medical sciences, 2009;34(3):204-207.

[53] Basu S, Paul DK, Ganguly S, Chatterjee M, Chandra PK. Efficacy of high-dose Lactobacillus rhamnosus GG in controlling acute watery diarrhea in Indian children: a randomized controlled trial. Journal of clinical gastroenterology, 2009;43(3):208-213.

[54] Rafeey M, Ostadrahimi A, Boniadi M, Ghorashi Z, Alizadeh MM, Hadafey V. Lactobacillus acidophilus yogurt and supplement in children with acute diarrhea: a clinical trial. Research journal of medical sciences, 2008;2(1):13-18.

[55] Narayanappa D. Randomized double blinded controlled trial to evaluate the efficacy and safety of Bifilac in patients with acute viral diarrhea. Indian journal of pediatrics, 2008;75(7):709-713.

[56] Mao M, Yu T, Xiong Y, et al. Effect of a lactose-free milk formula supplemented with bifidobacteria and streptococci on the recovery from acute diarrhoea. Asia Pacific journal of clinical nutrition, 2008;17(1):30-34.

[57] Villarruel G, Rubio DM, Lopez F, et al. Saccharomyces boulardii in acute childhood diarrhoea: a randomized, placebo-controlled study. Acta paediatrica, 2007;96(4):538-541.

[58] Ozkan TB, Sahin E, Erdemir G, Budak F. Effect of Saccharomyces boulardii in children with acute gastroenteritis and its relationship to the immune response. Journal of international medical research, 2007;35(2):201-212.

[59] Canani RB, Cirillo P, Terrin G, et al. Probiotics for treatment of acute diarrhoea in children: randomised clinical trial of five different preparations. BMJ, 2007;335(7615):340.

[60] Henker J, Laass M, Blokhin BM, et al. The probiotic Escherichia coli strain Nissle 1917 (EcN) stops acute diarrhoea in infants and toddlers. European journal of pediatrics, 2007;166(4):311-318.

[61] Basu S, Chatterjee M, Ganguly S, Chandra PK. Efficacy of Lactobacillus rhamnosus GG in acute watery diarrhoea of Indian children: a randomised controlled trial. Journal of paediatrics and child health, 2007;43(12):837-842.

[62] Vivatvakin B, Kowitdamrong E. Randomized control trial of live Lactobacillus acidophilus plus Bifidobacterium infantis in treatment of infantile acute watery diarrhea. Chotmaihet thangphaet [Journal of the Medical Association of Thailand], 2006;89 Suppl 3:S126-33.

[63] Szymański H, Pejcz J, Jawień M, Chmielarczyk A, Strus M, Heczko PB. Treatment of acute infectious diarrhoea in infants and children with a mixture of three Lactobacillus rhamnosus strains - A randomized, double-blind, placebo-controlled trial. Alimentary Pharmacology and Therapeutics, 2006;23(2):247-253.

[64] Billoo AG, Memon MA, Khaskheli SA, et al. Role of a probiotic (Saccharomyces boulardii) in management and prevention of diarrhoea. World journal of gastroenterology, 2006;12(28):4557-4560.

[65] Sarker SA, Sultana S, Fuchs GJ, et al. Lactobacillus paracasei strain ST11 has no effect on rotavirus but ameliorates the outcome of nonrotavirus diarrhea in children from Bangladesh. Pediatrics,

2005;116(2):e221-8.

[66] Kurugöl Z, Koturoğlu G. Effects of Saccharomyces boulardii in children with acute diarrhoea. Acta paediatrica, 2005;94(1):44-47.

[67] Kowalska-Duplaga K, Fyderek K, Szajewska H, Janiak R. Efficacy of Trilac® in the treatment of acute diarrhoea in infants and young children - A multicentre, randomized, double-blind placebo-controlled study. Pediatria wspolczesna, 2004;6(3):295-299.

[68] Costa-Ribeiro H, Ribeiro TC, Mattos AP, et al. Limitations of probiotic therapy in acute, severe dehydrating diarrhea. Journal of pediatric gastroenterology and nutrition, 2003;36(1):112-115.

[69] Rosenfeldt V, Michaelsen KF, Jakobsen M, et al. Effect of probiotic Lactobacillus strains on acute diarrhea in a cohort of nonhospitalized children attending day-care centers. Pediatric infectious disease journal, 2002;21(5):417-419.

[70] Rosenfeldt V, Michaelsen KF, Jakobsen M, et al. Effect of probiotic Lactobacillus strains in young children hospitalized with acute diarrhea. Pediatric infectious disease journal, 2002;21(5):411-416.

[71] Hafeez A, Tariq P, Ali S, Kundi ZU, Khan A, Hassan M. The efficacy of Saccharomyces boulardii in the treatment of acute watery diarrhea in children: a multicentre randomized controlled trial. Journal of the College of Physicians and Surgeons--Pakistan : JCPSP, 2002;12(7):432-434.

[72] Urganci N, Polat T, Uysalol M, Cetinkaya F. Evaluation of the efficacy of Saccharomyces boulardii in children with acute diarrhoea. Archives of gastroenterohepatology, 2001;20(3-4):81-83.

[73] Lee MC, Lin LH, Hung KL, Wu HY. Oral bacterial therapy promotes recovery from acute diarrhea in children. Acta paediatrica Taiwanica, 2001;42(5):301-305.

[74] Boudraa G, Benbouabdellah M, Hachelaf W, Boisset M, Desjeux JF, Touhami M. Effect of feeding yogurt versus milk in children with acute diarrhea and carbohydrate malabsorption. Journal of pediatric gastroenterology and nutrition, 2001;33(3):307-313.

[75] Guandalini S, Pensabene L, Zikri MA, et al. Lactobacillus GG administered in oral rehydration solution to children with acute diarrhea: a multicenter European trial. Journal of pediatric gastroenterology and nutrition. 2000;30(1):54-60. doi:10.1097/00005176-200001000-00018

[76] Hernandez CL, Pineda EE, Jimenez MIR, Lucena MS. Clinical therapeutic affect of Saccharomyces boulardii on children with acute diarrhea. Revista de enfermedades infecciosas en pediatria, 1998;11(43):87-89.

[77] Shornikova AV, Isolauri E, Burkanova L, Lukovnikova S, Vesikari T. A trial in the Karelian Republic of oral rehydration and Lactobacillus GG for treatment of acute diarrhoea. Acta paediatrica, 1997;86(5):460-465.

[78] Shornikova AV, Casas IA, Mykkänen H, Salo E, Vesikari T. Bacteriotherapy with Lactobacillus reuteri in rotavirus gastroenteritis. Pediatric infectious disease journal, 1997;16(12):1103-1107.

[79] Shornikova AV, Casas IA, Isolauri E, Mykkänen H, Vesikari T. Lactobacillus reuteri as a therapeutic agent in acute diarrhea in young children. Journal of pediatric gastroenterology and nutrition, 1997;24(4):399-404.

[80] Guarino A, Canani RB, Spagnuolo MI, Albano F, Di Benedetto L. Oral bacterial therapy reduces the duration of symptoms and of viral excretion in children with mild diarrhea. Journal of pediatric gastroenterology and nutrition, 1997;25(5):516-519.

[81] Pant AR, Graham SM, Allen SJ, et al. Lactobacillus GG and acute diarrhoea in young children in the tropics. Journal of tropical pediatrics, 1996;42(3):162-165.

[82] Raza S, Graham SM, Allen SJ, Sultana S, Cuevas L, Hart CA. Lactobacillus GG promotes recovery

from acute nonbloody diarrhea in Pakistan. Pediatric infectious disease journal, 1995;14(2):107-111.

[83] Isolauri E, Kaila M, Mykkänen H, Ling WH, Salminen S. Oral bacteriotherapy for viral gastroenteritis. Digestive diseases and sciences, 1994;39(12):2595-2600.

[84] Cetina-Sauri G, Sierra Basto G. Evaluation of Saccharomyces boulardii for the treatment of acute diarrhea in pediatric patients. Annales de pediatrie, 1994;41(6):397-400.

Figure S1. Risk of bias graph

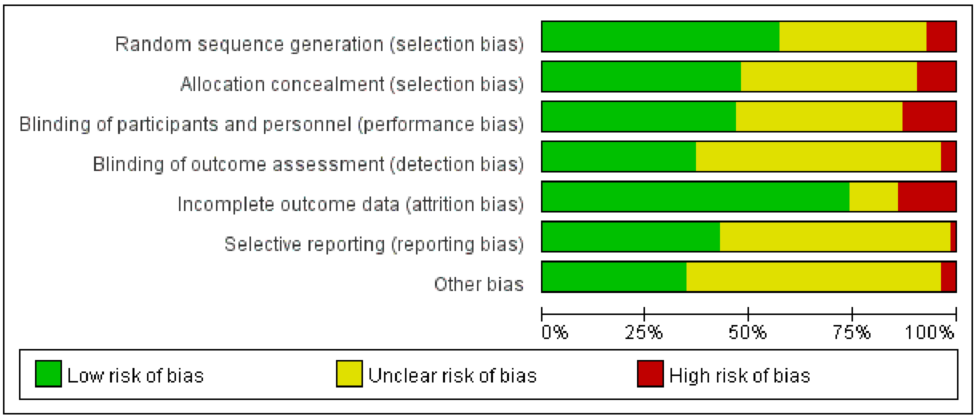

Figure S2. Risk of bias summary

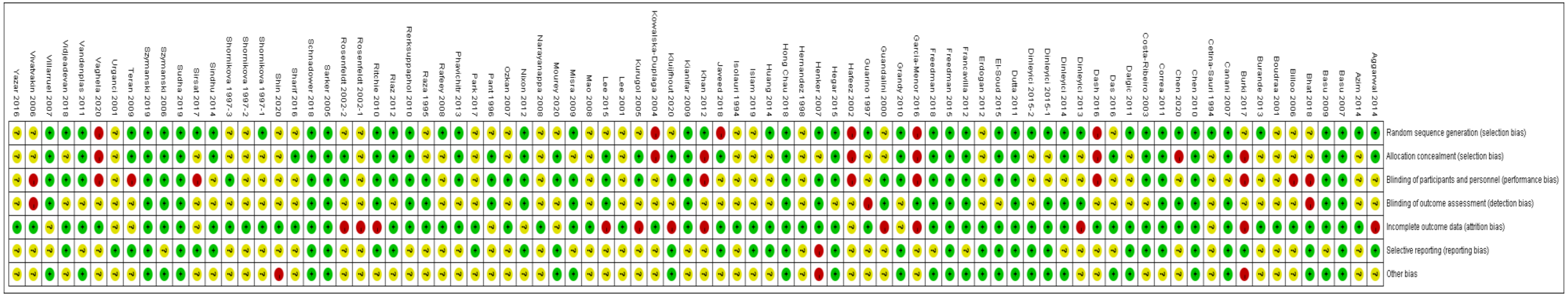

Figure S3. Incoherence plot for the duration of diarrhea (control = placebo/no treatment)

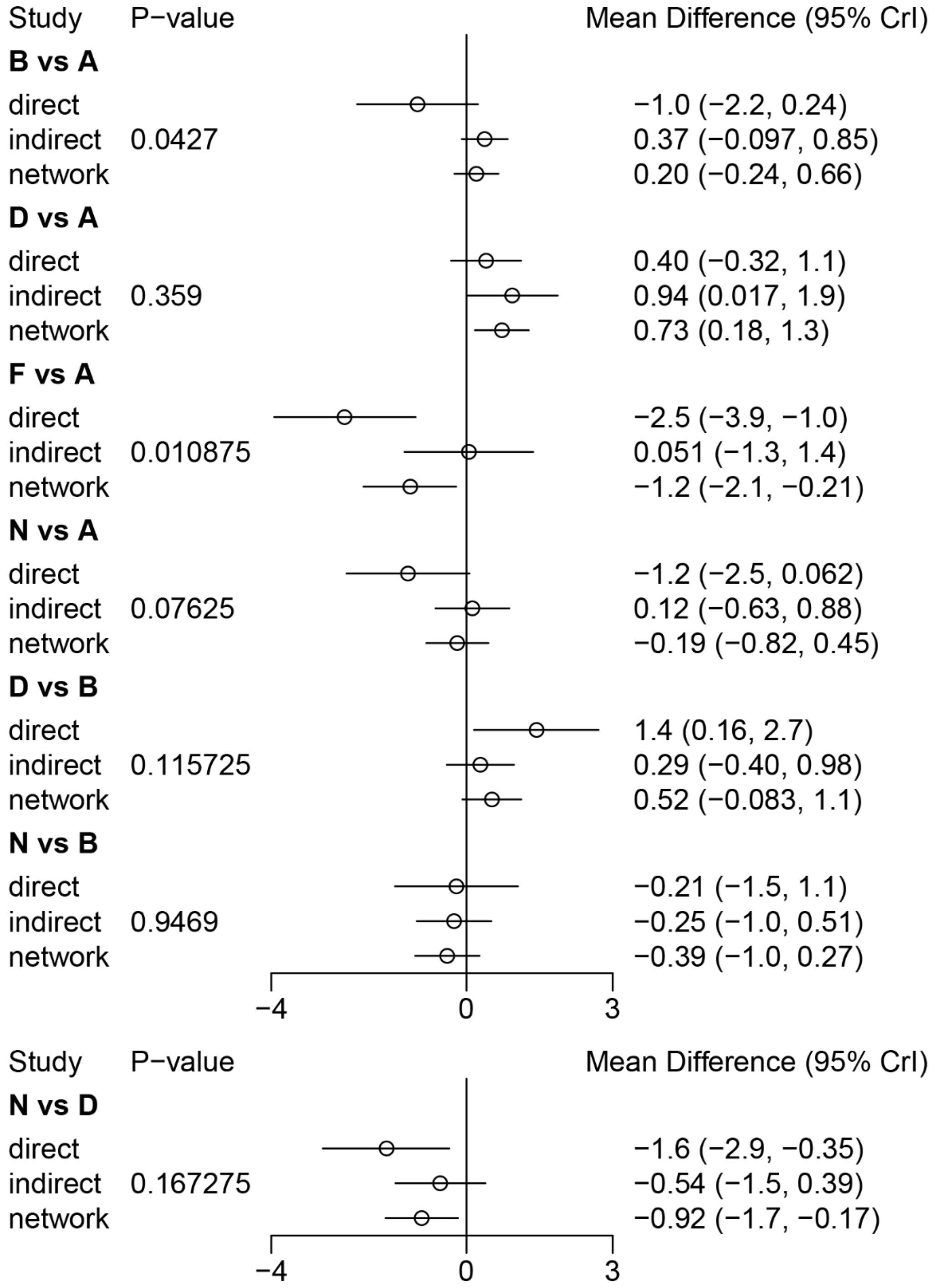

Footnote. **A**, *S. boulardii*; **B**, LGG; **D**, *Bacillus clausii*; **F**, *B. lactis*; **N**, *L.* species (spp) + *B.* spp + *S.* spp.

Figure S4. Incoherence plot for the duration of diarrhea (control = no treatment)

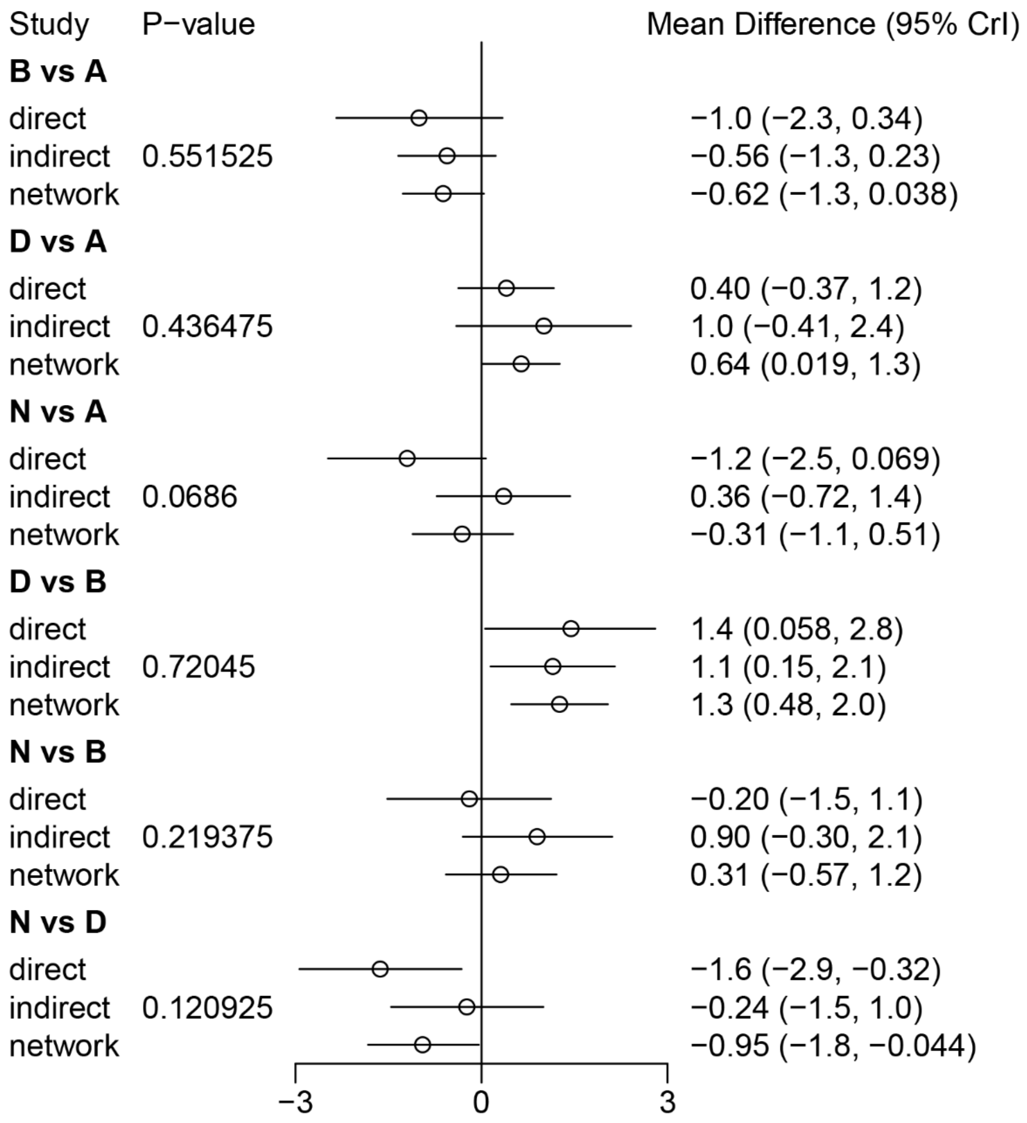

Footnote. **A**, *S. boulardii*; **B**, LGG; **D**, *Bacillus clausii*; **F**, *B. lactis*; **N**, *L.* spp + *B.* spp + *S.* spp.

Figure S5. Incoherence plot for the duration of hospitalization

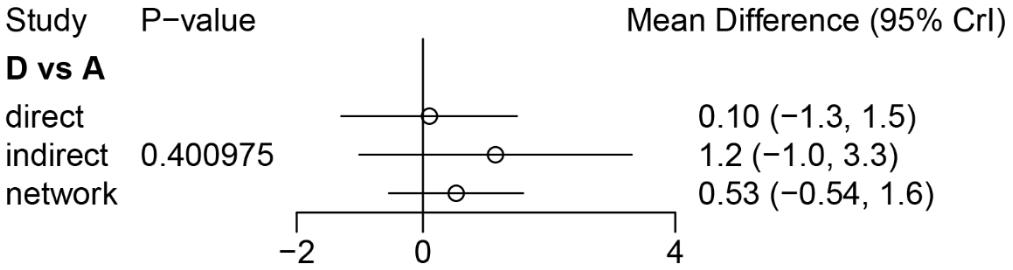

Footnote. **A**, *S. boulardii*; **D**, *Bacillus clausii.*

Figure S6. Incoherence plot for the mean stool frequency on day 2

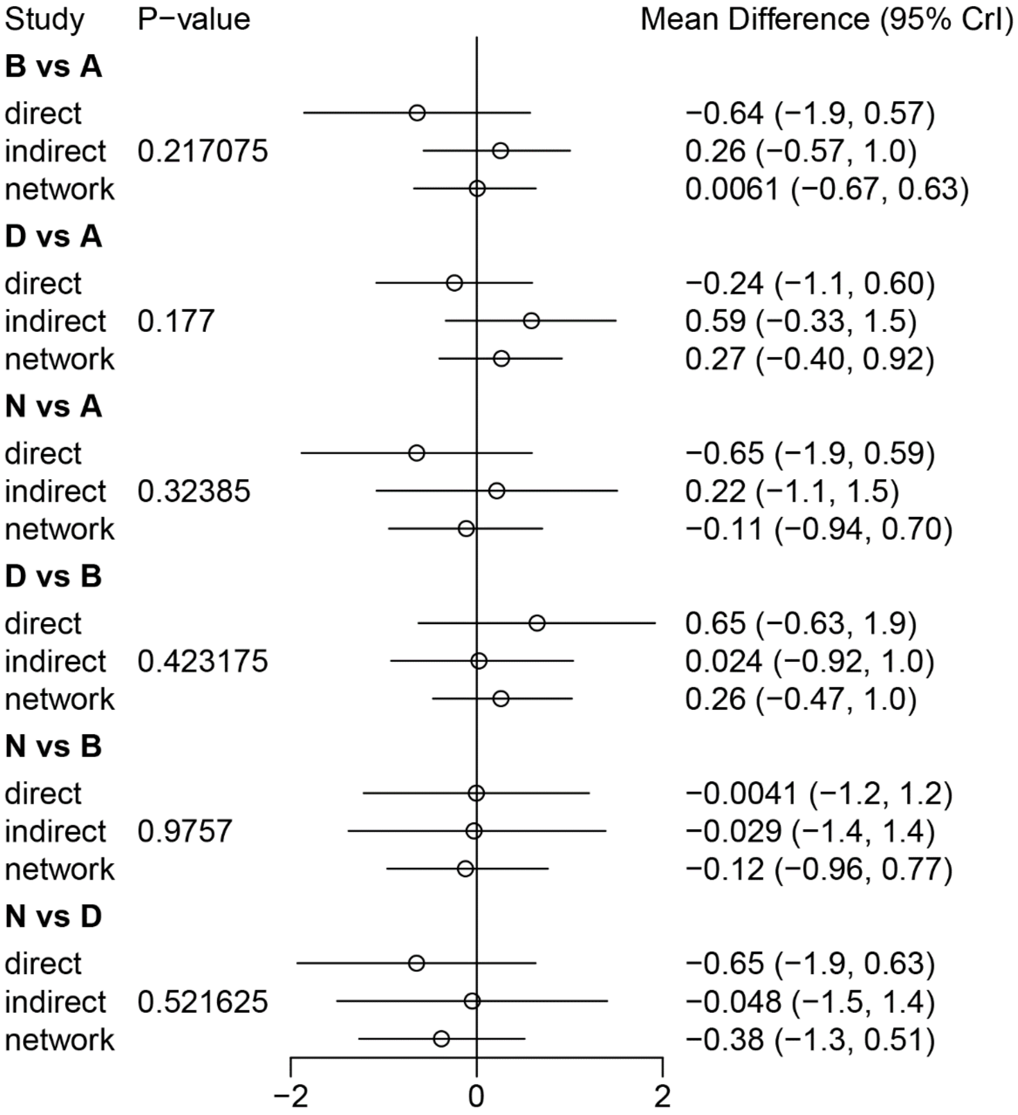

Footnote. **A**, *S. boulardii*; **B**, LGG; **D**, *Bacillus clausii*; **N**, *L*. spp + *B*. spp + *S*. spp.

Figure S7. Incoherence plot for the duration of vomiting

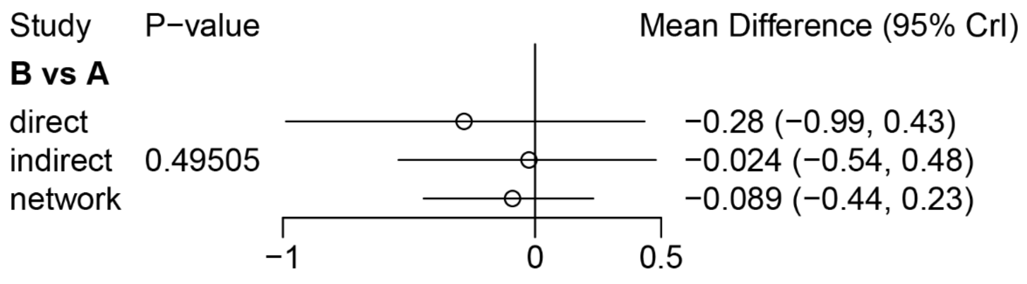

Footnote. **A**, *S. boulardii*; **B**, LGG.

Figure S8. Incoherence plot for the duration of fever

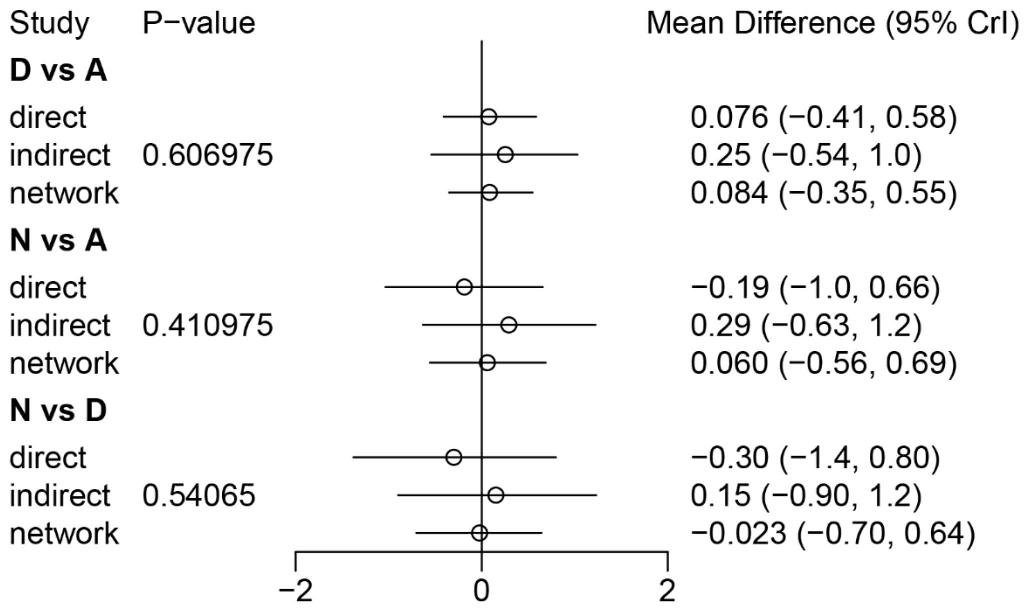

Footnote. **A**, *S. boulardii*; **D**, *Bacillus clausii*; **N**, *L*. spp + *B*. spp + *S*. spp.

Table S3. Heterogeneity for the duration of diarrhea (control = placebo/no treatment)

Comparison	I^2(pair−wise)	I^2(network)	P
A vs B	NA	97.4%	0.032
A vs D	0	76.7%	0.11
A vs F	NA	88.9%	0.024
A vs K	NA	NA	NA
A vs N	NA	95.8%	0.053
A vs U	87.6%	88.4%	NA
B vs D	NA	80.0%	0.15
B vs K	NA	NA	NA
B vs N	NA	0	0.81
B vs U	98.1%	98.3%	NA
C vs U	49.9%	49.9%	NA
D vs K	NA	NA	NA
D vs N	NA	62.8%	0.26
D vs U	55.1%	54.7%	NA
E vs U	NA	NA	NA
F vs U	93.1%	95.4%	NA
G vs U	NA	NA	NA
H vs U	NA	NA	NA
I vs U	NA	NA	NA
J vs U	NA	NA	NA
K vs N	NA	NA	NA
K vs U	NA	NA	NA
L vs U	47.4%	48.1%	NA
M vs U	64.0%	64.0%	NA
N vs U	3.2%	14.2%	NA
O vs U	NA	NA	NA
P vs U	NA	NA	NA
Q vs U	0	0	NA
R vs U	NA	NA	NA
S vs U	NA	NA	NA

T vs U	NA	NA	NA

Footnote. **A**, *S. boulardii*; **B**, LGG; **C**, *L. reuteri*; **D**, *Bacillus clausii*; **E**, *L. acidophilus*; **F**, *B. lactis*; **G**, *L. sporogenes*; **H**, *L. plantarum*; **I**, *ECN 1917*; **J**, *L. paracasei*; **K**, *E. faecium*; **L**, *L.* spp; **M**, *L.* spp + *B.* spp; **N**, *L.* spp + *B.* spp+ *S.* spp; **O**, *L.* spp + *S.* spp; **P**, *B.* spp + *S.* spp; **Q**, *Bacillus* spp + *E.* spp + C. spp; **R**, *L.* spp + *B.* spp + *E.* spp; **S,** L. spp + B. spp + *P.* spp; **T**, *L.* spp + *S.* spp + *C.* spp + *Bacillus* spp; **U**, Control (placebo/no treatment).

Table S4. Heterogeneity for the duration of diarrhea (control = placebo)

Comparison	I^2(pair−wise)	I^2(network)	P
A vs U	73.9%	73.8%	NA
B vs U	83.6%	83.6%	NA
C vs U	0	0	NA
D vs U	NA	NA	NA
E vs U	NA	NA	NA
F vs U	NA	NA	NA
G vs U	NA	NA	NA
I vs U	NA	NA	NA
J vs U	NA	NA	NA
L vs U	42.9%	42.9%	NA
M vs U	70.5%	70.5%	NA
N vs U	0	0	NA
P vs U	NA	NA	NA
Q vs U	NA	NA	NA
S vs U	NA	NA	NA
T vs U	NA	NA	NA

Footnote. **A**, *S. boulardii*; **B**, LGG; **C**, *L. reuteri*; **D**, *Bacillus clausii*; **E**, *L. acidophilus*; **F**, *B. lactis*; **G**, *L. sporogenes*; **I**, *ECN 1917*; **J**, *L. paracasei*; **L**, *L.* spp; **M**, *L.* spp + *B.* spp; **N**, *L.* spp + *B.* spp+ *S.* spp; **P**, *B.* spp + *S.* spp; **Q**, *Bacillus* spp + *E.* spp + C. spp; **S,** L. spp + B. spp + *P.* spp; **T**, *L.* spp + *S.* spp + *C.* spp + *Bacillus* spp; **U**, Control (placebo).

Table S5. Heterogeneity for the duration of diarrhea (control = no treatment)

**Comparison**	**I^2(pair−wise)**	**I^2(network)**	**P**
A vs B	NA	75.7%	0.47
A vs D	0	58.7%	0.17
A vs F	NA	NA	NA
A vs K	NA	NA	NA
A vs N	NA	94.7%	0.067
A vs U	89.1%	88.7%	NA
B vs D	NA	0	0.76
B vs K	NA	NA	NA
B vs N	NA	0	0.53
B vs U	97.5%	97.5%	NA
C vs U	86.1%	86.1%	NA
D vs K	NA	NA	NA
D vs N	NA	61.2%	0.26
D vs U	66.5%	68.0%	NA
F vs U	NA	NA	NA
H vs U	NA	NA	NA
K vs N	NA	NA	NA
K vs U	NA	NA	NA
M vs U	65.0%	65.3%	NA
N vs U	32.8%	65.8%	NA
O vs U	NA	NA	NA
Q vs U	NA	NA	NA
R vs U	NA	NA	NA

Footnote. **A**, *S. boulardii*; **B**, LGG; **C**, *L. reuteri*; **D**, *Bacillus clausii*; **F**, *B. lactis*; **H**, *L. plantarum*; **K**, *E. faecium*; **M**, *L.* spp + *B.* spp; **N**, *L.* spp + *B.* spp+ *S.* spp; **O**, *L.* spp + *S.* spp; **Q**, *Bacillus* spp + *E.* spp + C. spp; **R**, *L.* spp + *B.* spp + *E.* spp; **U**, Control (no treatment).

Table S6. Heterogeneity for diarrhea lasting ≥2 days

Comparison	I^2(pair−wise)	I^2(network)	P
A vs U	91.6%	91.6%	NA
B vs U	81.9%	82.0%	NA
C vs U	67.4%	67.4%	NA
D vs U	NA	NA	NA
E vs U	NA	NA	NA
I vs U	NA	NA	NA
L vs U	NA	NA	NA
M vs U	0	0	NA
N vs U	46.0%	46.7%	NA
O vs U	NA	NA	NA
R vs U	NA	NA	NA

Footnote. **A**, *S. boulardii*; **B**, LGG; **C**, *L. reuteri*; **D**, *Bacillus clausii*; **E**, *L. acidophilus*; **I**, *ECN 1917*; **L**, *L.* spp; **M**, *L.* spp + *B.* spp; **N**, *L.* spp + *B.* spp+ *S.* spp; **O**, *L.* spp + *S.* spp; **P**, *B.* spp + *S.* spp; **R**, *L.* spp + *B.* spp + *E.* spp; **U**, Control (placebo/no treatment).

Table S7. Heterogeneity for the duration of hospitalization

Comparison	I^2(pair−wise)	I^2(network)	P
A vs D	92.3%	82.1%	0.26
A vs U	91.4%	91.6%	NA
B vs U	99.0%	99.0%	NA
C vs U	85.2%	85.2%	NA
D vs U	0	72.0%	NA
E vs U	79.8%	80.0%	NA
F vs U	NA	NA	NA
H vs U	NA	NA	NA
L vs U	NA	NA	NA
M vs U	62.2%	62.2%	NA
N vs U	NA	NA	NA
Q vs U	96.3%	96.3%	NA
R vs U	NA	NA	NA

Footnote. **A**, *S. boulardii*; **B**, LGG; **C**, *L. reuteri*; **D**, *Bacillus clausii*; **E**, *L. acidophilus*; **H**, *L. plantarum*; **L**, *L.* spp; **M**, *L.* spp + *B.* spp; **N**, *L.* spp + *B.* spp+ *S.* spp; **Q**, *Bacillus* spp + *E.* spp + C. spp; **R**, *L.* spp + *B.* spp + *E.* spp; **U**, Control (placebo/no treatment).

Table S8. Heterogeneity for the mean stool frequency on day 2

Comparison	I^2(pair−wise)	I^2(network)	P
A vs B	NA	59.7%	0.25
A vs D	0	62.0%	0.11
A vs K	NA	NA	NA
A vs N	NA	46.0%	0.30
A vs U	84.5%	87.6%	NA
B vs D	NA	2.22%	0.46
B vs K	NA	NA	NA
B vs N	NA	0	0.75
B vs U	49.2%	62.9%	NA
C vs U	49.0%	49.2%	NA
D vs K	NA	NA	NA
D vs N	NA	0	0.57
E vs U	NA	NA	NA
K vs N	NA	NA	NA
K vs U	NA	NA	NA
L vs U	98.9%	99.2%	NA
M vs U	80.5%	80.7%	NA
N vs U	0	0	NA
Q vs U	NA	NA	NA
R vs U	NA	NA	NA
T vs U	NA	NA	NA

Footnote. **A**, *S. boulardii*; **B**, LGG; **C**, *L. reuteri*; **D**, *Bacillus clausii*; **E**, *L. acidophilus*; **K**, *E. faecium*; **L**, *L.* spp; **M**, *L.* spp + *B.* spp; **N**, *L.* spp + *B.* spp+ *S.* spp; **Q**, *Bacillus* spp + *E.* spp + C. spp; **R**, *L.* spp + *B.* spp + *E.* spp; **T**, *L.* spp + *S.* spp + *C.* spp + *Bacillus* spp; **U**, Control (placebo/no treatment).

Table S9. Heterogeneity for the duration of vomiting

Comparison	I^2(pair−wise)	I^2(network)	P
A vs B	NA	0	0.66
A vs D	NA	NA	NA
A vs K	NA	NA	NA
A vs N	NA	NA	NA
A vs U	83.7%	84.3%	NA
B vs D	NA	NA	NA
B vs K	NA	NA	NA
B vs N	NA	NA	NA
B vs U	0	0	NA
D vs K	NA	NA	NA
D vs N	NA	NA	NA
D vs U	NA	NA	NA
H vs U	NA	NA	NA
K vs N	NA	NA	NA
K vs U	NA	NA	NA
L vs U	0	0	NA
N vs U	NA	NA	NA
Q vs U	NA	NA	NA
S vs U	NA	NA	NA

Footnote. **A**, *S. boulardii*; **B**, LGG; **D**, *Bacillus clausii*; **H**, *L. plantarum*; **K**, *E. faecium*; **L**, *L.* spp; **N**, *L.* spp + *B.* spp+ *S.* spp; **Q**, *Bacillus* spp + *E.* spp + C. spp; **S,** L. spp + B. spp + *P*. spp; **U**, Control (placebo/no treatment).

Table S10. Heterogeneity for the duration of fever

Comparison	I^2(pair−wise)	I^2(network)	P
A vs B	NA	NA	NA
A vs D	0	0	0.98
A vs K	NA	NA	NA
A vs N	NA	0	0.47
A vs U	83.6%	83.9%	NA
B vs D	NA	NA	NA
B vs K	NA	NA	NA
B vs N	NA	NA	NA
B vs U	NA	NA	NA
D vs K	NA	NA	NA
D vs N	NA	0	0.91
D vs U	47.1%	34.0%	NA
F vs U	NA	NA	NA
H vs U	NA	NA	NA
K vs N	NA	NA	NA
K vs U	NA	NA	NA
L vs U	NA	NA	NA
M vs U	NA	NA	NA
N vs U	0	0	NA
Q vs U	76.6%	76.5%	NA
S vs U	NA	NA	NA

Footnote. **A**, *S. boulardii*; **B**, LGG; **D**, *Bacillus clausii*; **F**, *B. lactis*; **H**, *L. plantarum*; **K**, *E. faecium*; **L**, *L.* spp; **M**, *L.* spp + *B.* spp; **N**, *L.* spp + *B.* spp+ *S.* spp; **Q**, *Bacillus* spp + *E.* spp + C. spp; **S,** L. spp + B. spp + *P.* spp; **U**, Control (placebo/no treatment).

Table S11. NMA results for the duration of diarrhea (control = placebo/no treatment)

**A**	**A**																					
**B**	1.0 (-0.24, 2.2)	**B**																				
**C**	-0.07 (-0.73, 0.59)	0.13 (-0.55, 0.81)	**C**																			
**D**	-0.73 (-1.28, -0.17)	-0.52 (-1.13, 0.08)	-0.65 (-1.44, 0.12)	**D**																		
**E**	-1.01 (-2.34, 0.31)	-0.81 (-2.15, 0.53)	-0.94 (-2.36, 0.48)	-0.29 (-1.68, 1.1)	**E**																	
**F**	2.5 (1.0, 3.9)	1.35 (0.37, 2.34)	1.22 (0.14, 2.32)	1.88 (0.83, 2.93)	2.16 (0.58, 3.76)	**F**																
**G**	-0.89 (-2.19, 0.41)	-0.68 (-2, 0.63)	-0.81 (-2.21, 0.58)	-0.16 (-1.53, 1.21)	0.13 (-1.68, 1.94)	-2.04 (-3.61, -0.48)	**G**															
**H**	0.24 (-1.08, 1.57)	0.45 (-0.89, 1.78)	0.32 (-1.1, 1.73)	0.97 (-0.42, 2.36)	1.26 (-0.57, 3.08)	-0.91 (-2.5, 0.68)	1.13 (-0.67, 2.94)	**H**														
**I**	0.45 (-0.85, 1.75)	0.65 (-0.65, 1.96)	0.53 (-0.87, 1.92)	1.18 (-0.19, 2.55)	1.46 (-0.34, 3.28)	-0.7 (-2.27, 0.86)	1.34 (-0.45, 3.12)	0.21 (-1.6, 2.02)	**I**													
**J**	-0.82 (-2.18, 0.53)	-0.62 (-1.99, 0.74)	-0.75 (-2.19, 0.7)	-0.1 (-1.52, 1.32)	0.19 (-1.66, 2.04)	-1.97 (-3.59, -0.37)	0.06 (-1.77, 1.9)	-1.07 (-2.92, 0.78)	-1.28 (-3.1, 0.55)	**J**												

**K**	-1.14 (-2.21, -0.08)	-0.94 (-2.01, 0.13)	-1.07 (-2.27, 0.12)	-0.42 (-1.53, 0.69)	-0.13 (-1.79, 1.54)	-2.29 (-3.69, -0.91)	-0.26 (-1.9, 1.38)	-1.39 (-3.05, 0.27)	-1.59 (-3.24, 0.04)	-0.32 (-2, 1.36)	**K**										
**L**	-0.68 (-1.37, 0.02)	-0.47 (-1.18, 0.24)	-0.61 (-1.46, 0.25)	0.05 (-0.76, 0.86)	0.34 (-1.09, 1.78)	-1.83 (-2.94, -0.71)	0.21 (-1.2, 1.62)	-0.92 (-2.35, 0.52)	-1.13 (-2.54, 0.28)	0.14 (-1.31, 1.61)	0.47 (-0.75, 1.69)	**L**									
**M**	-0.12 (-0.68, 0.43)	0.08 (-0.49, 0.66)	-0.05 (-0.8, 0.7)	0.6 (-0.09, 1.3)	0.89 (-0.48, 2.27)	-1.27 (-2.31, -0.24)	0.76 (-0.58, 2.12)	-0.36 (-1.73, 1.01)	-0.57 (-1.92, 0.78)	0.7 (-0.69, 2.11)	1.02 (-0.12, 2.17)	0.56 (-0.23, 1.33)	**M**								
**N**	0.19 (-0.46, 0.82)	0.39 (-0.27, 1.05)	0.26 (-0.56, 1.08)	0.91 (0.17, 1.66)	1.2 (-0.21, 2.61)	-0.96 (-2.06, 0.12)	1.07 (-0.32, 2.47)	-0.06 (-1.47, 1.35)	-0.26 (-1.66, 1.12)	1.01 (-0.44, 2.45)	1.33 (0.2, 2.47)	0.87 (0.01, 1.71)	0.31 (-0.44, 1.05)	**N**							
**O**	-0.26 (-1.64, 1.12)	-0.05 (-1.44, 1.33)	-0.18 (-1.65, 1.28)	0.47 (-0.97, 1.91)	0.76 (-1.1, 2.62)	-1.41 (-3.05, 0.22)	0.63 (-1.21, 2.47)	-0.5 (-2.36, 1.36)	-0.71 (-2.55, 1.13)	0.57 (-1.32, 2.44)	0.89 (-0.81, 2.59)	0.42 (-1.07, 1.9)	-0.13 (-1.56, 1.29)	-0.44 (-1.91, 1.02)	**O**						
**P**	-0.95 (-2.22, 0.33)	-0.74 (-2.02, 0.54)	-0.87 (-2.24, 0.5)	-0.22 (-1.56, 1.12)	0.07 (-1.72, 1.85)	-2.1 (-3.64, -0.56)	-0.06 (-1.83, 1.72)	-1.19 (-2.98, 0.59)	-1.4 (-3.16, 0.37)	-0.12 (-1.92, 1.69)	0.2 (-1.41, 1.81)	-0.27 (-1.65, 1.11)	-0.82 (-2.15, 0.5)	-1.13 (-2.5, 0.24)	-0.69 (-2.51, 1.14)	**P**					
**Q**	0.12 (-0.85, 1.08)	0.32 (-0.66, 1.3)	0.19 (-0.9, 1.28)	0.84 (-0.21, 1.89)	1.13 (-0.46, 2.72)	-1.04 (-2.34, 0.26)	1 (-0.56, 2.57)	-0.13 (-1.71, 1.45)	-0.34 (-1.9, 1.23)	0.94 (-0.67, 2.54)	1.26 (-0.13, 2.65)	0.79 (-0.32, 1.9)	0.24 (-0.79, 1.26)	-0.07 (-1.16, 1.02)	0.37 (-1.25, 2)	1.06 (-0.48, 2.6)	**Q**				
**R**	0.55 (-0.78, 1.87)	0.75 (-0.59, 2.09)	0.62 (-0.8, 2.04)	1.27 (-0.12, 2.66)	1.56 (-0.27, 3.39)	-0.6 (-2.2, 0.98)	1.43 (-0.38, 3.24)	0.3 (-1.52, 2.12)	0.1 (-1.71, 1.9)	1.37 (-0.48, 3.21)	1.69 (0.03, 3.35)	1.23 (-0.22, 2.65)	0.67 (-0.71, 2.04)	0.36 (-1.06, 1.77)	0.8 (-1.06, 2.67)	1.49 (-0.3, 3.28)	0.43 (-1.15, 2.02)	**R**			
**S**	0.11 (-1.48, 1.71)	0.32 (-1.29, 1.92)	0.19 (-1.48, 1.86)	0.84 (-0.81, 2.49)	1.13 (-0.91, 3.16)	-1.04 (-2.86, 0.78)	1 (-1.02, 3.01)	-0.13 (-2.16, 1.9)	-0.34 (-2.35, 1.67)	0.94 (-1.1, 2.98)	1.26 (-0.63, 3.14)	0.79 (-0.89, 2.47)	0.24 (-1.4, 1.87)	-0.07 (-1.74, 1.6)	0.37 (-1.69, 2.43)	1.06 (-0.93, 3.05)	0 (-1.82, 1.81)	-0.43 (-2.47, 1.6)	**S**		
**T**	0.11 (-1.31, 1.54)	0.32 (-1.11, 1.75)	0.19 (-1.32, 1.7)	0.84 (-0.64, 2.33)	1.13 (-0.77, 3.03)	-1.03 (-2.71, 0.63)	1 (-0.88, 2.88)	-0.13 (-2.03, 1.78)	-0.34 (-2.21, 1.54)	0.94 (-0.98, 2.86)	1.26 (-0.48, 3)	0.79 (-0.73, 2.32)	0.24 (-1.23, 1.71)	-0.07 (-1.58, 1.44)	0.37 (-1.56, 2.31)	1.06 (-0.8, 2.92)	0 (-1.67, 1.67)	-0.43 (-2.32, 1.47)	0 (-2.09, 2.1)	**T**	

**U**	-0.99 (-1.29, -0.68)	-0.78 (-1.12, -0.44)	-0.91 (-1.5, -0.32)	-0.26 (-0.78, 0.26)	0.03 (-1.27, 1.32)	-2.13 (-3.06, -1.22)	-0.1 (-1.36, 1.17)	-1.23 (-2.52, 0.06)	-1.44 (-2.7, -0.17)	-0.16 (-1.48, 1.16)	0.16 (-0.88, 1.2)	-0.31 (-0.93, 0.31)	-0.86 (-1.33, -0.4)	-1.17 (-1.75, -0.59)	-0.73 (-2.07, 0.61)	-0.04 (-1.28, 1.2)	-1.1 (-2.02, -0.18)	-1.53 (-2.82, -0.24)	-1.1 (-2.66, 0.47)	-1.1 (-2.49, 0.29)	**U**

Footnote. Results were mean change [95% confidence interval (CrI)] from the network meta-analysis. Mean difference values less than 0 indicates that the listed intervention was better than the row intervention. **A**, *S. boulardii*; **B**, LGG; **C**, *L. reuteri*; **D**, *Bacillus clausii*; **E**, *L. acidophilus*; **F**, *B. lactis*; **G**, *L. sporogenes*; **H**, *L. plantarum*; **I**, *ECN 1917*; **J**, *L. paracasei*; **K**, *E. faecium*; **L**, *L.* spp; **M**, *L.* spp + *B.* spp; **N**, *L.* spp + *B.* spp+ *S.* spp; **O**, *L.* spp + *S.* spp; **P**, *B.* spp + *S.* spp; **Q**, *Bacillus* spp + *E.* spp + C. spp; **R**, *L.* spp + *B.* spp + *E.* spp; **S,** L. spp + B. spp + *P.* spp; **T**, *L.* spp + *S.* spp + *C.* spp + *Bacillus* spp; **U**, Control (placebo/no treatment).

Table S12. NMA results for the duration of diarrhea (control = placebo)

**A**	**A**																
**B**	-1.02(-1.44, -0.57)	**B**															
**C**	-0.41(-1.05, 0.23)	0.61(-0.01, 1.21)	**C**														
**D**	-0.99(-1.76, -0.22)	0.03(-0.73, 0.76)	-0.58(-1.47, 0.3)	**D**													
**E**	-1.28(-2.12, -0.44)	-0.26(-1.09, 0.54)	-0.87(-1.82, 0.07)	-0.29(-1.33, 0.75)	**E**												
**F**	-0.27(-1.18, 0.63)	0.75(-0.15, 1.62)	0.14(-0.87, 1.14)	0.72(-0.37, 1.81)	1.01(-0.13, 2.15)	**F**											
**G**	-1.15(-1.95, -0.35)	-0.13(-0.92, 0.62)	-0.74(-1.65, 0.16)	-0.16(-1.16, 0.84)	0.13(-0.93, 1.19)	-0.88(-1.98, 0.23)	**G**										
**I**	0.19(-0.61, 0.98)	1.21(0.43, 1.96)	0.6(-0.3, 1.5)	1.18(0.18, 2.18)	1.47(0.42, 2.52)	0.46(-0.64, 1.57)	1.34(0.32, 2.36)	**I**									
**J**	-1.09(-1.97, -0.21)	-0.07(-0.94, 0.78)	-0.68(-1.66, 0.3)	-0.1(-1.17, 0.98)	0.19(-0.93, 1.32)	-0.82(-1.98, 0.35)	0.06(-1.03, 1.15)	-1.28(-2.36, -0.19)	**J**								
**L**	-1.02(-1.55, -0.47)	0(-0.5, 0.5)	-0.61(-1.29, 0.09)	-0.03(-0.83, 0.8)	0.26(-0.6, 1.15)	-0.75(-1.68, 0.21)	0.13(-0.69, 0.98)	-1.21(-2.03, -0.36)	0.07(-0.84, 1)	**L**							
**M**	-0.46(-0.99,	0.56(0.06	-0.05(-0.74,	0.53(-0.28,	0.82(-0.05,	-0.18(-1.12,	0.69(-0.13,	-0.65(-	0.63(-0.28,	0.57(-0.04,	**M**						

	0.09)	, 1.06)	0.65)	1.35)	1.71)	0.76)	1.54)	1.47, 0.2)	1.56)	1.15)							
**N**	-0.06(-0.76, 0.64)	0.96(0.28, 1.62)	0.35(-0.47, 1.17)	0.93(0, 1.86)	1.22(0.24, 2.21)	0.21(-0.82, 1.25)	1.09(0.14, 2.04)	-0.25(-1.19, 0.7)	1.03(0.01, 2.05)	0.96(0.2, 1.7)	0.4(-0.35, 1.14)	**N**					
**P**	-1.21(-1.96, -0.46)	-0.19(-0.93, 0.52)	-0.8(-1.67, 0.06)	-0.22(-1.19, 0.74)	0.07(-0.95, 1.09)	-0.94(-2.01, 0.14)	-0.06(-1.04, 0.92)	-1.4(-2.38, -0.41)	-0.12(-1.17, 0.93)	-0.19(-1, 0.59)	-0.75(-1.55, 0.03)	-1.15(-2.06, -0.24)	**P**				
**Q**	-0.15(-0.97, 0.67)	0.87(0.06, 1.65)	0.26(-0.67, 1.18)	0.84(-0.18, 1.86)	1.13(0.06, 2.21)	0.12(-1, 1.24)	1(-0.04, 2.03)	-0.34(-1.38, 0.7)	0.94(-0.16, 2.04)	0.87(-0.01, 1.71)	0.3(-0.56, 1.16)	-0.09(-1.06, 0.87)	1.06(0.06, 2.06)	**Q**			
**S**	-0.15(-1.37, 1.07)	0.87(-0.34, 2.06)	0.26(-1.04, 1.55)	0.84(-0.52, 2.2)	1.13(-0.27, 2.53)	0.12(-1.32, 1.57)	1(-0.37, 2.37)	-0.34(-1.71, 1.04)	0.94(-0.49, 2.38)	0.87(-0.39, 2.11)	0.31(-0.94, 1.55)	-0.09(-1.42, 1.23)	1.06(-0.29, 2.41)	0(-1.38, 1.39)	**S**		
**T**	-0.15(-1.14, 0.84)	0.87(-0.11, 1.83)	0.26(-0.82, 1.34)	0.84(-0.32, 2)	1.13(-0.08, 2.34)	0.12(-1.13, 1.37)	1(-0.18, 2.17)	-0.34(-1.51, 0.84)	0.94(-0.3, 2.17)	0.87(-0.16, 1.88)	0.31(-0.72, 1.32)	-0.09(-1.21, 1.02)	1.06(-0.08, 2.2)	0(-1.19, 1.19)	0(-1.49, 1.5)	**T**	
**U**	-1.25(-1.59, -0.91)	-0.23(-0.51, 0.02)	-0.84(-1.39, -0.29)	-0.26(-0.96, 0.44)	0.03(-0.74, 0.8)	-0.98(-1.82, -0.14)	-0.1(-0.82, 0.62)	-1.44(-2.16, -0.72)	-0.16(-0.98, 0.65)	-0.23(-0.67, 0.18)	-0.79(-1.22, -0.38)	-1.19(-1.81, -0.58)	-0.04(-0.71, 0.63)	-1.1(-1.84, -0.35)	-1.1(-2.27, 0.08)	-1.1(-2.03, -0.17)	**U**

Footnote. Results were mean change (95% CrI) from the network meta-analysis. Mean difference values less than 0 indicates that the listed intervention was better than the row intervention. **A**, *S. boulardii*; **B**, LGG; **C**, *L. reuteri*; **D**, *Bacillus clausii*; **E**, *L. acidophilus*; **F**, *B. lactis*; **G**, *L. sporogenes*; **I**, *ECN 1917*; **J**, *L. paracasei*; **L**, *L.* spp; **M**, *L.* spp + *B.* spp; **N**, *L.* spp + *B.* spp+ *S.* spp; **P**, *B.* spp + *S.* spp; **Q**, *Bacillus* spp + *E.* spp + C. spp; **S,** L. spp + B. spp + *P.* spp; **T**, *L.* spp + *S.* spp + *C.* spp + *Bacillus* spp; **U**, Control (placebo).

Table S13. NMA results for the duration of diarrhea (control = no treatment)

**A**	**A**												
**B**	0.62 (-0.04, 1.27)	**B**											
**C**	0.03 (-0.97, 1.04)	-0.58 (-1.68, 0.51)	**C**										
**D**	-0.64 (-1.26, -0.02)	-1.26 (-2.03, -0.49)	-0.67 (-1.78, 0.43)	**D**									
**F**	2.22 (0.92, 3.52)	1.6 (0.19, 3.01)	2.18 (0.58, 3.79)	2.86 (1.45, 4.26)	**F**								
**H**	0.28 (-1.09, 1.65)	-0.34 (-1.77, 1.09)	0.25 (-1.37, 1.86)	0.92 (-0.52, 2.36)	-1.94 (-3.78, -0.09)	**H**							
**K**	-0.94 (-2.04, 0.15)	-1.55 (-2.71, -0.41)	-0.97 (-2.41, 0.45)	-0.3 (-1.44, 0.85)	-3.16 (-4.83, -1.49)	-1.22 (-2.92, 0.48)	**K**						
**M**	0.02 (-0.69, 0.72)	-0.6 (-1.42, 0.22)	-0.02 (-1.13, 1.1)	0.66 (-0.18, 1.5)	-2.2 (-3.63, -0.77)	-0.26 (-1.71, 1.18)	0.95 (-0.28, 2.19)	**M**					
**N**	0.31 (-0.5, 1.1)	-0.31 (-1.2, 0.57)	0.28 (-0.93, 1.46)	0.95 (0.05, 1.83)	-1.91 (-3.41, -0.43)	0.03 (-1.49, 1.53)	1.25 (0.04, 2.44)	0.29 (-0.67, 1.23)	**N**				
**O**	-0.22 (-1.64, 1.2)	-0.84 (-2.32, 0.64)	-0.25 (-1.91, 1.4)	0.42 (-1.07, 1.9)	-2.44 (-4.33, -0.56)	-0.5 (-2.39, 1.39)	0.72 (-1.02, 2.46)	-0.24 (-1.73, 1.25)	-0.53 (-2.07, 1.03)	**O**			
**Q**	0.15 (-1.25, 1.54)	-0.47 (-1.93, 0.99)	0.12 (-1.52, 1.75)	0.79 (-0.68, 2.25)	-2.07 (-3.94, -0.2)	-0.13 (-2.01, 1.75)	1.09 (-0.64, 2.81)	0.13 (-1.34, 1.6)	-0.16 (-1.69, 1.39)	0.37 (-1.54, 2.28)	**Q**		
**R**	0.58 (-0.8, 1.95)	-0.04 (-1.47, 1.39)	0.55 (-1.07, 2.16)	1.22 (-0.22, 2.67)	-1.64 (-3.49, 0.21)	0.3 (-1.55, 2.16)	1.52 (-0.18, 3.22)	0.56 (-0.89, 2.01)	0.27 (-1.24, 1.8)	0.8 (-1.1, 2.7)	0.43 (-1.45, 2.31)	**R**	
**U**	-0.95 (-1.33, -0.58)	-1.57 (-2.13, -1.01)	-0.98 (-1.92, -0.04)	-0.31 (-0.9, 0.28)	-3.17 (-4.47, -1.87)	-1.23 (-2.55, 0.08)	-0.01 (-1.09, 1.06)	-0.97 (-1.57, -0.37)	-1.26 (-1.99, -0.51)	-0.73 (-2.1, 0.64)	-1.1 (-2.44, 0.24)	-1.53 (-2.85, -0.21)	**U**

Footnote. Results were mean change (95% CrI) from the network meta-analysis. Mean difference values less than 0 indicates that the listed intervention was better than the row intervention. **A**, *S. boulardii*; **B**, LGG; **C**, *L. reuteri*; **D**, *Bacillus clausii*; **F**, *B. lactis*; **H**, *L. plantarum*; **K**, *E. faecium*; **M**, *L.* spp + *B.* spp; **N**, *L.* spp + *B.* spp+ *S.* spp; **O**, *L.* spp + *S.* spp; **Q**, *Bacillus* spp + *E.* spp + C. spp; **R**, *L.* spp + *B.* spp + *E.* spp; **U**, Control (no treatment).

Table S14. NMA results for diarrhea lasting ≥2 days

**A**	**A**											
**B**	0.39(0.12, 1.20)	**B**										
**C**	0.94(0.29, 2.9)	2.4(0.62, 9.1)	**C**									
**D**	0.67(0.047, 12.0)	1.7(0.11, 33.0)	0.72(0.046, 14.0)	**D**								
**E**	0.26(0.031, 2.1)	0.67(0.072, 6.0)	0.27(0.030, 2.5)	0.38(0.013, 10.0)	**E**							
**I**	0.44(0.050, 3.8)	1.1(0.11, 11.0)	0.46(0.048, 4.6)	0.65(0.020, 18.0)	1.7(0.095, 30.0)	**I**						
**L**	0.21(0.027, 1.7)	0.55(0.061, 4.8)	0.23(0.026, 2.0)	0.31(0.011, 8.1)	0.82(0.050, 14.0)	0.48(0.028, 8.4)	**L**					
**M**	1.1(0.24, 4.8)	2.8(0.54, 14.0)	1.1(0.22, 6.0)	1.6(0.072, 29.0)	4.2(0.38, 47.0)	2.5(0.21, 29.0)	5.0(0.47, 55.0)	**M**				
**N**	0.62(0.17, 2.3)	1.6(0.37, 6.9)	0.66(0.16, 2.9)	0.93(0.046, 15.0)	2.4(0.25, 24.0)	1.4(0.14, 15.0)	2.9(0.31, 28.0)	0.58(0.10, 3.3)	**N**			
**O**	0.82(0.088, 7.6)	2.1(0.20, 22.0)	0.87(0.086, 9.2)	1.2(0.037, 35.0)	3.2(0.17, 59.0)	1.9(0.096, 37.0)	3.9(0.21, 70.0)	0.77(0.061, 9.5)	1.3(0.12, 14.0)	**O**		
**R**	1.3(0.14, 13.0)	3.4(0.32, 36.0)	1.4(0.14, 15.0)	1.9(0.058, 56.0)	5.1(0.27, 97.0)	3.0(0.15, 61.0)	6.2(0.34, 200)	1.2(0.097, 16.0)	2.1(0.19, 24.0)	1.6(0.078, 34.0)	**R**	
**U**	0.22(0.11, 0.41)	0.56(0.21, 1.4)	0.23(0.090, 0.60)	0.32(0.020, 4.2)	0.84(0.11, 6.2)	0.50(0.062, 4.0)	1.0(0.14, 7.3)	0.20(0.052, 0.77)	0.35(0.11, 1.0)	0.26(0.031, 2.2)	0.16(0.019, 1.4)	**U**

Footnote. Results are Odds Ratio (95% CrI) from the network meta-analysis. Odds ratios < 1 indicates that the listed intervention is better than the row intervention. **A**, *S. boulardii*; **B**, LGG; **C**, *L. reuteri*; **D**, *Bacillus clausii*; **E**, *L. acidophilus*; **I**, *ECN 1917*; **L**, *L.* spp; **M**, *L.* spp + *B.* spp; **N**, *L.* spp + *B.* spp+ *S.* spp; **O**, *L.* spp + *S.* spp; **P**, *B.* spp + *S.* spp; **R**, *L.* spp + *B.* spp + *E.* spp; **U**, Control (placebo/no treatment).

Table S15. NMA results for the duration of hospitalization

**A**	**A**											
**B**	0.33 (-0.79, 1.46)	**B**										
**C**	-0.19 (-1.7, 1.32)	-0.51 (-2.12, 1.08)	**C**									
**D**	-0.53 (-1.6, 0.55)	-0.85 (-2.19, 0.48)	-0.34 (-2.01, 1.34)	**D**								
**E**	-0.6 (-2.09, 0.89)	-0.92 (-2.52, 0.66)	-0.41 (-2.29, 1.47)	-0.07 (-1.73, 1.59)	**E**							
**H**	-1.11 (-3.15, 0.94)	-1.44 (-3.55, 0.68)	-0.92 (-3.27, 1.44)	-0.58 (-2.75, 1.59)	-0.51 (-2.84, 1.82)	**H**						
**L**	0.22 (-1.86, 2.3)	-0.11 (-2.27, 2.04)	0.41 (-1.97, 2.78)	0.74 (-1.46, 2.96)	0.82 (-1.55, 3.19)	1.33 (-1.42, 4.08)	**L**					
**M**	-0.22 (-1.32, 0.87)	-0.55 (-1.78, 0.66)	-0.04 (-1.61, 1.54)	0.3 (-1.01, 1.62)	0.38 (-1.19, 1.94)	0.89 (-1.21, 2.98)	-0.44 (-2.57, 1.69)	**M**				
**N**	-0.14 (-2.21, 1.93)	-0.47 (-2.62, 1.68)	0.05 (-2.32, 2.41)	0.38 (-1.81, 2.58)	0.46 (-1.9, 2.82)	0.97 (-1.78, 3.7)	-0.36 (-3.12, 2.42)	0.08 (-2.05, 2.21)	**N**			
**Q**	-0.42 (-1.96, 1.09)	-0.75 (-2.38, 0.85)	-0.23 (-2.15, 1.66)	0.11 (-1.59, 1.79)	0.18 (-1.73, 2.07)	0.69 (-1.69, 3.03)	-0.64 (-3.03, 1.74)	-0.2 (-1.8, 1.39)	-0.28 (-2.68, 2.09)	**Q**		

**R**	-0.05 (-2.07, 1.98)	-0.38 (-2.47, 1.71)	0.14 (-2.19, 2.47)	0.47 (-1.67, 2.63)	0.54 (-1.77, 2.87)	1.06 (-1.64, 3.75)	-0.27 (-3, 2.47)	0.17 (-1.9, 2.25)	0.09 (-2.64, 2.81)	0.37 (-1.95, 2.71)	**R**	
**U**	-0.88 (-1.58, -0.18)	-1.21 (-2.09, -0.33)	-0.69 (-2.02, 0.65)	-0.35 (-1.36, 0.65)	-0.28 (-1.6, 1.04)	0.23 (-1.69, 2.15)	-1.1 (-3.06, 0.86)	-0.65 (-1.5, 0.19)	-0.74 (-2.69, 1.22)	-0.46 (-1.8, 0.91)	-0.83 (-2.73, 1.07)	**U**

Footnote. Results were mean change (CI) from the network meta-analysis. Mean difference values less than 0 indicates that the listed intervention was better than the row intervention. **A**, *S. boulardii*; **B**, LGG; **C**, *L. reuteri*; **D**, *Bacillus clausii*; **E**, *L. acidophilus*; **H**, *L. plantarum*; **L**, *L.* spp; **M**, *L.* spp + *B.* spp; **N**, *L.* spp + *B.* spp+ *S.* spp; **Q**, *Bacillus* spp + *E.* spp + C. spp; **R**, *L.* spp + *B.* spp + *E.* spp; **U**, Control (placebo/no treatment).

Table S16. NMA results for the mean stool frequency on day 2

**A**	**A**												
**B**	-0.0077(-0.64, 0.67)	**B**											
**C**	0.79(-0.15, 1.7)	0.80(-0.21, 1.8)	**C**										
**D**	-0.27(-0.91, 0.40)	-0.26(-1.0, 0.47)	-1.1(-2.1, -0.033)	**D**									
**E**	-0.67(-2.6, 1.2)	-0.66(-2.6, 1.3)	-1.5(-3.5, 0.58)	-0.40(-2.3, 1.9)	**E**								
**K**	-0.44(-1.5, 0.63)	-0.43(-1.5, 0.64)	-1.2(-2.5, 0.10)	-0.18(-1.3, 0.92)	0.23(-1.9, 2.3)	**K**							
**L**	-0.44(-1.4,	-0.44(-1.5,	-1.2(-2.5, -	-0.18(-1.3,	0.22(-1.9,	-0.0017(-	**L**						

	0.55)	0.59)	0.0054)	0.88)	2.3)	1.4, 1.3)							
**M**	0.12(-0.74, 1.0)	0.12(-0.82, 1.0)	-0.68(-1.8, 0.48)	0.38(-0.57, 1.4)	0.78(-1.2, 2.8)	0.56(-0.72, 1.8)	0.56(-0.60, 1.8)	**M**					
**N**	0.11(-0.71, 0.94)	0.12(-0.76, 0.96)	-0.68(-1.8, 0.46)	0.38(-0.51, 1.3)	0.78(-1.2, 2.8)	0.55(-0.60, 1.7)	0.55(-0.60, 1.7)	-0.0047(-1.1, 1.1)	**N**				
**Q**	0.98(-0.29, 2.3)	0.99(-0.35, 2.3)	0.19(-1.3, 1.7)	1.3(-0.094, 2.6)	1.6(-0.56, 3.9)	1.4(-0.16, 3.0)	1.4(-0.068, 3.0)	0.87(-0.58, 2.3)	0.87(-0.56, 2.3)	**Q**			
**R**	0.61(-0.80, 2.0)	0.61(-0.85, 2.1)	-0.18(-1.8, 1.4)	0.87(-0.59, 2.3)	1.3(-1.0, 3.6)	1.1(-0.65, 2.7)	1.1(-0.56, 2.7)	0.49(-1.1, 2.0)	0.50(-1.0, 2.0)	-0.38(-2.2, 1.4)	**R**		
**T**	0.19(-1.5, 1.9)	0.19(-1.5, 1.9)	-0.60(-2.4, 1.2)	0.45(-1.3, 2.2)	0.85(-1.6, 3.3)	0.63(-1.3, 2.6)	0.63(-1.2, 2.5)	0.069(-1.7, 1.9)	0.074(-1.7, 1.9)	-0.80(-2.8, 1.2)	-0.42(-2.5, 1.7)	**T**	
**U**	-0.66(-1.1, -0.23)	-0.66(-1.2, -0.14)	-1.5(-2.3, -0.61)	-0.40(-0.98, 0.18)	0.0018(-1.9, 1.9)	-0.22(-1.3, 0.80)	-0.22(-1.1, 0.70)	-0.78(-1.6, -0.021)	-0.77(-1.5, -0.014)	-1.6(-2.9, -0.44)	-1.3(-2.6, 0.073)	-0.85(-2.5, 0.77)	**U**

Footnote. Results were mean change (95% CrI) from the network meta-analysis. Mean difference values less than 0 indicates that the listed intervention was better than the row intervention. **A**, *S. boulardii*; **B**, LGG; **C**, *L. reuteri*; **D**, *Bacillus clausii*; **E**, *L. acidophilus*; **K**, *E. faecium*; **L**, *L.* spp; **M**, *L.* spp + *B.* spp; **N**, *L.* spp + *B.* spp+ *S.* spp; **Q**, *Bacillus* spp + *E.* spp + C. spp; **R**, *L.* spp + *B.* spp + *E.* spp; **T**, *L.* spp + *S.* spp + *C.* spp + *Bacillus* spp; **U**, Control (placebo/no treatment).

Table S17. NMA results for the duration of vomiting

**A**	**A**								
**B**	0.09 (-0.23, 0.44)	**B**							
**D**	0.05 (-0.46, 0.57)	-0.04 (-0.56, 0.47)	**D**						
**H**	0.01 (-0.78, 0.82)	-0.08 (-0.88, 0.72)	-0.04 (-0.95, 0.87)	**H**					
**K**	0.05 (-0.45, 0.56)	-0.04 (-0.55, 0.46)	0 (-0.58, 0.58)	0.04 (-0.86, 0.94)	**K**				
**L**	0.17 (-0.43, 0.78)	0.08 (-0.53, 0.68)	0.12 (-0.62, 0.86)	0.16 (-0.78, 1.1)	0.12 (-0.61, 0.85)	**L**			
**N**	0.19 (-0.31, 0.71)	0.1 (-0.41, 0.6)	0.14 (-0.44, 0.72)	0.18 (-0.73, 1.08)	0.14 (-0.43, 0.71)	0.02 (-0.71, 0.75)	**N**		
**Q**	0.56 (0, 1.15)	0.47 (-0.11, 1.04)	0.51 (-0.21, 1.23)	0.55 (-0.37, 1.47)	0.51 (-0.2, 1.22)	0.39 (-0.36, 1.15)	0.37 (-0.34, 1.08)	**Q**	
**S**	1.14 (-0.2, 2.47)	1.04 (-0.29, 2.37)	1.09 (-0.31, 2.48)	1.13 (-0.4, 2.64)	1.09 (-0.31, 2.48)	0.96 (-0.45, 2.39)	0.94 (-0.45, 2.33)	0.57 (-0.84, 1.98)	**S**
**U**	-0.07 (-0.31, 0.19)	-0.16 (-0.42, 0.09)	-0.12 (-0.61, 0.38)	-0.08 (-0.84, 0.68)	-0.12 (-0.6, 0.36)	-0.24 (-0.79, 0.31)	-0.26 (-0.75, 0.23)	-0.63 (-1.15, -0.11)	-1.2 (-2.51, 0.11)	**U**

Footnote. Results were mean change (95% CrI) from the network meta-analysis. Mean difference values less than 0 indicates that the listed intervention was better than the row intervention. **A**, *S. boulardii*; **B**, LGG; **D**, *Bacillus clausii*; **H**, *L. plantarum*; **K**, *E. faecium*; **L**, *L.* spp; **N**, *L.* spp + *B.* spp+ *S.* spp; **Q**, *Bacillus* spp + *E.* spp + C. spp; **S,** L. spp + B. spp + *P.* spp; **U**, Control (placebo/no treatment).

Table S18. NMA results for the duration of fever

**A**	**A**											
**B**	0.37 (-0.29, 1.02)	**B**										
**D**	-0.09 (-0.55, 0.36)	-0.46 (-1.17, 0.25)	**D**									
**F**	0.33 (-0.52, 1.19)	-0.04 (-1.05, 0.99)	0.42 (-0.46, 1.32)	**F**								
**H**	0.05 (-1.85, 1.97)	-0.32 (-2.3, 1.68)	0.14 (-1.78, 2.08)	-0.29 (-2.31, 1.76)	**H**							
**K**	-0.27 (-1.05, 0.5)	-0.64 (-1.51, 0.24)	-0.18 (-1, 0.64)	-0.6 (-1.71, 0.49)	-0.32 (-2.36, 1.71)	**K**						
**L**	0.02 (-1.12, 1.16)	-0.35 (-1.61, 0.92)	0.11 (-1.05, 1.27)	-0.31 (-1.65, 1.02)	-0.03 (-2.2, 2.14)	0.29 (-1.04, 1.63)	**L**					
**M**	0.47 (-0.63, 1.58)	0.1 (-1.13, 1.34)	0.56 (-0.56, 1.7)	0.14 (-1.17, 1.45)	0.42 (-1.74, 2.57)	0.74 (-0.56, 2.05)	0.45 (-1.06, 1.97)	**M**				
**N**	-0.06 (-0.69, 0.57)	-0.43 (-1.22, 0.37)	0.03 (-0.64, 0.71)	-0.4 (-1.38, 0.59)	-0.11 (-2.1, 1.85)	0.21 (-0.68, 1.11)	-0.08 (-1.31, 1.16)	-0.53 (-1.74, 0.67)	**N**			
**Q**	-0.06 (-0.72, 0.56)	-0.44 (-1.29, 0.41)	0.02 (-0.67, 0.7)	-0.4 (-1.37, 0.54)	-0.11 (-2.08, 1.83)	0.2 (-0.75, 1.14)	-0.08 (-1.3, 1.12)	-0.54 (-1.73, 0.64)	0 (-0.82, 0.78)	**Q**		
**S**	0.31 (-1.19, 1.81)	-0.06 (-1.65, 1.54)	0.4 (-1.12, 1.93)	-0.02 (-1.68, 1.64)	0.26 (-2.13, 2.65)	0.58 (-1.07, 2.24)	0.29 (-1.54, 2.14)	-0.16 (-1.96, 1.65)	0.37 (-1.21, 1.95)	0.38 (-1.18, 1.95)	**S**	
**U**	-0.18 (-0.53, 0.16)	-0.56 (-1.2, 0.11)	-0.1 (-0.5, 0.33)	-0.52 (-1.3, 0.27)	-0.23 (-2.13, 1.64)	0.08 (-0.68, 0.86)	-0.2 (-1.29, 0.89)	-0.66 (-1.7, 0.39)	-0.12 (-0.71, 0.47)	-0.12 (-0.65, 0.44)	-0.5 (-1.97, 0.97)	**U**

Footnote. Results were mean change (95% CrI) from the network meta-analysis. Mean difference values less than 0 indicates that the listed intervention was better than the row intervention. **A**, *S. boulardii*; **B**, LGG; **D**, *Bacillus clausii*; **F**, *B. lactis*; **H**, *L. plantarum*; **K**, *E. faecium*; **L**, *L.* spp; **M**, *L.* spp + *B.* spp; **N**, *L.* spp + *B.* spp+ *S.* spp; **Q**, *Bacillus* spp + *E.* spp + C. spp; **S,** L. spp + B. spp + *P.* spp; **U**, Control (placebo/no treatment).

Table S19. Certainty of evidence for the duration of diarrhea

Comparison	N of trials	Certainty of evidence	Imprecision	Indirectness	Inconsistency	Overall risk of bias
A vs B	1	Very low	Serious	Serious	Very serious	Not serious
A vs D	3	Moderate	Serious	Not serious	Not serious	Not serious
A vs F	1	Very low	Serious	Serious	Very serious	Serious
A vs K	1	Moderate	Serious	Not serious	Not serious	Not serious
A vs N	1	Moderate	Serious	Not serious	Not serious	Not serious
A vs U	20	Low	Not serious	Not serious	Serious	Serious
B vs D	1	Moderate	Serious	Not serious	Not serious	Not serious
B vs K	1	Moderate	Serious	Not serious	Not serious	Not serious
B vs N	1	Moderate	Serious	Not serious	Not serious	Not serious
B vs U	15	Low	Not serious	Not serious	Serious	Serious
C vs U	6	Moderate	Not serious	Not serious	Not serious	Serious
D vs K	1	Moderate	Serious	Not serious	Not serious	Not serious
D vs N	1	Moderate	Serious	Not serious	Not serious	Not serious
D vs U	4	Low	Not serious	Not serious	Serious	Serious
E vs U	1	Moderate	Serious	Not serious	Not serious	Not serious
F vs U	2	Low	Serious	Not serious	Serious	Not serious
G vs U	1	Moderate	Serious	Not serious	Not serious	Not serious
H vs U	1	Low	Serious	Not serious	Not serious	Serious
I vs U	1	Very low	Very serious	Not serious	Not serious	Serious
J vs U	1	Moderate	Serious	Not serious	Not serious	Not serious
K vs N	1	Moderate	Serious	Not serious	Not serious	Not serious
K vs U	1	Moderate	Serious	Not serious	Not serious	Not serious
L vs U	6	High	Not serious	Not serious	Not serious	Not serious
M vs U	8	Low	Not serious	Not serious	Serious	Serious
N vs U	5	Moderate	Not serious	Not serious	Not serious	Serious
O vs U	1	Low	Serious	Not serious	Not serious	Serious
P vs U	1	Low	Serious	Not serious	Not serious	Serious
Q vs U	2	Low	Serious	Not serious	Not serious	Serious
R vs U	1	Low	Serious	Not serious	Not serious	Serious
S vs U	1	Low	Serious	Not serious	Not serious	Serious
T vs U	1	Low	Serious	Not serious	Not serious	Serious

Footnote. **A**, *S. boulardii*; **B**, LGG; **C**, *L. reuteri*; **D**, *Bacillus clausii*; **E**, *L. acidophilus*; **F**, *B. lactis*; **G**, *L. sporogenes*; **H**, *L. plantarum*; **I**, *ECN 1917*; **J**, *L. paracasei*; **K**, *E. faecium*; **L**, *L.* spp; **M**, *L.* spp + *B.* spp; **N**, *L.* spp + *B.* spp+ *S.* spp; **O**, *L.* spp + *S.* spp; **P**, *B.* spp + *S.* spp; **Q**, *Bacillus* spp + *E.* spp + C. spp; **R**, *L.* spp + *B.* spp + *E.* spp; **S,** L. spp + B. spp + *P.* spp; **T**, *L.* spp + *S.* spp + *C.* spp + *Bacillus* spp; **U**,

Control (placebo/no treatment).

Table S20. Certainty of evidence for the duration of diarrhea (control = placebo)

Comparison	N of trials	Certainty of evidence	Imprecision	Indirectness	Inconsistency	Overall risk of bias
A vs U	6	Moderate	Not serious	Not serious	Serious	Not serious
B vs U	10	Moderate	Not serious	Not serious	Serious	Not serious
C vs U	4	Moderate	Serious	Not serious	Not serious	Not serious
D vs U	1	Moderate	Serious	Not serious	Not serious	Not serious
E vs U	1	Moderate	Serious	Not serious	Not serious	Not serious
F vs U	1	Moderate	Serious	Not serious	Not serious	Not serious
G vs U	1	Moderate	Serious	Not serious	Not serious	Not serious
I vs U	1	Very low	Very serious	Not serious	Not serious	Serious
J vs U	1	Moderate	Serious	Not serious	Not serious	Not serious
L vs U	6	High	Not serious	Not serious	Not serious	Not serious
M vs U	4	Low	Serious	Not serious	Serious	Not serious
N vs U	2	Moderate	Serious	Not serious	Not serious	Not serious
P vs U	1	Low	Serious	Not serious	Not serious	Serious
Q vs U	1	Moderate	Serious	Not serious	Not serious	Not serious
S vs U	1	Low	Serious	Not serious	Not serious	Serious
T vs U	1	Low	Serious	Not serious	Not serious	Serious

Footnote. **A**, *S. boulardii*; **B**, LGG; **C**, *L. reuteri*; **D**, *Bacillus clausii*; **E**, *L. acidophilus*; **F**, *B. lactis*; **G**, *L. sporogenes*; **I**, *ECN 1917*; **J**, *L. paracasei*; **L**, *L.* spp; **M**, *L.* spp + *B.* spp; **N**, *L.* spp + *B.* spp+ *S.* spp; **P**, *B.* spp + *S.* spp; **Q**, *Bacillus* spp + *E.* spp + C. spp; **S,** L. spp + B. spp + *P.* spp; **T**, *L.* spp + *S.* spp + *C.* spp + *Bacillus* spp; **U**, Control (placebo).

Table S21. Certainty of evidence for the duration of diarrhea (control = no treatment)

Comparison	N of trials	Certainty of evidence	Imprecision	Indirectness	Inconsistency	Overall risk of bias
A vs B	1	Moderate	Serious	Not serious	Not serious	Not serious
A vs D	3	Moderate	Serious	Not serious	Not serious	Not serious
A vs F	1	Low	Serious	Not serious	Not serious	Serious
A vs K	1	Moderate	Serious	Not serious	Not serious	Not serious
A vs N	1	Moderate	Serious	Not serious	Not serious	Not serious
A vs U	14	Low	Not serious	Not serious	Serious	Serious
B vs D	1	Moderate	Serious	Not serious	Not serious	Not serious
B vs K	1	Moderate	Serious	Not serious	Not serious	Not serious
B vs N	1	Moderate	Serious	Not serious	Not serious	Not serious
B vs U	5	Low	Not serious	Not serious	Serious	Serious

C vs U	2	Low	Serious	Not serious	Serious	Not serious
D vs K	1	Moderate	Serious	Not serious	Not serious	Not serious
D vs N	1	Moderate	Serious	Not serious	Not serious	Not serious
D vs U	2	Low	Serious	Not serious	Serious	Not serious
F vs U	1	Low	Serious	Not serious	Not serious	Serious
H vs U	1	Low	Serious	Not serious	Not serious	Serious
K vs N	1	Moderate	Serious	Not serious	Not serious	Not serious
K vs U	1	Moderate	Serious	Not serious	Not serious	Not serious
M vs U	4	Low	Not serious	Not serious	Serious	Serious
N vs U	3	Very low	Serious	Not serious	Serious	Serious
O vs U	1	Low	Serious	Not serious	Not serious	Serious
Q vs U	1	Low	Serious	Not serious	Not serious	Serious
R vs U	1	Low	Serious	Not serious	Not serious	Serious

Footnote. **A**, *S. boulardii*; **B**, LGG; **C**, *L. reuteri*; **D**, *Bacillus clausii*; **F**, *B. lactis*; **H**, *L. plantarum*; **K**, *E. faecium*; **M**, *L.* spp + *B.* spp; **N**, *L.* spp + *B.* spp+ *S.* spp; **O**, *L.* spp + *S.* spp; **Q**, *Bacillus* spp + *E.* spp + C. spp; **R**, *L.* spp + *B.* spp + *E.* spp; **U**, Control (no treatment).

Table S22. Certainty of evidence for diarrhea lasting ≥2 days

Comparison	N of trials	Certainty of evidence	Imprecision	Indirectness	Inconsistency	Overall risk of bias
A vs U	12	Moderate	Not serious	Not serious	Serious	Not serious
B vs U	5	Low	Serious	Not serious	Serious	Not serious
C vs U	6	Moderate	Not serious	Not serious	Serious	Not serious
D vs U	1	Low	Very serious	Not serious	Not serious	Not serious
E vs U	1	Low	Very serious	Not serious	Not serious	Not serious
I vs U	1	Very low	Very serious	Not serious	Not serious	Serious
L vs U	1	Moderate	Serious	Not serious	Not serious	Not serious
M vs U	3	Low	Serious	Not serious	Not serious	Serious
N vs U	4	Low	Serious	Not serious	Not serious	Serious
O vs U	1	Very low	Very serious	Not serious	Not serious	Serious
R vs U	1	Very low	Very serious	Not serious	Not serious	Serious

Footnote. **A**, *S. boulardii*; **B**, LGG; **C**, *L. reuteri*; **D**, *Bacillus clausii*; **E**, *L. acidophilus*; **I**, *ECN 1917*; **L**, *L.* spp; **M**, *L.* spp + *B.* spp; **N**, *L.* spp + *B.* spp+ *S.* spp; **O**, *L.* spp + *S.* spp; **P**, *B.* spp + *S.* spp; **R**, *L.* spp + *B.* spp + *E.* spp; **U**, Control (placebo/no treatment).

Table S23. Certainty of evidence for the duration of hospitalization

Comparison	N of trials	Certainty of evidence	Imprecision	Indirectness	Inconsistency	Overall risk of bias
A vs D	1	Moderate	Serious	Not serious	Not serious	Not serious
A vs U	7	Low	Not serious	Not serious	Serious	Serious
B vs U	5	Low	Not serious	Not serious	Serious	Serious
C vs U	2	Low	Serious	Not serious	Serious	Not serious
D vs U	3	Low	Not serious	Not serious	Serious	Serious
E vs U	2	Low	Serious	Not serious	Serious	Not serious
F vs U	1	Low	Serious	Not serious	Not serious	Serious
H vs U	1	Low	Serious	Not serious	Not serious	Serious
L vs U	1	Low	Serious	Not serious	Not serious	Serious
M vs U	5	Low	Not serious	Not serious	Serious	Serious
N vs U	1	Low	Serious	Not serious	Not serious	Serious
Q vs U	2	Low	Serious	Not serious	Serious	Not serious
R vs U	1	Low	Serious	Not serious	Not serious	Serious

Footnote. **A**, *S. boulardii*; **B**, LGG; **C**, *L. reuteri*; **D**, *Bacillus clausii*; **E**, *L. acidophilus*; **H**, *L. plantarum*; **L**, *L.* spp; **M**, *L.* spp + *B.* spp; **N**, *L.* spp + *B.* spp+ *S.* spp; **Q**, *Bacillus* spp + *E.* spp + C. spp; **R**, *L.* spp + *B.* spp + *E.* spp; **U**, Control (placebo/no treatment).

Table S24. Certainty of evidence for the mean stool frequency on day 2

Comparison	N of trials	Certainty of evidence	Imprecision	Indirectness	Inconsistency	Overall risk of bias
A vs B	1	Moderate	Serious	Not serious	Not serious	Not serious
A vs D	2	Low	Serious	Not serious	Not serious	Serious
A vs K	1	Moderate	Serious	Not serious	Not serious	Not serious
A vs N	1	Moderate	Serious	Not serious	Not serious	Not serious
A vs U	9	Moderate	Not serious	Not serious	Serious	Not serious
B vs D	1	Moderate	Serious	Not serious	Not serious	Not serious
B vs K	1	Moderate	Serious	Not serious	Not serious	Not serious
B vs N	1	Moderate	Serious	Not serious	Not serious	Not serious
B vs U	7	Moderate	Not serious	Not serious	Serious	Not serious
C vs U	4	Moderate	Serious	Not serious	Not serious	Not serious
D vs K	1	Moderate	Serious	Not serious	Not serious	Not serious
D vs U	4	High	Not serious	Not serious	Not serious	Not serious
D vs N	1	Moderate	Serious	Not serious	Not serious	Not serious
E vs U	1	Low	Serious	Not serious	Not serious	Serious
K vs N	1	Moderate	Serious	Not serious	Not serious	Not serious
K vs U	1	Moderate	Serious	Not serious	Not serious	Not serious
L vs U	2	Low	Serious	Not serious	Serious	Not serious
M vs U	3	Very low	Serious	Not serious	Serious	Serious
N vs U	3	Moderate	Serious	Not serious	Not serious	Not serious
Q vs U	1	Moderate	Serious	Not serious	Not serious	Not serious
R vs U	1	Low	Serious	Not serious	Not serious	Serious
T vs U	1	Low	Serious	Not serious	Not serious	Serious

Footnote. **A**, *S. boulardii*; **B**, LGG; **C**, *L. reuteri*; **D**, *Bacillus clausii*; **E**, *L. acidophilus*; **K**, *E. faecium*; **L**, *L.* spp; **M**, *L.* spp + *B.* spp; **N**, *L.* spp + *B.* spp+ *S.* spp; **Q**, *Bacillus* spp + *E.* spp + C. spp; **R**, *L.* spp + *B*. spp + *E*. spp; **T**, *L.* spp + *S.* spp + *C.* spp + *Bacillus* spp; **U**, Control (placebo/no treatment).

Table S25. Certainty of evidence for the duration of vomiting

Comparison	N of trials	Certainty of evidence	Imprecision	Indirectness	Inconsistency	Overall risk of bias
A vs B	1	Moderate	Serious	Not serious	Not serious	Not serious
A vs D	1	Moderate	Serious	Not serious	Not serious	Not serious
A vs K	1	Moderate	Serious	Not serious	Not serious	Not serious
A vs N	1	Moderate	Serious	Not serious	Not serious	Not serious
A vs U	4	Moderate	Not serious	Not serious	Serious	Not serious
B vs D	1	Moderate	Serious	Not serious	Not serious	Not serious
B vs K	1	Moderate	Serious	Not serious	Not serious	Not serious
B vs N	1	Moderate	Serious	Not serious	Not serious	Not serious
B vs U	4	High	Not serious	Not serious	Not serious	Not serious
D vs K	1	Moderate	Serious	Not serious	Not serious	Not serious
D vs N	1	Moderate	Serious	Not serious	Not serious	Not serious
D vs U	1	Moderate	Serious	Not serious	Not serious	Not serious
H vs U	1	Low	Serious	Not serious	Not serious	Serious
K vs N	1	Moderate	Serious	Not serious	Not serious	Not serious
K vs U	1	Moderate	Serious	Not serious	Not serious	Not serious
L vs U	2	Moderate	Serious	Not serious	Not serious	Not serious
N vs U	1	Moderate	Serious	Not serious	Not serious	Not serious
Q vs U	1	Moderate	Serious	Not serious	Not serious	Not serious
S vs U	1	Low	Serious	Not serious	Not serious	Serious

Footnote. **A**, *S. boulardii*; **B**, LGG; **D**, *Bacillus clausii*; **H**, *L. plantarum*; **K**, *E. faecium*; **L**, *L.* spp; **N**, *L.* spp + *B.* spp+ *S.* spp; **Q**, *Bacillus* spp + *E.* spp + C. spp; **S,** L. spp + B. spp + *P.* spp; **U**, Control (placebo/no treatment).

Table S26. Certainty of evidence for the duration of fever

Comparison	N of trials	Certainty of evidence	Imprecision	Indirectness	Inconsistency	Overall risk of bias
A vs B	1	Moderate	Serious	Not serious	Not serious	Not serious
A vs D	2	Moderate	Serious	Not serious	Not serious	Not serious
A vs K	1	Moderate	Serious	Not serious	Not serious	Not serious
A vs N	1	Moderate	Serious	Not serious	Not serious	Not serious
A vs U	4	Low	Not serious	Not serious	Serious	Serious
B vs D	1	Moderate	Serious	Not serious	Not serious	Not serious
B vs K	1	Moderate	Serious	Not serious	Not serious	Not serious
B vs N	1	Moderate	Serious	Not serious	Not serious	Not serious
B vs U	1	Moderate	Serious	Not serious	Not serious	Not serious
D vs K	1	Moderate	Serious	Not serious	Not serious	Not serious
D vs N	1	Moderate	Serious	Not serious	Not serious	Not serious
D vs U	3	Moderate	Serious	Not serious	Not serious	Not serious
F vs U	1	Low	Serious	Not serious	Not serious	Serious
H vs U	1	Low	Serious	Not serious	Not serious	Serious
K vs N	1	Moderate	Serious	Not serious	Not serious	Not serious
K vs U	1	Moderate	Serious	Not serious	Not serious	Not serious
L vs U	1	Low	Serious	Not serious	Not serious	Serious
M vs U	1	Low	Serious	Not serious	Not serious	Serious
N vs U	2	Moderate	Serious	Not serious	Not serious	Not serious
Q vs U	2	Moderate	Not serious	Not serious	Serious	Not serious
S vs U	1	Low	Serious	Not serious	Not serious	Serious

Footnote. **A**, *S. boulardii*; **B**, LGG; **D**, *Bacillus clausii*; **F**, *B. lactis*; **H**, *L. plantarum*; **K**, *E. faecium*; **L**, *L.* spp; **M**, *L.* spp + *B.* spp; **N**, *L.* spp + *B.* spp+ *S.* spp; **Q**, *Bacillus* spp + *E.* spp + C. spp; **S,** L. spp + B. spp + *P*. spp; **U**, Control (placebo/no treatment).

Table S27. Rank for outcomes

Intervention	Duration of diarrhea		Duration of diarrhea (control= placebo)		Duration of diarrhea (control= no treatment)		Diarrhea lasting ≥2 Days		Duration of hospitalization		Mean stool frequency on day 2		Duration of vomiting		Duration of fever	
	SUCRA	Rank	SUCRA	Rank	SUCRA	Rank	SUCRA	Rank	SUCRA	Rank	SUCRA	Rank	SUCRA	Rank	SUCRA	Rank
S. boulardii	0.636	**8**	0.831	**2**	0.475	**9**	0.721	**3**	0.659	**3**	0.515	**7**	0.320	**9**	0.481	**6**
LGG	0.515	**12**	0.303	**11**	0.779	**2**	0.361	**9**	0.789	**1**	0.510	**8**	0.467	**5**	0.751	**1**
L. reuteri	0.589	**10**	0.619	**8**	0.498	**7**	0.689	**4**	0.550	**6**	0.852	**2**	-	-	-	-
Bacillus clausii	0.271	**16**	0.315	**10**	0.195	**11**	0.549	**6**	0.390	**9**	0.357	**9**	0.394	**7**	0.383	**10**
L. acidophilus	0.210	**20**	0.168	**16**	-	-	0.272	**10**	0.372	**10**	0.261	**12**	-	-	-	-
B. lactis	0.951	**1**	0.682	**7**	0.991	**1**	-	-	-	-	-	-	-	-	0.703	**3**
L. sporogenes	0.249	**17**	0.228	**13**	-	-	-	-	-	-	-	-	-	-	-	-
L. plantarum	0.695	**5**	-	-	0.597	**5**	-	-	0.230	**11**	-	-	0.370	**8**	0.493	**5**
ECN 1917	0.768	**3**	0.886	**1**	-	-	0.425	**8**	-	-	-	-	-	-	-	-
L. paracasei	0.275	**15**	0.264	**14**	-	-	-	-	-	-	-	-	-	-	-	-
E. faecium	0.150	**22**	-	**-**	0.120	**12**	-	-	-	-	0.277	**10**	0.395	**6**	0.263	
L. spp	0.294	**14**	0.296	**12**	-	-	0.220	**11**	0.678	**2**	0.271	**11**	0.527	**4**	0.477	**7**
L. spp + *B.* spp	0.563	**11**	0.595	**9**	0.488	**8**	0.723	**2**	0.541	**7**	0.573	**5**	-	-	0.742	**2**
L. spp + *B.* spp+ *S.* spp	0.718	**4**	0.792	**3**	0.634	**4**	0.536	**7**	0.557	**5**	0.574	**4**	0.569	**3**	0.413	**8**

L. spp + *S*. spp	0.497	**13**	-	**-**	0.393	**10**	0.612	**5**	-	-	-	-	-	-	-	-
B. spp + *S*. spp	0.227	**18**	0.193	**15**	-	-	-	-	-	-	-	-	-	-	-	-
Bacillus spp + *E*. spp + C. spp(	0.666	**6**	0.742	**4**	0.544	**6**	-	-	0.448	**8**	0.878	**1**	0.844	**2**	0.411	**9**
L. spp + *B*. spp + *E*. spp	0.795	**2**	-	-	0.710	**3**	0.740	**1**	0.590	**4**	0.751	**3**	-	-	-	-
L. spp + *B*. spp + *S*. spp + *P*. spp	0.633	**9**	0.718	**6**	-	-	-	-	-	-	-	-	0.919	**1**	0.621	**4**
L. spp + *S*. spp + *C*. spp + *Bacillus* spp	0.642	**7**	0.733	**5**	-	-	-	-	-	-	0.571	**6**	-	-	-	-
Control (placebo/no treatment)	0.156	**21**	0.134	**17**	0.07	**13**	0.153	**12**	0.197	**12**	0.122	**13**	0.194	**10**	0.263	**11**
